# Supplementary material for: Cry Toxins Use Multiple ATP-Binding Cassette Transporter Subfamily C Members as Low-Efficiency Receptors in Bombyx mori
Source: Biomolecules. 2024 Feb 23;14(3):271. doi: 10.3390/biom14030271 (PMC10968512; doi:10.3390/biom14030271)
Supplement: Supplementary file 1 [file biomolecules-14-00271-s001.zip › Fig_S4.pdf]

Supplementary Figure 4. Domain prediction of BmABC transporters defined in this study using InterPro.

ABCA3

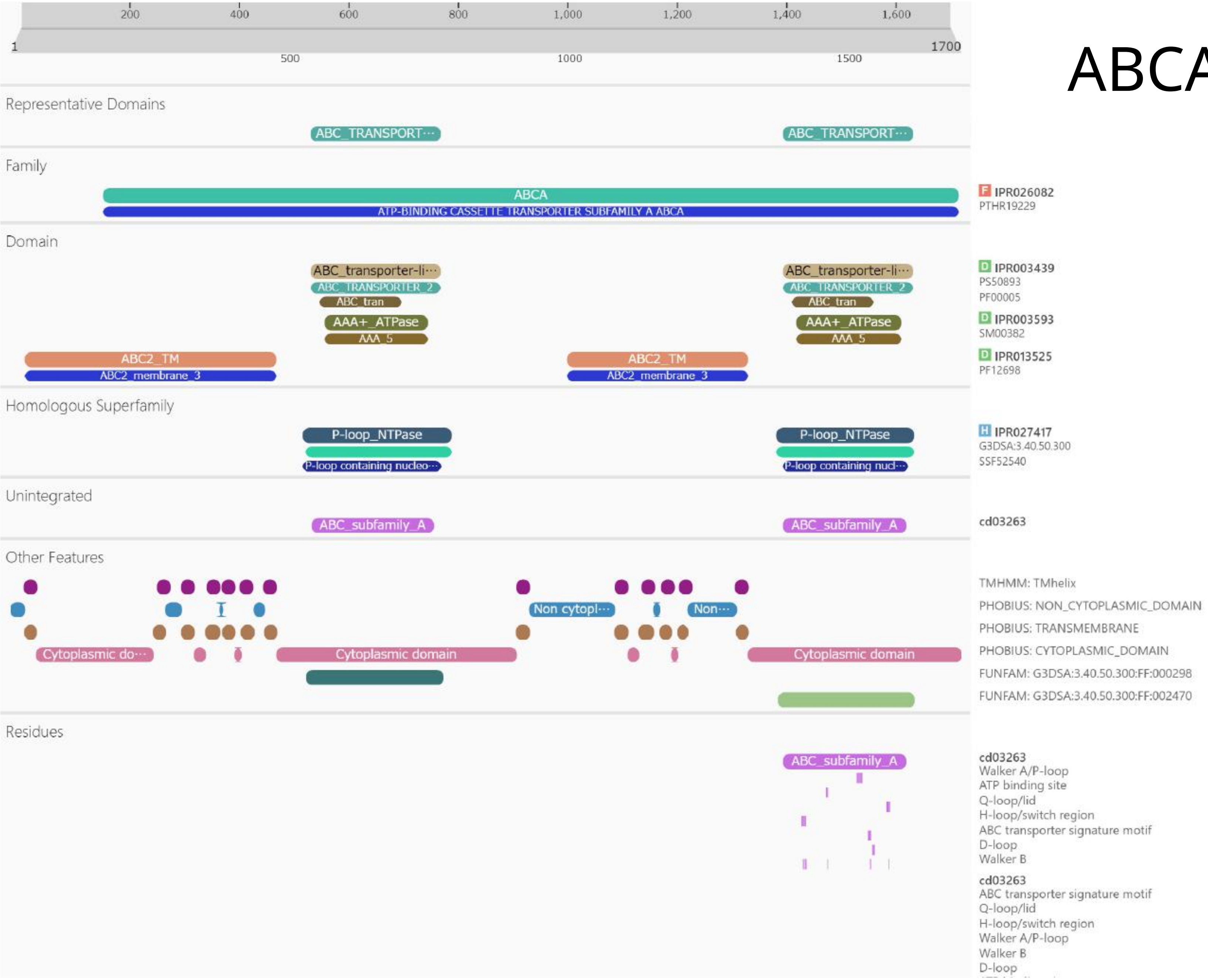

200
400
600
800
1,000
1,200
1,400
1,600
1
1700
500
1000
1500
Representative Domains
ABC_TRANSPORT···
ABC_TRANSPORT···
Family
ABCA
ATP-BINDING CASSETTE TRANSPORTER SUBFAMILY A ABCA
IPR026082
PTHR19229
Domain
ABC_transporter-li···
ABC_TRANSPORTER_2
ABC_tran
AAA+_ATPase
AAA_5
ABC2_TM
ABC2_membrane_3
IPR003439
PS50893
PF00005
IPR003593
SM00382
IPR013525
PF12698
Homologous Superfamily
P-loop_NTPase
P-loop containing nucleo···
P-loop containing nucl···
IPR027417
G3DSA:3.40.50.300
SSF52540
Unintegrated
ABC_subfamily_A
cd03263
Other Features
Non cytopl···
Non···
Cytoplasmic do···
Cytoplasmic domain
TMHMM: TMhelix
PHOBIUS: NON_CYTOPLASMIC_DOMAIN
PHOBIUS: TRANSMEMBRANE
PHOBIUS: CYTOPLASMIC_DOMAIN
FUNFAM: G3DSA:3.40.50.300:FF:000298
FUNFAM: G3DSA:3.40.50.300:FF:002470
Residues
ABC_subfamily_A
cd03263
Walker A/P-loop
ATP binding site
Q-loop/lid
H-loop/switch region
ABC transporter signature motif
D-loop
Walker B
cd03263
ABC transporter signature motif
Q-loop/lid
H-loop/switch region
Walker A/P-loop
Walker B
D-loop

ABCA2

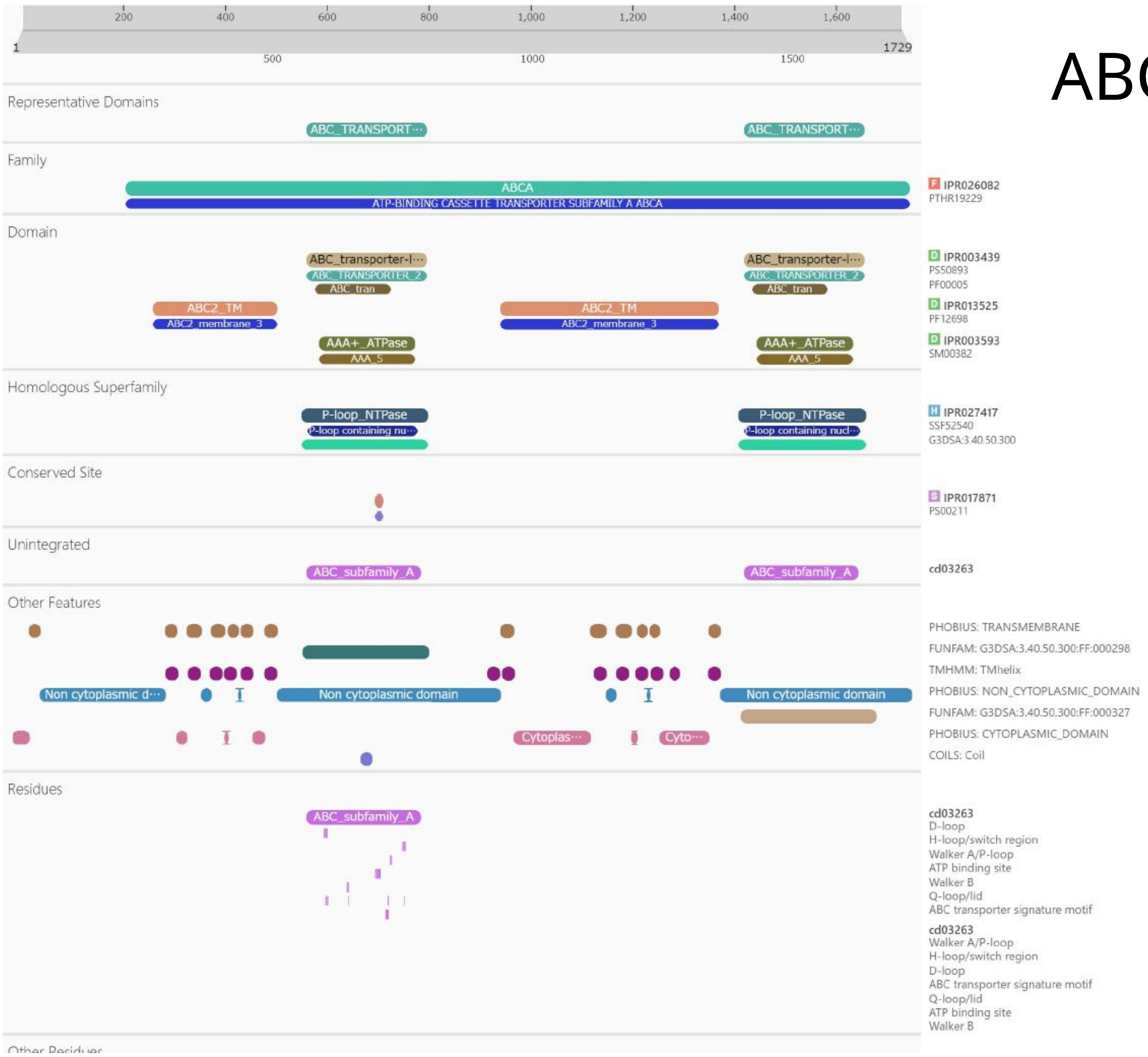
200
400
600
800
1,000
1,200
1,400
1,600
1
1729
500
1000
1500
Representative Domains
ABC_TRANSPORT···
ABC_TRANSPORT···
Family
ABCA
ATP-BINDING CASSETTE TRANSPORTER SUBFAMILY A ABCA
IPR026082
PTHR19229
Domain
ABC_transporter-l···
ABC_TRANSPORTER_2
ABC_tran
ABC2_TM
ABC2_membrane_3
AAA+_ATPase
AAA_5
IPR003439
PS50893
PF00005
IPR013525
PF12698
IPR003593
SM00382
Homologous Superfamily
P-loop_NTPase
P-loop containing nu···
IPR027417
SSF52540
G3DSA:3.40.50.300
Conserved Site
IPR017871
PS00211
Unintegrated
ABC_subfamily_A
cd03263
Other Features
Non cytoplasmic d···
Non cytoplasmic domain
Cytoplas···
Cyto···
PHOBIUS: TRANSMEMBRANE
FUNFAM: G3DSA:3.40.50.300:FF:000298
TMHMM: TMhelix
PHOBIUS: NON_CYTOPLASMIC_DOMAIN
FUNFAM: G3DSA:3.40.50.300:FF:000327
PHOBIUS: CYTOPLASMIC_DOMAIN
COILS: Coil
Residues
ABC_subfamily_A
cd03263
D-loop
H-loop/switch region
Walker A/P-loop
ATP binding site
Walker B
Q-loop/lid
ABC transporter signature motif
cd03263
Walker A/P-loop
H-loop/switch region
D-loop
ABC transporter signature motif
Q-loop/lid
ATP binding site
Walker B
Other Residues

ABCA4

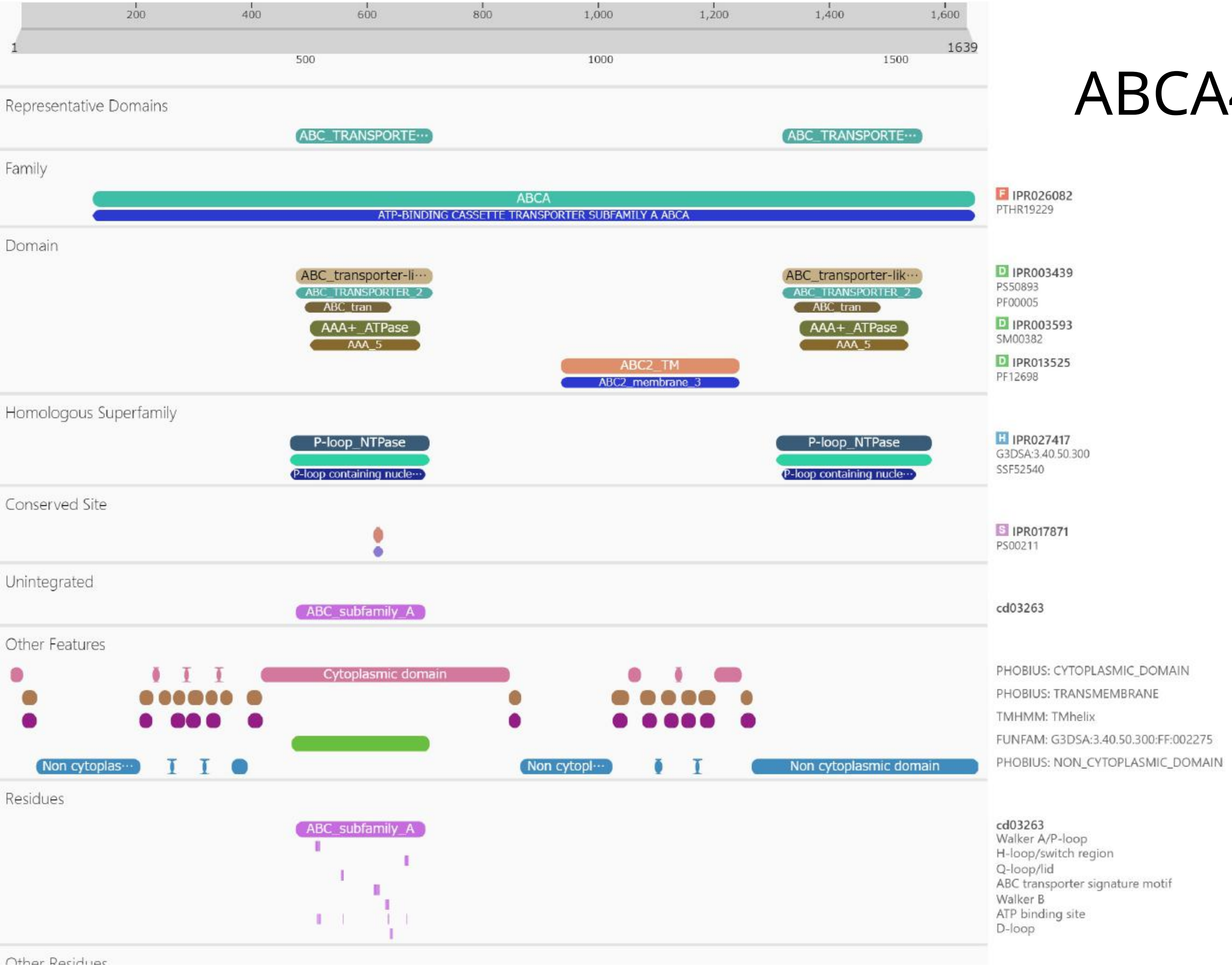

200
400
600
800
1,000
1,200
1,400
1,600
1
1639
500
1000
1500
Representative Domains
ABC_TRANSPORTE···
ABC_TRANSPORTE···
Family
ABCA
ATP-BINDING CASSETTE TRANSPORTER SUBFAMILY A ABCA
IPR026082
PTHR19229
Domain
ABC_transporter-li···
ABC_TRANSPORTER_2
ABC_tran
AAA+_ATPase
AAA_5
ABC_transporter-lik···
ABC2_TM
ABC2_membrane_3
IPR003439
PS50893
PF00005
IPR003593
SM00382
IPR013525
PF12698
Homologous Superfamily
P-loop_NTPase
P-loop containing nucle···
IPR027417
G3DSA:3.40.50.300
SSF52540
Conserved Site
IPR017871
PS00211
Unintegrated
ABC_subfamily_A
cd03263
Other Features
Cytoplasmic domain
Non cytoplas···
Non cytopl···
Non cytoplasmic domain
PHOBIUS: CYTOPLASMIC_DOMAIN
PHOBIUS: TRANSMEMBRANE
TMHMM: TMhelix
FUNFAM: G3DSA:3.40.50.300:FF:002275
PHOBIUS: NON_CYTOPLASMIC_DOMAIN
Residues
ABC_subfamily_A
cd03263
Walker A/P-loop
H-loop/switch region
Q-loop/lid
ABC transporter signature motif
Walker B
ATP binding site
D-loop

ABCA5

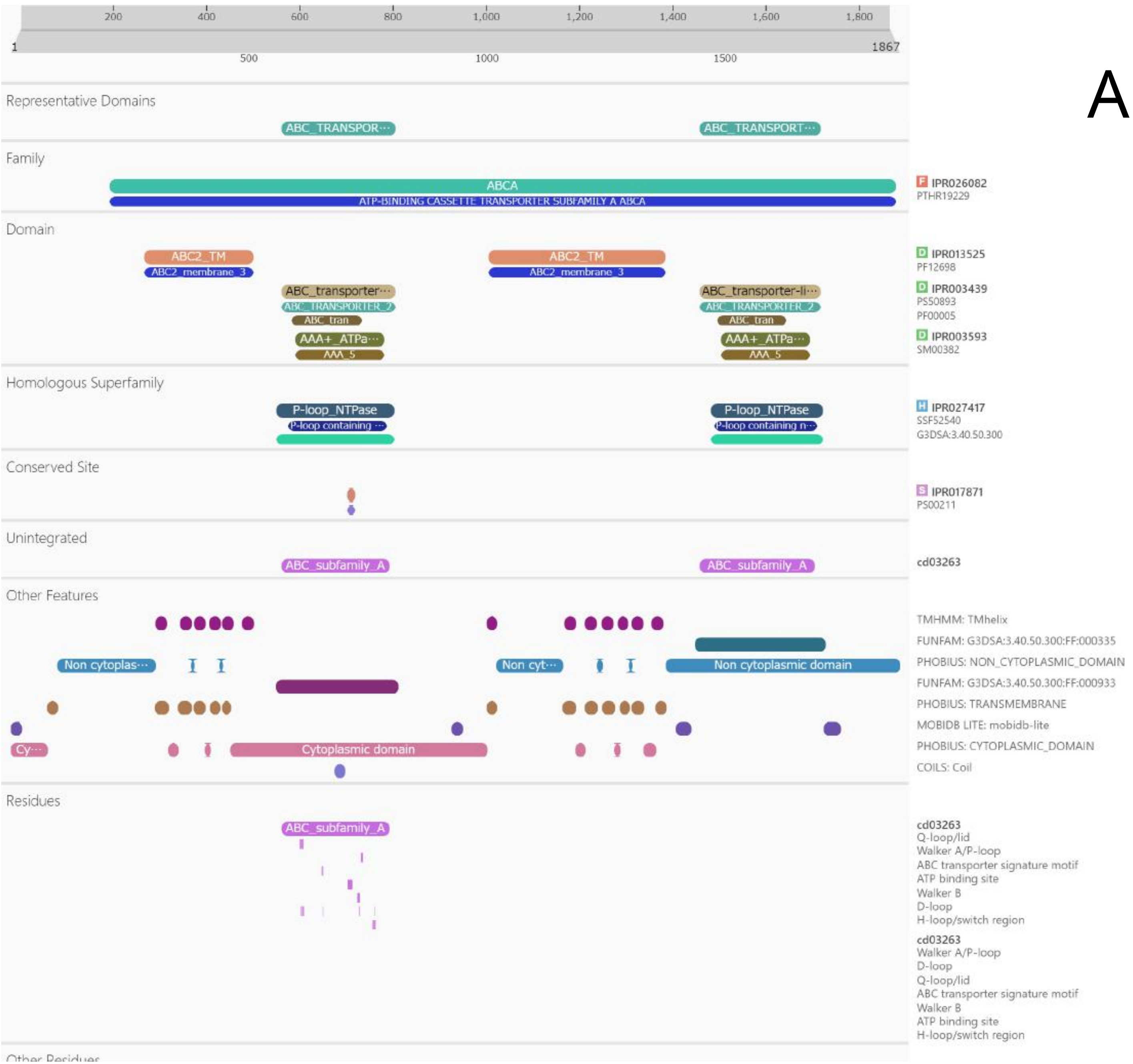

200
400
600
800
1,000
1,200
1,400
1,600
1,800
1
1867
500
1000
1500
Representative Domains
ABC_TRANSPOR···
ABC_TRANSPORT···
Family
ABCA
ATP-BINDING CASSETTE TRANSPORTER SUBFAMILY A ABCA
Domain
ABC2_TM
ABC2_membrane_3
ABC_transporter···
ABC_TRANSPORTER_2
ABC_tran
AAA+_ATPa···
AAA_5
ABC_transporter-li···
Homologous Superfamily
P-loop_NTPase
P-loop containing ···
P-loop containing n···
Conserved Site
Unintegrated
ABC_subfamily_A
Other Features
Non cytoplas···
Non cyt···
Non cytoplasmic domain
Cytoplasmic domain
Cy···
Residues
ABC_subfamily_A
Other Residues
F IPR026082
PTHR19229
D IPR013525
PF12698
D IPR003439
PS50893
PF00005
D IPR003593
SM00382
H IPR027417
SSF52540
G3DSA:3.40.50.300
S IPR017871
PS00211
cd03263
TMHMM: TMhelix
FUNFAM: G3DSA:3.40.50.300:FF:000335
PHOBIUS: NON_CYTOPLASMIC_DOMAIN
FUNFAM: G3DSA:3.40.50.300:FF:000933
PHOBIUS: TRANSMEMBRANE
MOBIDB LITE: mobidb-lite
PHOBIUS: CYTOPLASMIC_DOMAIN
COILS: Coil
cd03263
Q-loop/lid
Walker A/P-loop
ABC transporter signature motif
ATP binding site
Walker B
D-loop
H-loop/switch region
cd03263
Walker A/P-loop
D-loop
Q-loop/lid
ABC transporter signature motif
Walker B
ATP binding site
H-loop/switch region

ABCA1

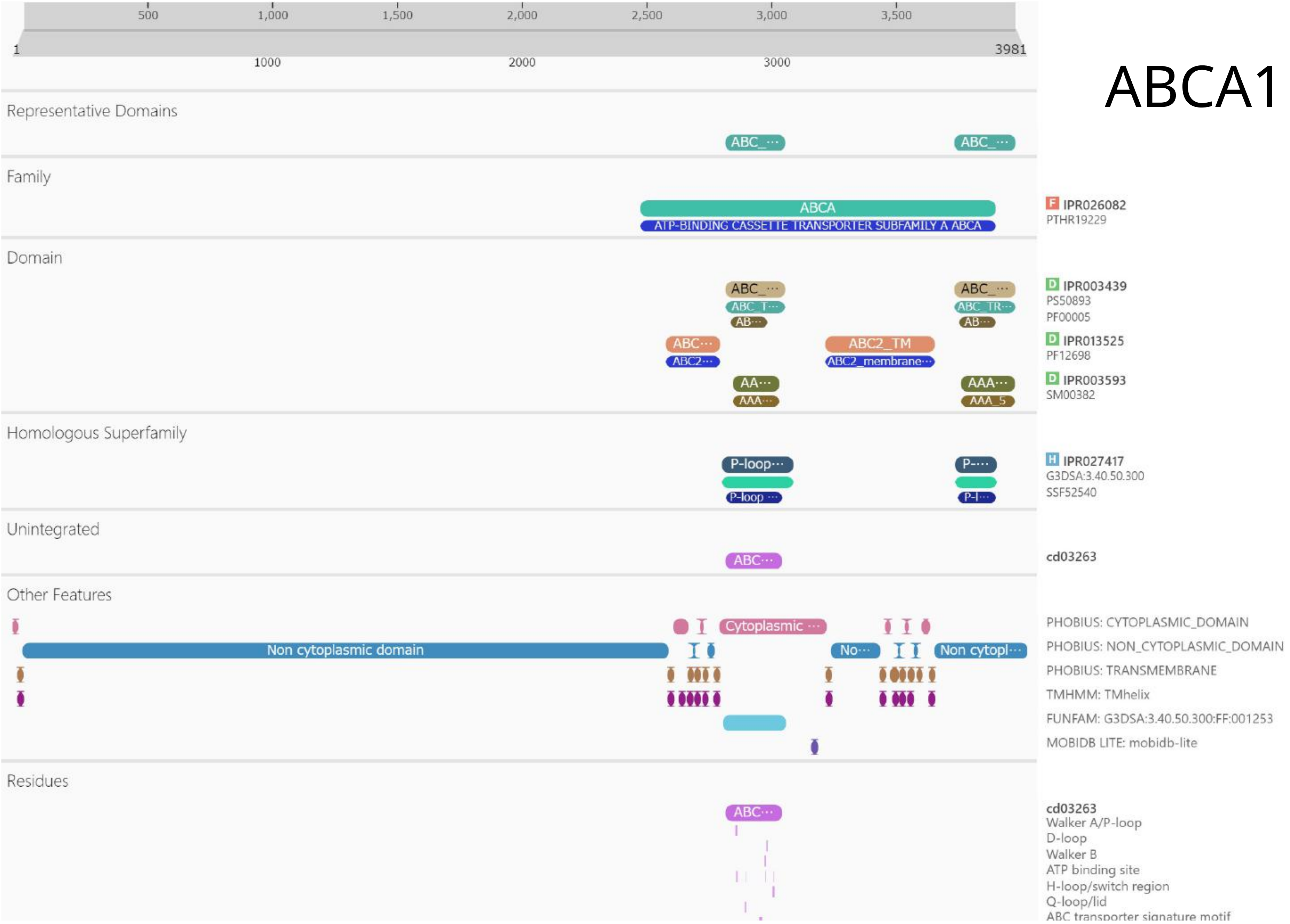

500
1,000
1,500
2,000
2,500
3,000
3,500
1
3981
1000
2000
3000
Representative Domains
ABC_···
ABC_···
Family
ABCA
ATP-BINDING CASSETTE TRANSPORTER SUBFAMILY A ABCA
IPR026082
PTHR19229
Domain
ABC_···
ABC2_TM
ABC2_membrane···
AAA_5
IPR003439
PS50893
PF00005
IPR013525
PF12698
IPR003593
SM00382
Homologous Superfamily
P-loop···
IPR027417
G3DSA:3.40.50.300
SSF52540
Unintegrated
ABC···
cd03263
Other Features
Cytoplasmic ···
Non cytoplasmic domain
Non cytopl···
PHOBIUS: CYTOPLASMIC_DOMAIN
PHOBIUS: NON_CYTOPLASMIC_DOMAIN
PHOBIUS: TRANSMEMBRANE
TMHMM: TMhelix
FUNFAM: G3DSA:3.40.50.300:FF:001253
MOBIDB LITE: mobidb-lite
Residues
ABC···
cd03263
Walker A/P-loop
D-loop
Walker B
ATP binding site
H-loop/switch region
Q-loop/lid
ABC transporter signature motif

ABCA12

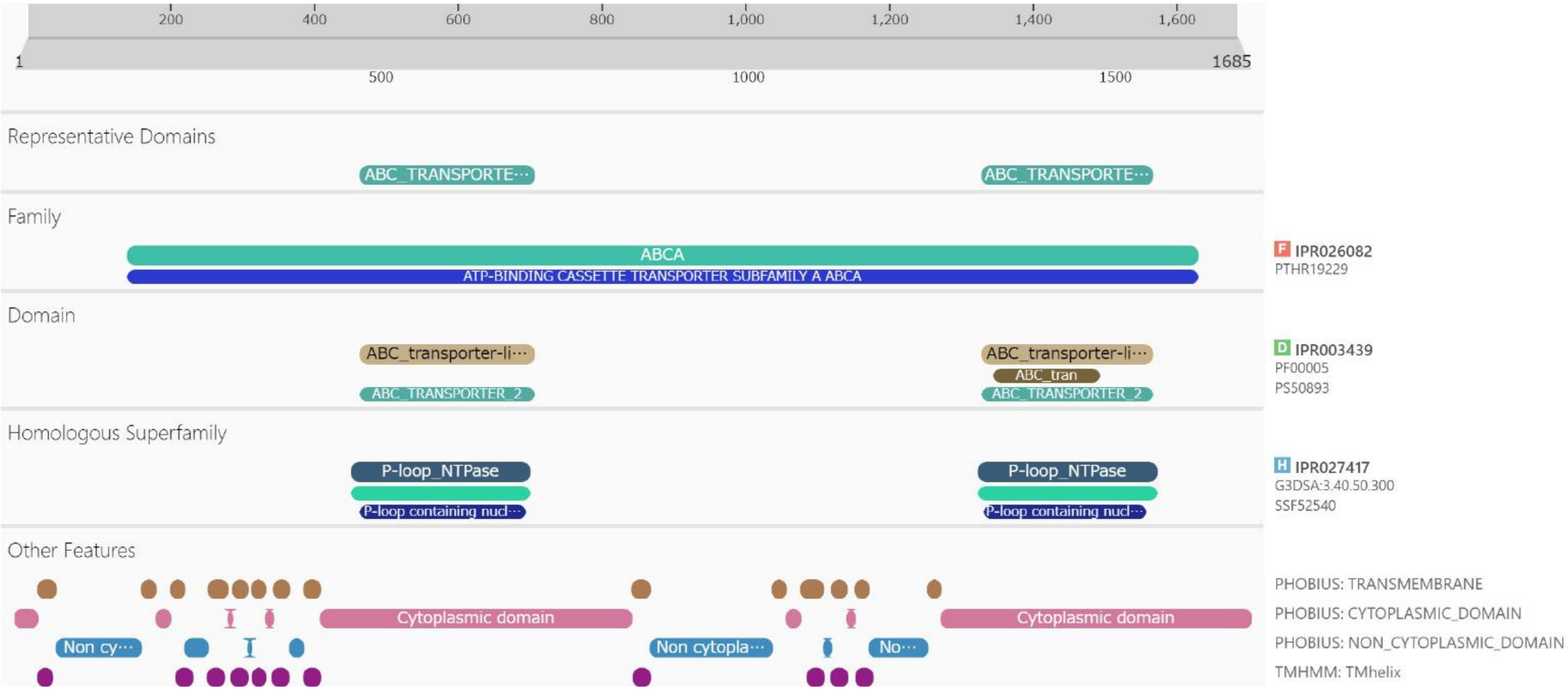

200
400
600
800
1,000
1,200
1,400
1,600
1
1685
500
1000
1500
Representative Domains
ABC_TRANSPORTE···
ABC_TRANSPORTE···
Family
ABCA
ATP-BINDING CASSETTE TRANSPORTER SUBFAMILY A ABCA
IPR026082
PTHR19229
Domain
ABC_transporter-li···
ABC_TRANSPORTER_2
ABC_transporter-li···
ABC_tran
ABC_TRANSPORTER_2
IPR003439
PF00005
PS50893
Homologous Superfamily
P-loop_NTPase
P-loop containing nucl···
P-loop_NTPase
P-loop containing nucl···
IPR027417
G3DSA:3.40.50.300
SSF52540
Other Features
Cytoplasmic domain
Cytoplasmic domain
Non cy···
Non cytopla···
No···
PHOBIUS: TRANSMEMBRANE
PHOBIUS: CYTOPLASMIC_DOMAIN
PHOBIUS: NON_CYTOPLASMIC_DOMAIN
TMHMM: TMhelix

ABCB1

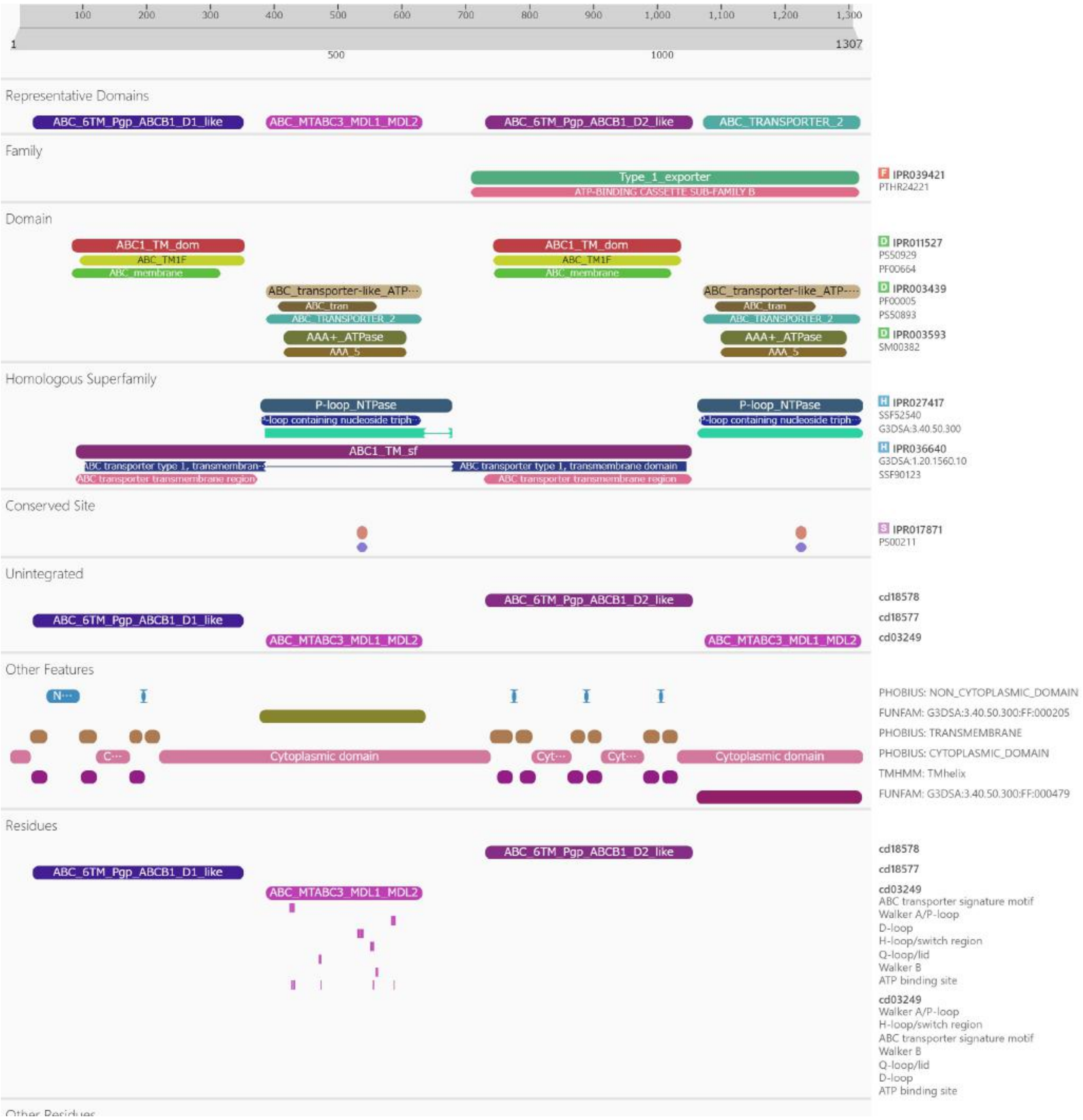

Representative Domains
ABC_6TM_Pgp_ABCB1_D1_like
ABC_MTABC3_MDL1_MDL2
ABC_6TM_Pgp_ABCB1_D2_like
ABC_TRANSPORTER_2
Family
Type_1_exporter
ATP-BINDING CASSETTE SUB-FAMILY B
IPR039421
PTHR24221
Domain
ABC1_TM_dom
ABC_TM1F
ABC_membrane
ABC_transporter-like_ATP···
ABC_tran
ABC_TRANSPORTER_2
AAA+_ATPase
AAA_5
IPR011527
PS50929
PF00664
IPR003439
PF00005
PS50893
IPR003593
SM00382
Homologous Superfamily
P-loop_NTPase
ABC1_TM_sf
ABC transporter type 1, transmembrane domain
ABC transporter transmembrane region
IPR027417
SSF52540
G3DSA:3.40.50.300
IPR036640
G3DSA:1.20.1560.10
SSF90123
Conserved Site
IPR017871
PS00211
Unintegrated
cd18578
cd18577
cd03249
Other Features
Cytoplasmic domain
PHOBIUS: NON_CYTOPLASMIC_DOMAIN
FUNFAM: G3DSA:3.40.50.300:FF:000205
PHOBIUS: TRANSMEMBRANE
PHOBIUS: CYTOPLASMIC_DOMAIN
TMHMM: TMhelix
FUNFAM: G3DSA:3.40.50.300:FF:000479
Residues
cd18578
cd18577
cd03249
ABC transporter signature motif
Walker A/P-loop
D-loop
H-loop/switch region
Q-loop/lid
Walker B
ATP binding site
cd03249
Walker A/P-loop
H-loop/switch region
ABC transporter signature motif
Walker B
Q-loop/lid
D-loop
ATP binding site

ABCB2

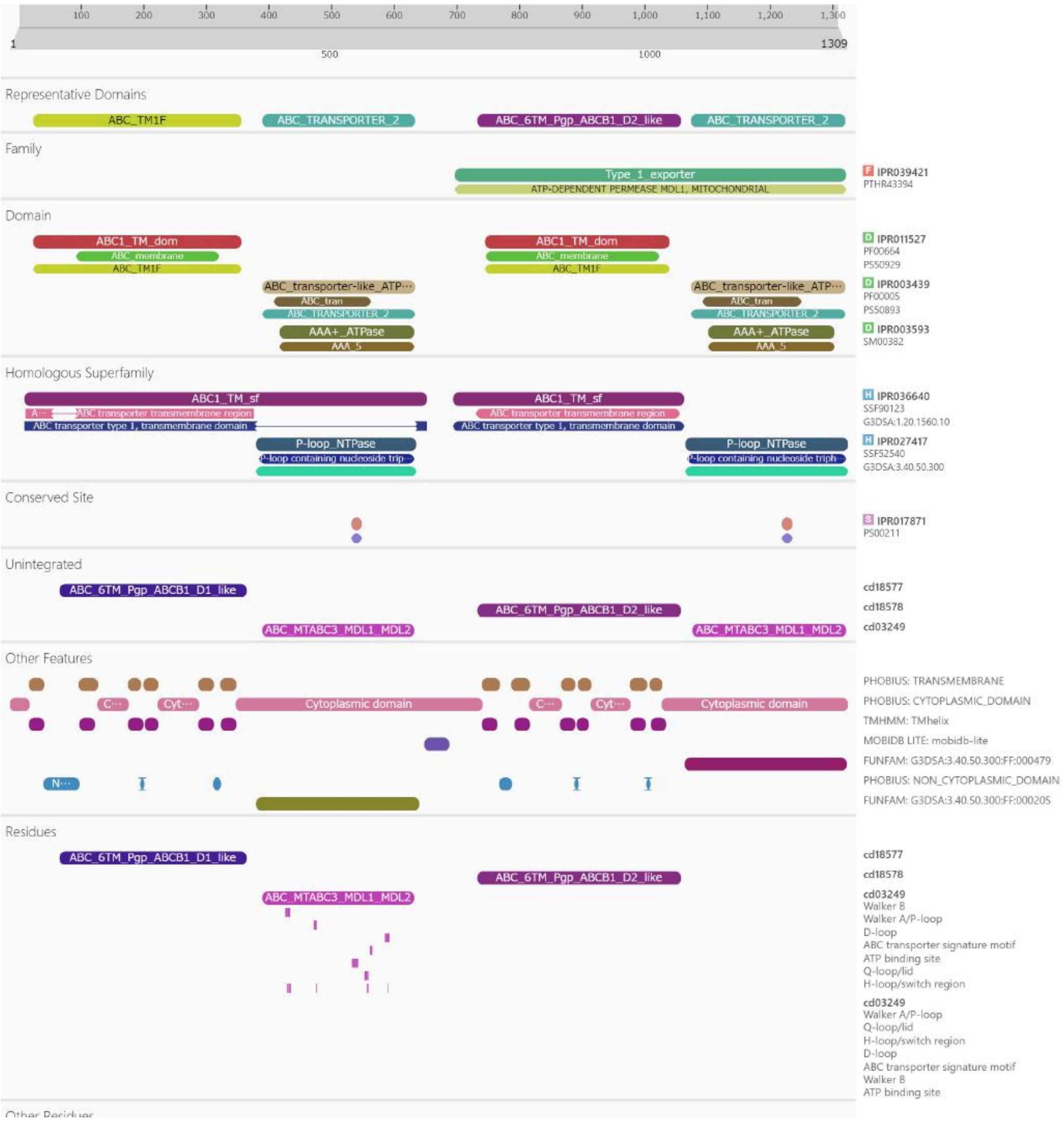

100
200
300
400
500
600
700
800
900
1,000
1,100
1,200
1,300
1
1309
500
1000
Representative Domains
ABC_TM1F
ABC_TRANSPORTER_2
ABC_6TM_Pgp_ABCB1_D2_like
ABC_TRANSPORTER_2
Family
Type_1_exporter
ATP-DEPENDENT PERMEASE MDL1, MITOCHONDRIAL
IPR039421
PTHR43394
Domain
ABC1_TM_dom
ABC_membrane
ABC_TM1F
ABC_transporter-like_ATP···
ABC_tran
ABC_TRANSPORTER_2
AAA+_ATPase
AAA_5
IPR011527
PF00664
PS50929
IPR003439
PF00005
PS50893
IPR003593
SM00382
Homologous Superfamily
ABC1_TM_sf
ABC transporter transmembrane region
ABC transporter type 1, transmembrane domain
P-loop_NTPase
P-loop containing nucleoside trip···
P-loop containing nucleoside triph···
IPR036640
SSF90123
G3DSA:1.20.1560.10
IPR027417
SSF52540
G3DSA:3.40.50.300
Conserved Site
IPR017871
PS00211
Unintegrated
ABC_6TM_Pgp_ABCB1_D1_like
ABC_6TM_Pgp_ABCB1_D2_like
ABC_MTABC3_MDL1_MDL2
cd18577
cd18578
cd03249
Other Features
C···
Cyt···
Cytoplasmic domain
N···
PHOBIUS: TRANSMEMBRANE
PHOBIUS: CYTOPLASMIC_DOMAIN
TMHMM: TMhelix
MOBIDB LITE: mobidb-lite
FUNFAM: G3DSA:3.40.50.300:FF:000479
PHOBIUS: NON_CYTOPLASMIC_DOMAIN
FUNFAM: G3DSA:3.40.50.300:FF:000205
Residues
ABC_6TM_Pgp_ABCB1_D1_like
ABC_6TM_Pgp_ABCB1_D2_like
ABC_MTABC3_MDL1_MDL2
cd18577
cd18578
cd03249
Walker B
Walker A/P-loop
D-loop
ABC transporter signature motif
ATP binding site
Q-loop/lid
H-loop/switch region
cd03249
Walker A/P-loop
Q-loop/lid
H-loop/switch region
D-loop
ABC transporter signature motif
Walker B
ATP binding site

ABCB3

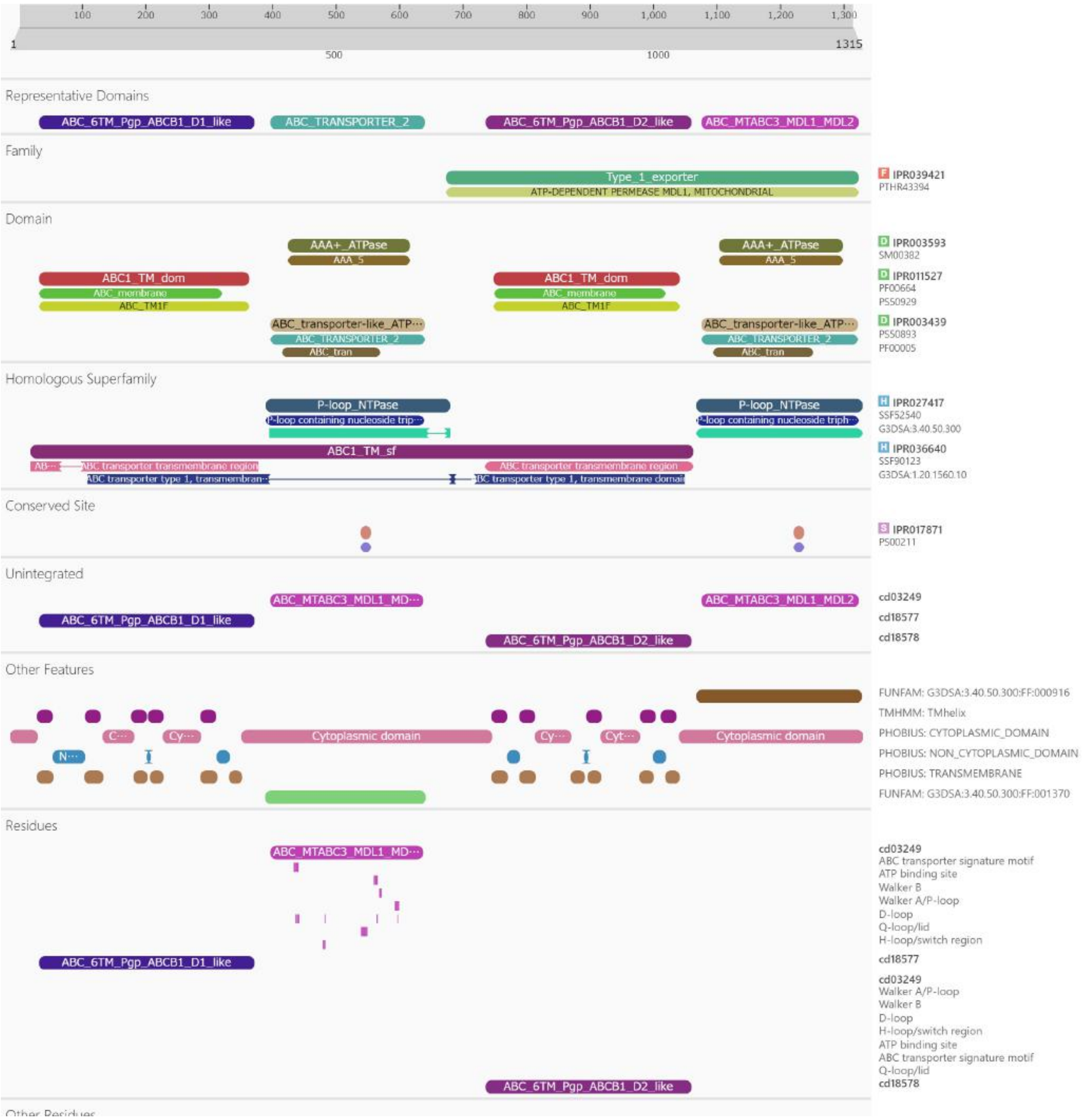

1315
500
1000
Representative Domains
ABC_6TM_Pgp_ABCB1_D1_like
ABC_TRANSPORTER_2
ABC_6TM_Pgp_ABCB1_D2_like
ABC_MTABC3_MDL1_MDL2
Family
Type_1_exporter
ATP-DEPENDENT PERMEASE MDL1, MITOCHONDRIAL
IPR039421
PTHR43394
Domain
AAA+_ATPase
AAA_5
ABC1_TM_dom
ABC_membrane
ABC_TM1F
ABC_transporter-like_ATP···
ABC_TRANSPORTER_2
ABC_tran
IPR003593
SM00382
IPR011527
PF00664
PS50929
IPR003439
PS50893
PF00005
Homologous Superfamily
P-loop_NTPase
P-loop containing nucleoside trip···
ABC1_TM_sf
ABC transporter transmembrane region
ABC transporter type 1, transmembrane domain
IPR027417
SSF52540
G3DSA:3.40.50.300
IPR036640
SSF90123
G3DSA:1.20.1560.10
Conserved Site
IPR017871
PS00211
Unintegrated
ABC_MTABC3_MDL1_MD···
cd03249
cd18577
cd18578
Other Features
Cytoplasmic domain
FUNFAM: G3DSA:3.40.50.300:FF:000916
TMHMM: TMhelix
PHOBIUS: CYTOPLASMIC_DOMAIN
PHOBIUS: NON_CYTOPLASMIC_DOMAIN
PHOBIUS: TRANSMEMBRANE
FUNFAM: G3DSA:3.40.50.300:FF:001370
Residues
cd03249
ABC transporter signature motif
ATP binding site
Walker B
Walker A/P-loop
D-loop
Q-loop/lid
H-loop/switch region
cd18577
cd03249
Walker A/P-loop
Walker B
D-loop
H-loop/switch region
ATP binding site
ABC transporter signature motif
Q-loop/lid
cd18578

ABCB4

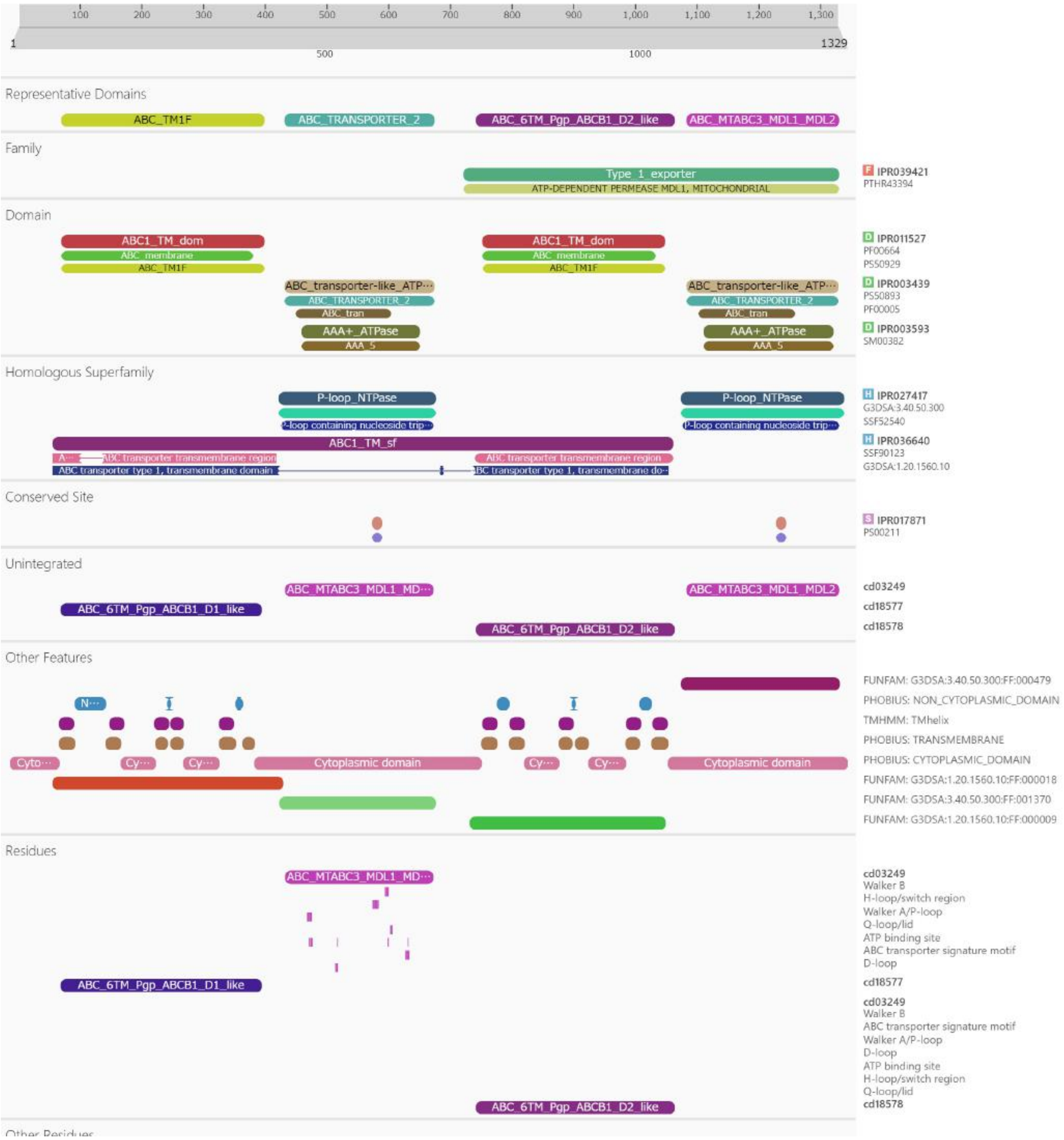

100
200
300
400
500
600
700
800
900
1,000
1,100
1,200
1,300
1
1329
500
1000
Representative Domains
ABC_TM1F
ABC_TRANSPORTER_2
ABC_6TM_Pgp_ABCB1_D2_like
ABC_MTABC3_MDL1_MDL2
Family
Type_1_exporter
ATP-DEPENDENT PERMEASE MDL1, MITOCHONDRIAL
IPR039421
PTHR43394
Domain
ABC1_TM_dom
ABC_membrane
ABC_TM1F
ABC_transporter-like_ATP···
ABC_TRANSPORTER_2
ABC_tran
AAA+_ATPase
AAA_5
IPR011527
PF00664
PS50929
IPR003439
PS50893
PF00005
IPR003593
SM00382
Homologous Superfamily
P-loop_NTPase
P-loop containing nucleoside trip···
ABC1_TM_sf
ABC transporter transmembrane region
ABC transporter type 1, transmembrane domain
IPR027417
G3DSA:3.40.50.300
SSF52540
IPR036640
SSF90123
G3DSA:1.20.1560.10
Conserved Site
IPR017871
PS00211
Unintegrated
ABC_MTABC3_MDL1_MD···
ABC_MTABC3_MDL1_MDL2
ABC_6TM_Pgp_ABCB1_D1_like
ABC_6TM_Pgp_ABCB1_D2_like
cd03249
cd18577
cd18578
Other Features
Cytoplasmic domain
FUNFAM: G3DSA:3.40.50.300:FF:000479
PHOBIUS: NON_CYTOPLASMIC_DOMAIN
TMHMM: TMhelix
PHOBIUS: TRANSMEMBRANE
PHOBIUS: CYTOPLASMIC_DOMAIN
FUNFAM: G3DSA:1.20.1560.10:FF:000018
FUNFAM: G3DSA:3.40.50.300:FF:001370
FUNFAM: G3DSA:1.20.1560.10:FF:000009
Residues
ABC_MTABC3_MDL1_MD···
ABC_6TM_Pgp_ABCB1_D1_like
ABC_6TM_Pgp_ABCB1_D2_like
cd03249
Walker B
H-loop/switch region
Walker A/P-loop
Q-loop/lid
ATP binding site
ABC transporter signature motif
D-loop
cd18577
cd03249
Walker B
ABC transporter signature motif
Walker A/P-loop
D-loop
ATP binding site
H-loop/switch region
Q-loop/lid
cd18578

ABCB5

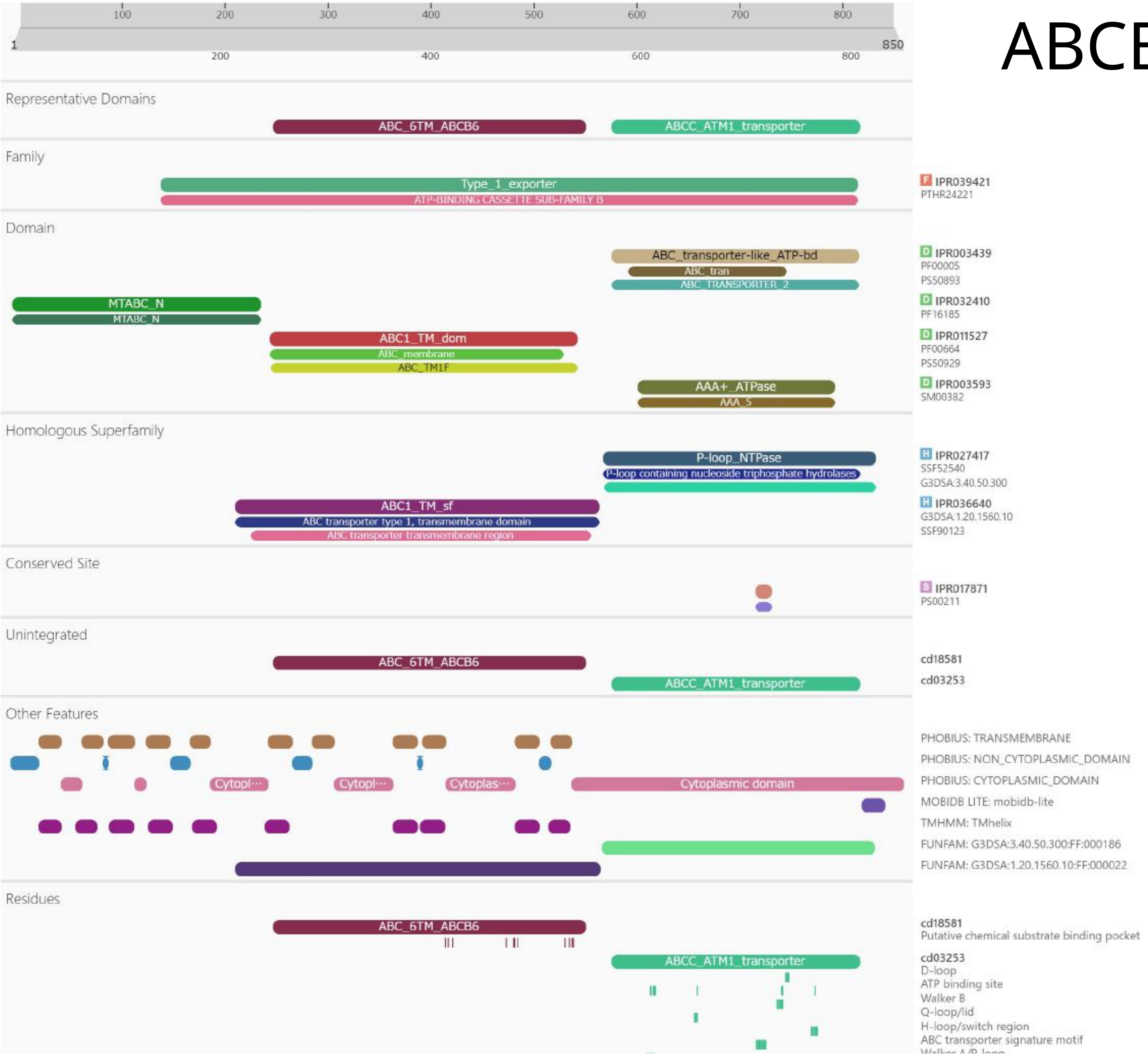

100
200
300
400
500
600
700
800
1
850
Representative Domains
ABC_6TM_ABCB6
ABCC_ATM1_transporter
Family
Type_1_exporter
ATP-BINDING CASSETTE SUB-FAMILY B
IPR039421
PTHR24221
Domain
ABC_transporter-like_ATP-bd
ABC_tran
ABC_TRANSPORTER_2
MTABC_N
MTABC_N
ABC1_TM_dom
ABC_membrane
ABC_TM1F
AAA+_ATPase
AAA_5
IPR003439
PF00005
PS50893
IPR032410
PF16185
IPR011527
PF00664
PS50929
IPR003593
SM00382
Homologous Superfamily
P-loop_NTPase
P-loop containing nucleoside triphosphate hydrolases
ABC1_TM_sf
ABC transporter type 1, transmembrane domain
ABC transporter transmembrane region
IPR027417
SSF52540
G3DSA:3.40.50.300
IPR036640
G3DSA:1.20.1560.10
SSF90123
Conserved Site
IPR017871
PS00211
Unintegrated
ABC_6TM_ABCB6
ABCC_ATM1_transporter
cd18581
cd03253
Other Features
Cytopl···
Cytopl···
Cytoplas···
Cytoplasmic domain
PHOBIUS: TRANSMEMBRANE
PHOBIUS: NON_CYTOPLASMIC_DOMAIN
PHOBIUS: CYTOPLASMIC_DOMAIN
MOBIDB LITE: mobidb-lite
TMHMM: TMhelix
FUNFAM: G3DSA:3.40.50.300:FF:000186
FUNFAM: G3DSA:1.20.1560.10:FF:000022
Residues
ABC_6TM_ABCB6
ABCC_ATM1_transporter
cd18581
Putative chemical substrate binding pocket
cd03253
D-loop
ATP binding site
Walker B
Q-loop/lid
H-loop/switch region
ABC transporter signature motif

ABCB6

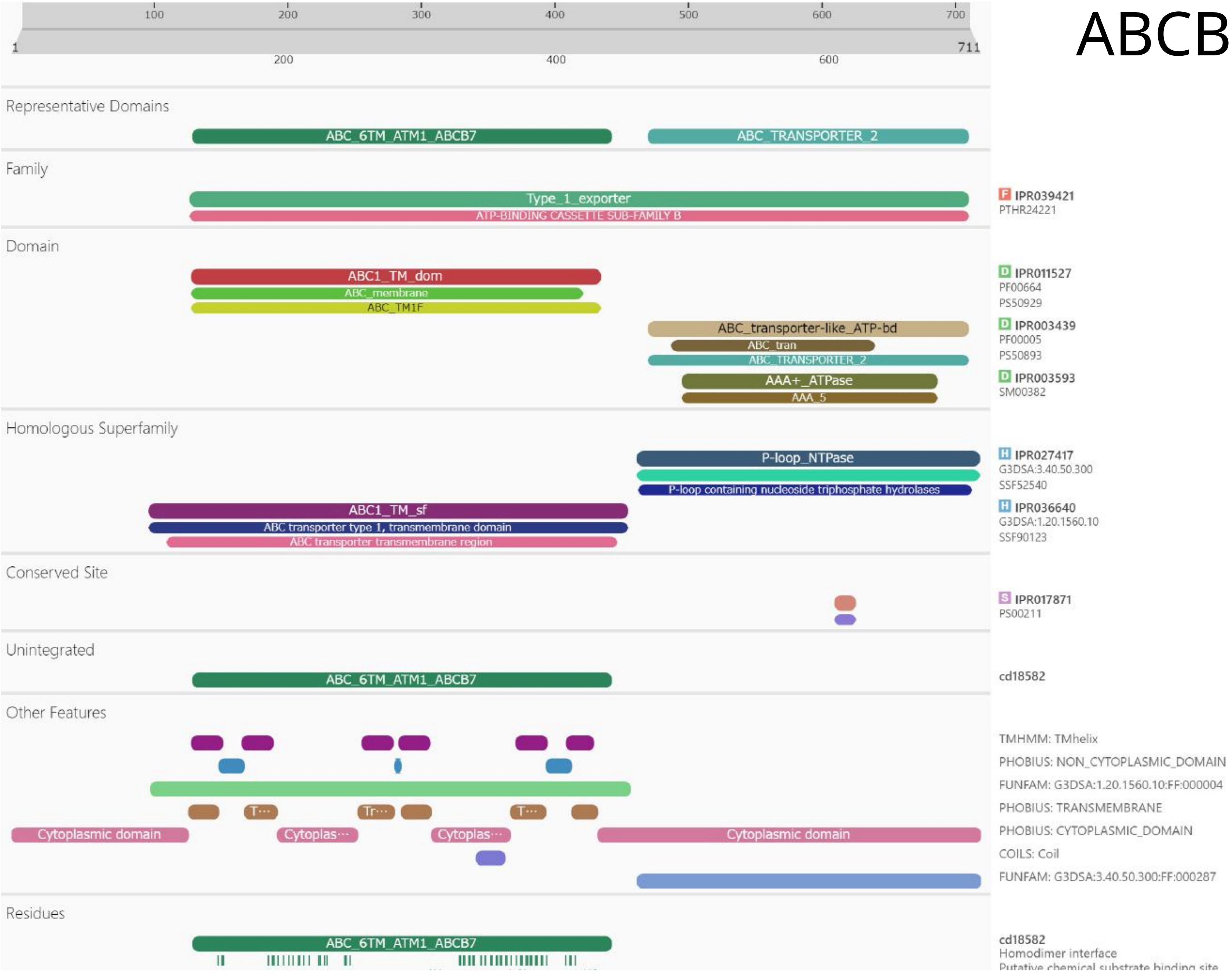

100
200
300
400
500
600
700
1
711
200
400
600
Representative Domains
ABC_6TM_ATM1_ABCB7
ABC_TRANSPORTER_2
Family
Type_1_exporter
ATP-BINDING CASSETTE SUB-FAMILY B
IPR039421
PTHR24221
Domain
ABC1_TM_dom
ABC_membrane
ABC_TM1F
IPR011527
PF00664
PS50929
ABC_transporter-like_ATP-bd
ABC_tran
ABC_TRANSPORTER_2
IPR003439
PF00005
PS50893
AAA+_ATPase
AAA_5
IPR003593
SM00382
Homologous Superfamily
P-loop_NTPase
P-loop containing nucleoside triphosphate hydrolases
IPR027417
G3DSA:3.40.50.300
SSF52540
ABC1_TM_sf
ABC transporter type 1, transmembrane domain
ABC transporter transmembrane region
IPR036640
G3DSA:1.20.1560.10
SSF90123
Conserved Site
IPR017871
PS00211
Unintegrated
ABC_6TM_ATM1_ABCB7
cd18582
Other Features
TMHMM: TMhelix
PHOBIUS: NON_CYTOPLASMIC_DOMAIN
FUNFAM: G3DSA:1.20.1560.10:FF:000004
PHOBIUS: TRANSMEMBRANE
PHOBIUS: CYTOPLASMIC_DOMAIN
COILS: Coil
FUNFAM: G3DSA:3.40.50.300:FF:000287
Cytoplasmic domain
Cytoplas···
Cytoplas···
Cytoplasmic domain
Residues
ABC_6TM_ATM1_ABCB7
cd18582
Homodimer interface

ABCB7

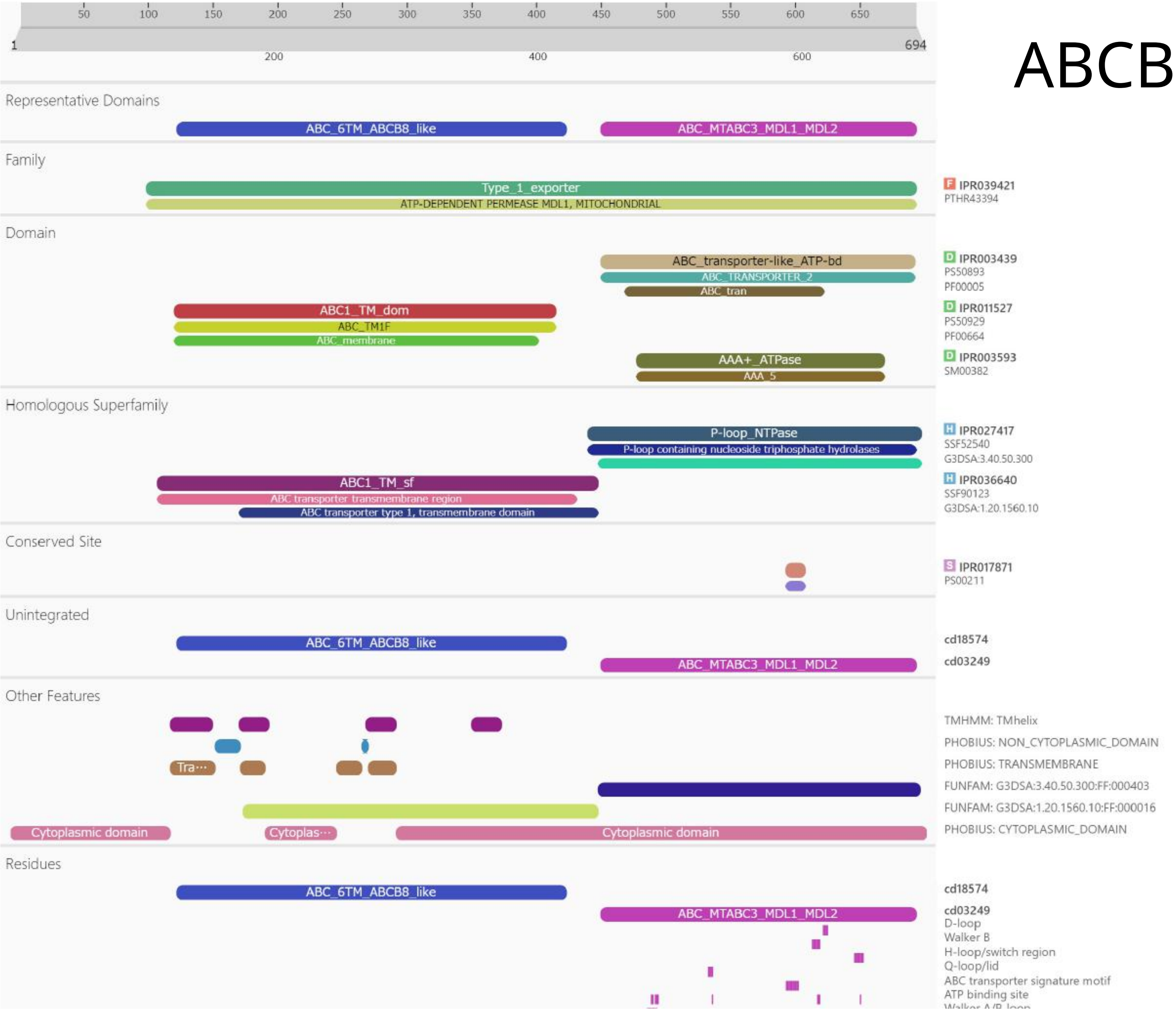

50 100 150 200 250 300 350 400 450 500 550 600 650
1
694
200 400 600
Representative Domains
ABC_6TM_ABCB8_like
ABC_MTABC3_MDL1_MDL2
Family
Type_1_exporter
ATP-DEPENDENT PERMEASE MDL1, MITOCHONDRIAL
IPR039421
PTHR43394
Domain
ABC_transporter-like_ATP-bd
ABC_TRANSPORTER_2
ABC_tran
ABC1_TM_dom
ABC_TM1F
ABC_membrane
AAA+_ATPase
AAA_5
IPR003439
PS50893
PF00005
IPR011527
PS50929
PF00664
IPR003593
SM00382
Homologous Superfamily
P-loop_NTPase
P-loop containing nucleoside triphosphate hydrolases
ABC1_TM_sf
ABC transporter transmembrane region
ABC transporter type 1, transmembrane domain
IPR027417
SSF52540
G3DSA:3.40.50.300
IPR036640
SSF90123
G3DSA:1.20.1560.10
Conserved Site
IPR017871
PS00211
Unintegrated
ABC_6TM_ABCB8_like
ABC_MTABC3_MDL1_MDL2
cd18574
cd03249
Other Features
Tra···
Cytoplasmic domain
Cytoplas···
Cytoplasmic domain
TMHMM: TMhelix
PHOBIUS: NON_CYTOPLASMIC_DOMAIN
PHOBIUS: TRANSMEMBRANE
FUNFAM: G3DSA:3.40.50.300:FF:000403
FUNFAM: G3DSA:1.20.1560.10:FF:000016
PHOBIUS: CYTOPLASMIC_DOMAIN
Residues
ABC_6TM_ABCB8_like
ABC_MTABC3_MDL1_MDL2
cd18574
cd03249
D-loop
Walker B
H-loop/switch region
Q-loop/lid
ABC transporter signature motif
ATP binding site

ABCB8

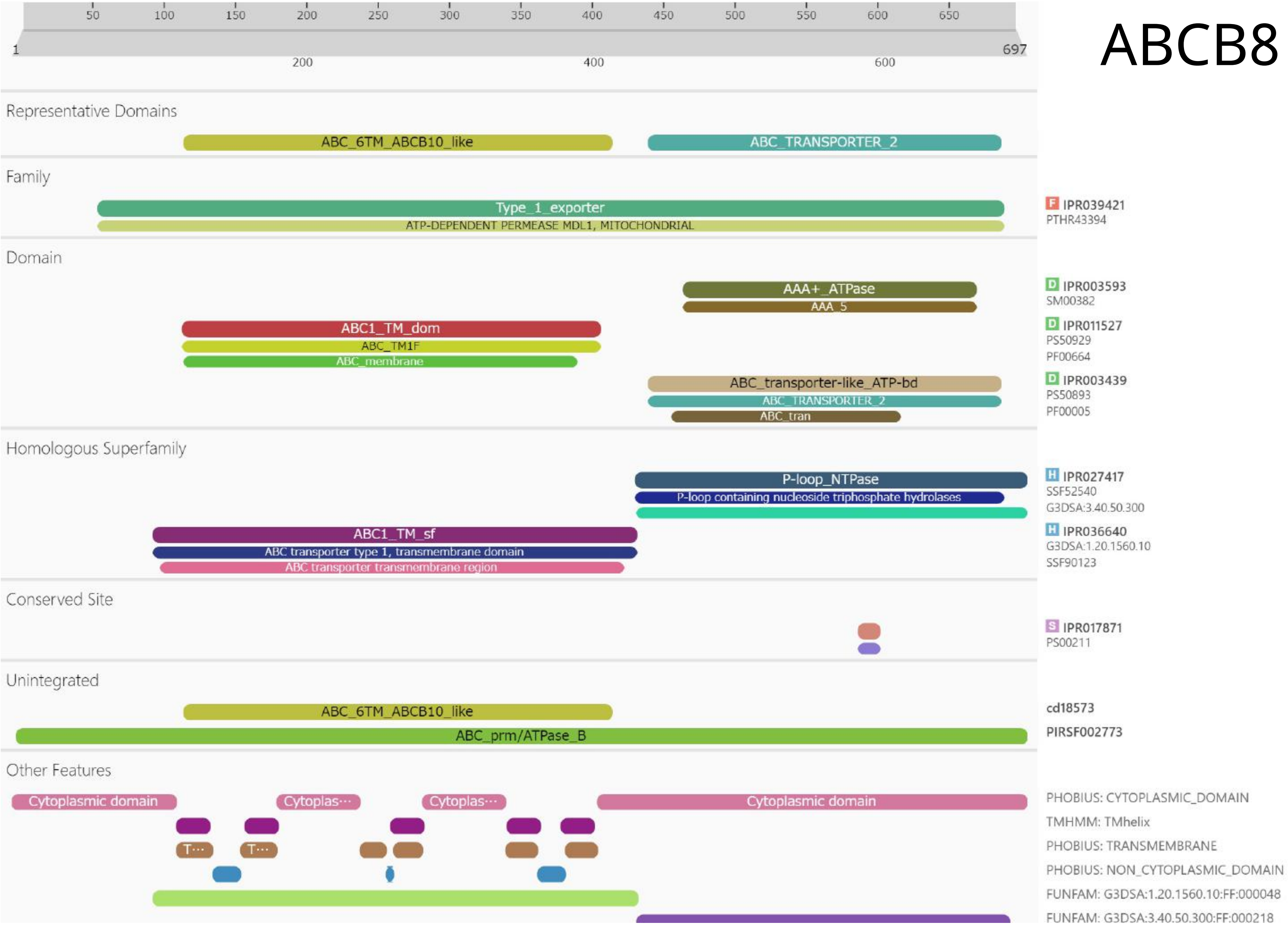

50
100
150
200
250
300
350
400
450
500
550
600
650
1
697
200
400
600
Representative Domains
ABC_6TM_ABCB10_like
ABC_TRANSPORTER_2
Family
Type_1_exporter
ATP-DEPENDENT PERMEASE MDL1, MITOCHONDRIAL
F IPR039421
PTHR43394
Domain
AAA+_ATPase
AAA_5
D IPR003593
SM00382
ABC1_TM_dom
ABC_TM1F
ABC_membrane
D IPR011527
PS50929
PF00664
ABC_transporter-like_ATP-bd
ABC_TRANSPORTER_2
ABC_tran
D IPR003439
PS50893
PF00005
Homologous Superfamily
P-loop_NTPase
P-loop containing nucleoside triphosphate hydrolases
H IPR027417
SSF52540
G3DSA:3.40.50.300
ABC1_TM_sf
ABC transporter type 1, transmembrane domain
ABC transporter transmembrane region
H IPR036640
G3DSA:1.20.1560.10
SSF90123
Conserved Site
S IPR017871
PS00211
Unintegrated
ABC_6TM_ABCB10_like
cd18573
ABC_prm/ATPase_B
PIRSF002773
Other Features
Cytoplasmic domain
Cytoplas···
Cytoplas···
Cytoplasmic domain
T···
T···
PHOBIUS: CYTOPLASMIC_DOMAIN
TMHMM: TMhelix
PHOBIUS: TRANSMEMBRANE
PHOBIUS: NON_CYTOPLASMIC_DOMAIN
FUNFAM: G3DSA:1.20.1560.10:FF:000048
FUNFAM: G3DSA:3.40.50.300:FF:000218

ABCB9

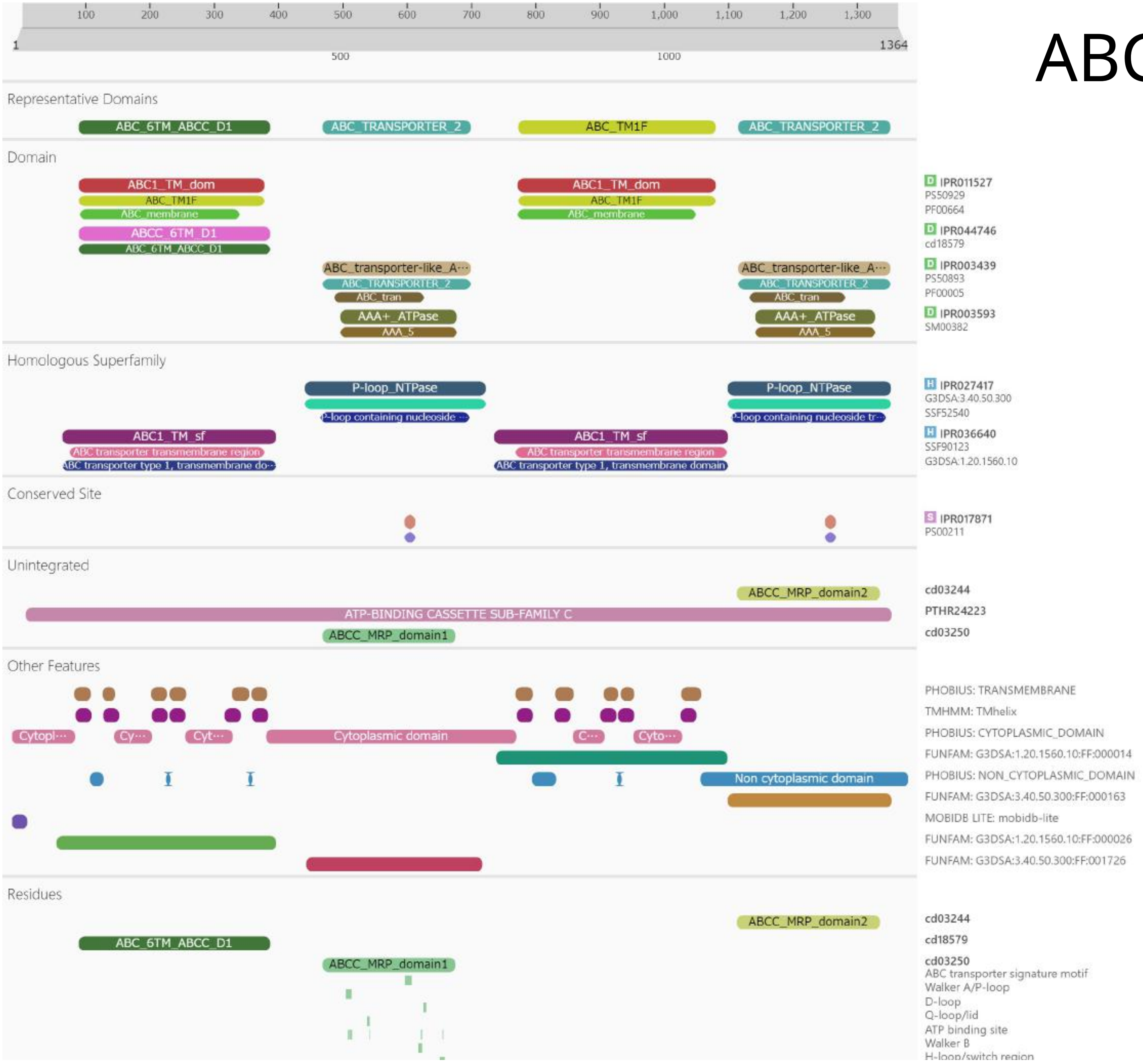

1
1364
500
1000
Representative Domains
ABC_6TM_ABCC_D1
ABC_TRANSPORTER_2
ABC_TM1F
ABC_TRANSPORTER_2
Domain
ABC1_TM_dom
ABC_TM1F
ABC_membrane
ABCC_6TM_D1
ABC_6TM_ABCC_D1
ABC_transporter-like_A···
ABC_TRANSPORTER_2
ABC_tran
AAA+_ATPase
AAA_5
IPR011527
PS50929
PF00664
IPR044746
cd18579
IPR003439
PS50893
PF00005
IPR003593
SM00382
Homologous Superfamily
P-loop_NTPase
ABC1_TM_sf
ABC transporter transmembrane region
ABC transporter type 1, transmembrane domain
IPR027417
G3DSA:3.40.50.300
SSF52540
IPR036640
SSF90123
G3DSA:1.20.1560.10
Conserved Site
IPR017871
PS00211
Unintegrated
ABCC_MRP_domain2
ATP-BINDING CASSETTE SUB-FAMILY C
ABCC_MRP_domain1
cd03244
PTHR24223
cd03250
Other Features
Cytoplasmic domain
Non cytoplasmic domain
PHOBIUS: TRANSMEMBRANE
TMHMM: TMhelix
PHOBIUS: CYTOPLASMIC_DOMAIN
FUNFAM: G3DSA:1.20.1560.10:FF:000014
PHOBIUS: NON_CYTOPLASMIC_DOMAIN
FUNFAM: G3DSA:3.40.50.300:FF:000163
MOBIDB LITE: mobidb-lite
FUNFAM: G3DSA:1.20.1560.10:FF:000026
FUNFAM: G3DSA:3.40.50.300:FF:001726
Residues
ABCC_MRP_domain2
ABC_6TM_ABCC_D1
ABCC_MRP_domain1
cd03244
cd18579
cd03250
ABC transporter signature motif
Walker A/P-loop
D-loop
Q-loop/lid
ATP binding site
Walker B
H-loop/switch region

ABCC3

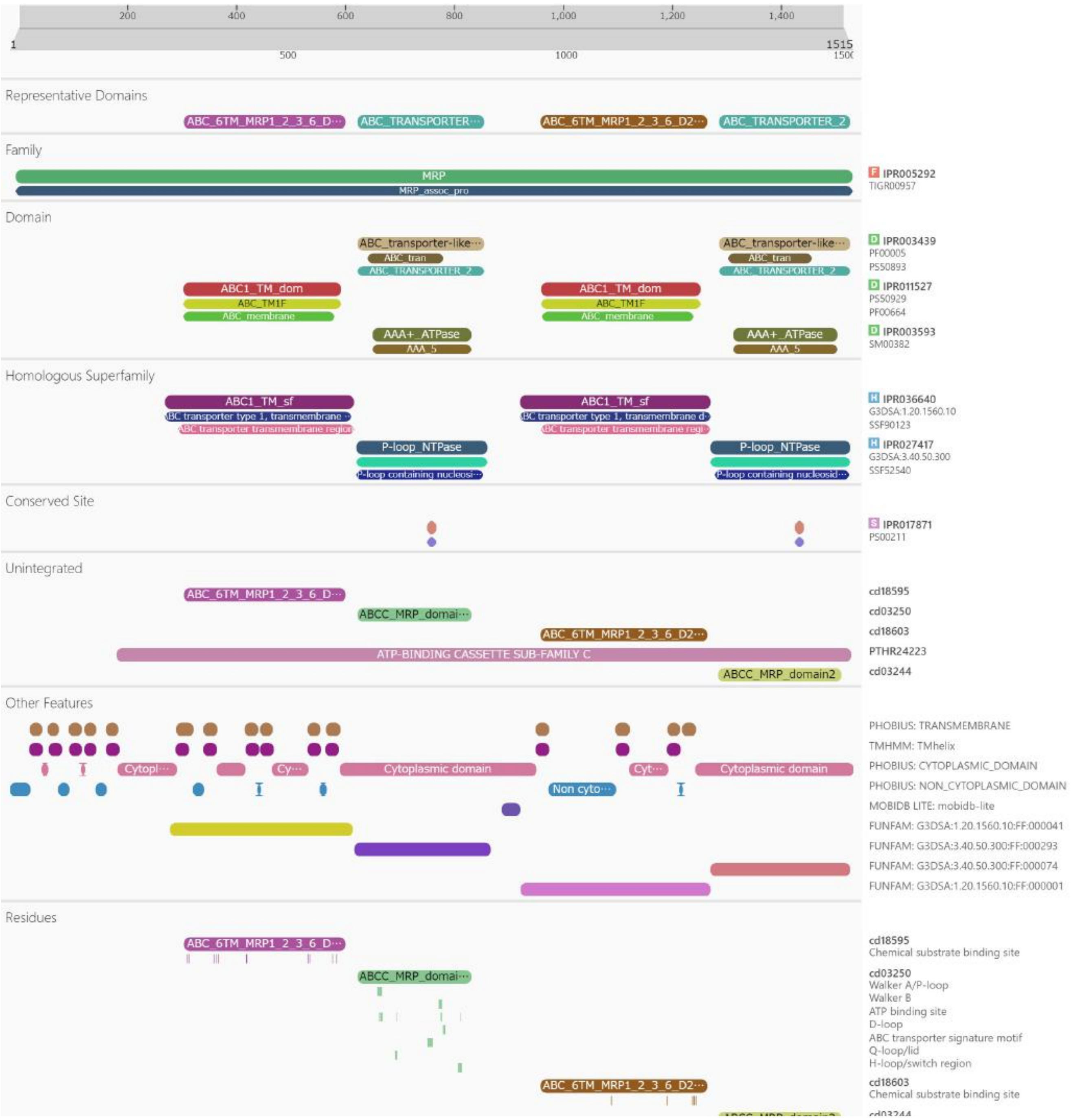

Representative Domains
ABC_6TM_MRP1_2_3_6_D···
ABC_TRANSPORTER···
ABC_6TM_MRP1_2_3_6_D2···
ABC_TRANSPORTER_2
Family
MRP
MRP_assoc_pro
IPR005292
TIGR00957
Domain
ABC_transporter-like···
ABC_tran
ABC_TRANSPORTER_2
ABC1_TM_dom
ABC_TM1F
ABC_membrane
AAA+_ATPase
AAA_5
IPR003439
PF00005
PS50893
IPR011527
PS50929
PF00664
IPR003593
SM00382
Homologous Superfamily
ABC1_TM_sf
P-loop_NTPase
IPR036640
G3DSA:1.20.1560.10
SSF90123
IPR027417
G3DSA:3.40.50.300
SSF52540
Conserved Site
IPR017871
PS00211
Unintegrated
ABCC_MRP_domai···
ATP-BINDING CASSETTE SUB-FAMILY C
ABCC_MRP_domain2
cd18595
cd03250
cd18603
PTHR24223
cd03244
Other Features
Cytoplasmic domain
Non cyto···
PHOBIUS: TRANSMEMBRANE
TMHMM: TMhelix
PHOBIUS: CYTOPLASMIC_DOMAIN
PHOBIUS: NON_CYTOPLASMIC_DOMAIN
MOBIDB LITE: mobidb-lite
FUNFAM: G3DSA:1.20.1560.10:FF:000041
FUNFAM: G3DSA:3.40.50.300:FF:000293
FUNFAM: G3DSA:3.40.50.300:FF:000074
FUNFAM: G3DSA:1.20.1560.10:FF:000001
Residues
cd18595
Chemical substrate binding site
cd03250
Walker A/P-loop
Walker B
ATP binding site
D-loop
ABC transporter signature motif
Q-loop/lid
H-loop/switch region
cd18603
Chemical substrate binding site

ABCC1

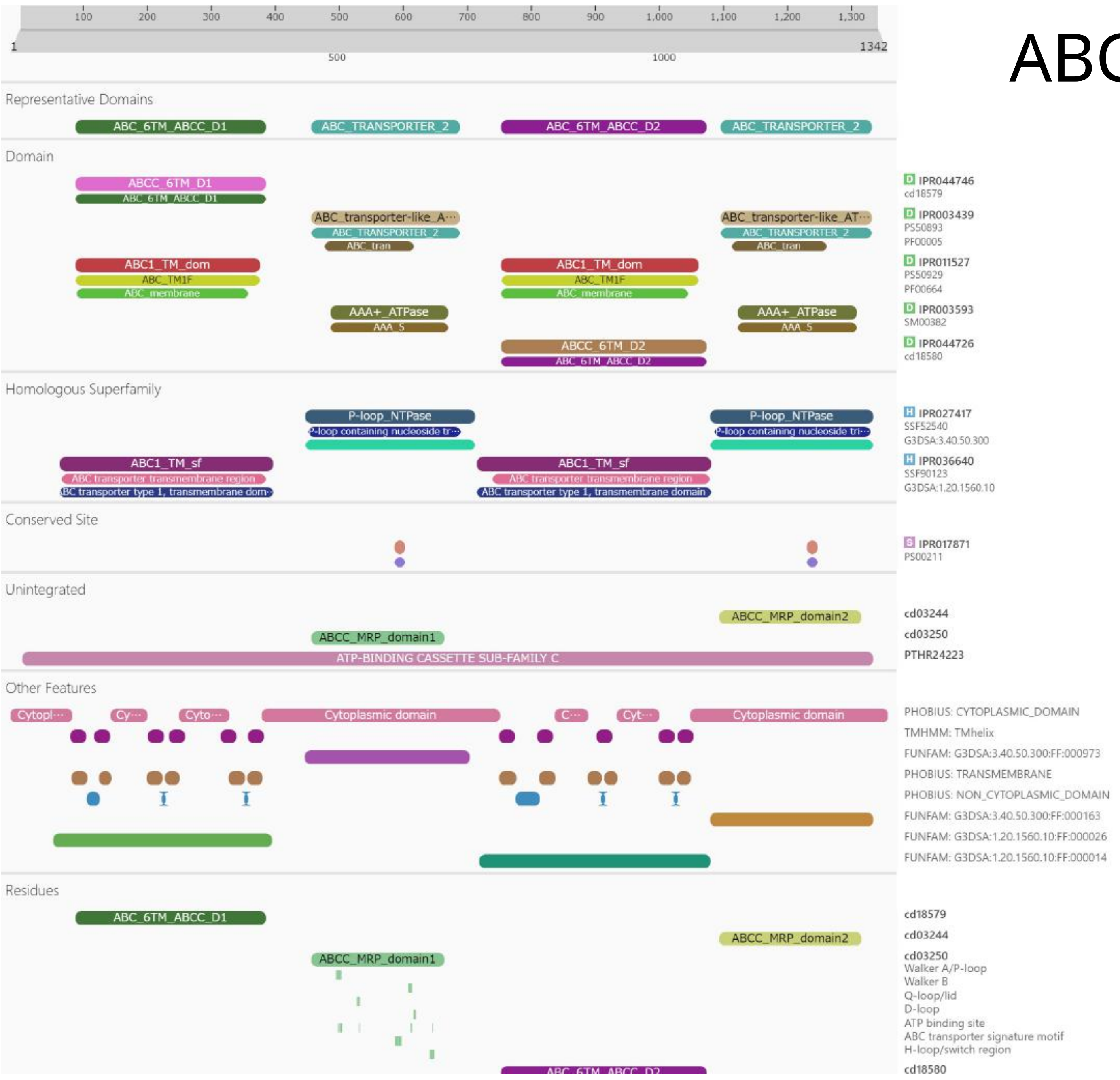

Representative Domains
ABC_6TM_ABCC_D1
ABC_TRANSPORTER_2
ABC_6TM_ABCC_D2
ABC_TRANSPORTER_2
Domain
ABCC_6TM_D1
ABC_transporter-like_A···
ABC1_TM_dom
ABC_TM1F
ABC_membrane
AAA+_ATPase
AAA_5
ABCC_6TM_D2
ABC_tran
IPR044746
cd18579
IPR003439
PS50893
PF00005
IPR011527
PS50929
PF00664
IPR003593
SM00382
IPR044726
cd18580
Homologous Superfamily
P-loop_NTPase
P-loop containing nucleoside tri···
ABC1_TM_sf
ABC transporter transmembrane region
ABC transporter type 1, transmembrane domain
IPR027417
SSF52540
G3DSA:3.40.50.300
IPR036640
SSF90123
G3DSA:1.20.1560.10
Conserved Site
IPR017871
PS00211
Unintegrated
ABCC_MRP_domain2
ABCC_MRP_domain1
ATP-BINDING CASSETTE SUB-FAMILY C
cd03244
cd03250
PTHR24223
Other Features
Cytoplasmic domain
PHOBIUS: CYTOPLASMIC_DOMAIN
TMHMM: TMhelix
FUNFAM: G3DSA:3.40.50.300:FF:000973
PHOBIUS: TRANSMEMBRANE
PHOBIUS: NON_CYTOPLASMIC_DOMAIN
FUNFAM: G3DSA:3.40.50.300:FF:000163
FUNFAM: G3DSA:1.20.1560.10:FF:000026
FUNFAM: G3DSA:1.20.1560.10:FF:000014
Residues
cd18579
cd03244
cd03250
Walker A/P-loop
Walker B
Q-loop/lid
D-loop
ATP binding site
ABC transporter signature motif
H-loop/switch region
cd18580

ABCC2

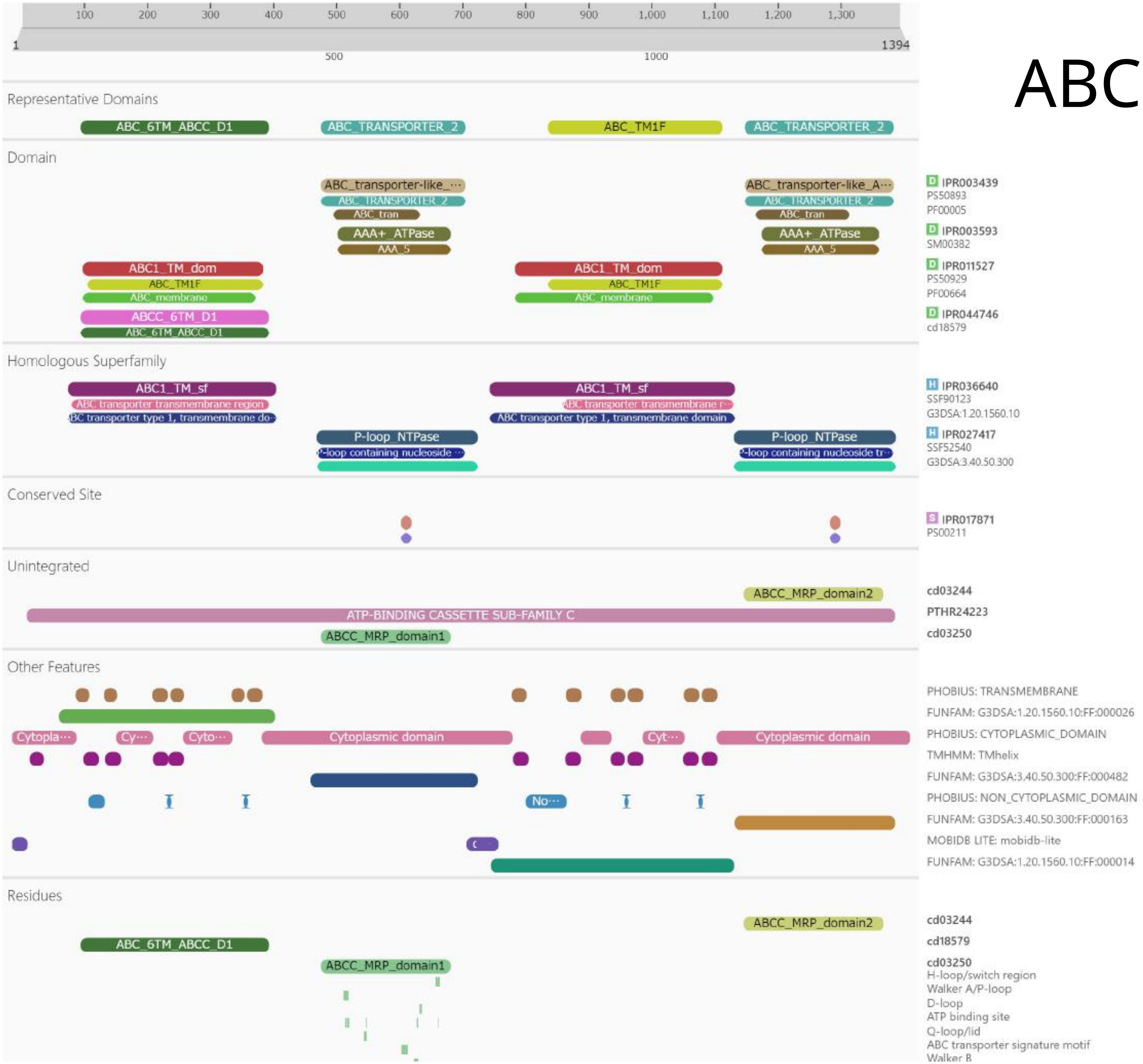

Representative Domains
ABC_6TM_ABCC_D1
ABC_TRANSPORTER_2
ABC_TM1F
Domain
ABC_transporter-like
ABC1_TM_dom
AAA+_ATPase
ABCC_6TM_D1
IPR003439
PS50893
PF00005
IPR003593
SM00382
IPR011527
PS50929
PF00664
IPR044746
cd18579
Homologous Superfamily
ABC1_TM_sf
P-loop_NTPase
IPR036640
SSF90123
G3DSA:1.20.1560.10
IPR027417
SSF52540
G3DSA:3.40.50.300
Conserved Site
IPR017871
PS00211
Unintegrated
ABCC_MRP_domain2
ATP-BINDING CASSETTE SUB-FAMILY C
ABCC_MRP_domain1
cd03244
PTHR24223
cd03250
Other Features
Cytoplasmic domain
PHOBIUS: TRANSMEMBRANE
FUNFAM: G3DSA:1.20.1560.10:FF:000026
PHOBIUS: CYTOPLASMIC_DOMAIN
TMHMM: TMhelix
FUNFAM: G3DSA:3.40.50.300:FF:000482
PHOBIUS: NON_CYTOPLASMIC_DOMAIN
FUNFAM: G3DSA:3.40.50.300:FF:000163
MOBIDB LITE: mobidb-lite
FUNFAM: G3DSA:1.20.1560.10:FF:000014
Residues
cd03244
cd18579
cd03250
H-loop/switch region
Walker A/P-loop
D-loop
ATP binding site
Q-loop/lid
ABC transporter signature motif
Walker B

ABCC5

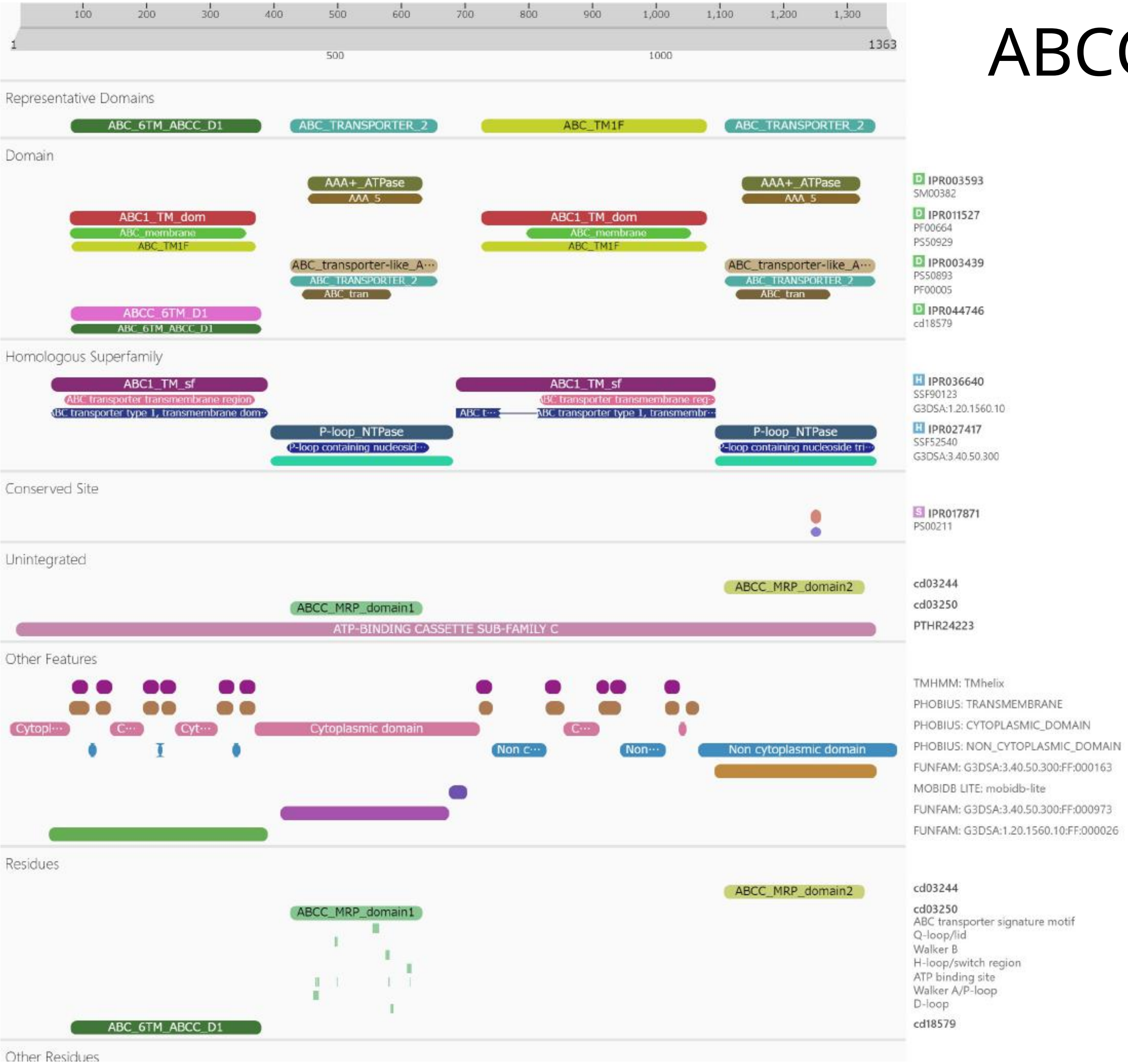

Representative Domains
ABC_6TM_ABCC_D1
ABC_TRANSPORTER_2
ABC_TM1F
Domain
AAA+_ATPase
ABC1_TM_dom
ABC_transporter-like_A···
ABCC_6TM_D1
IPR003593
SM00382
IPR011527
PF00664
PS50929
IPR003439
PS50893
PF00005
IPR044746
cd18579
Homologous Superfamily
ABC1_TM_sf
P-loop_NTPase
IPR036640
SSF90123
G3DSA:1.20.1560.10
IPR027417
SSF52540
G3DSA:3.40.50.300
Conserved Site
IPR017871
PS00211
Unintegrated
ABCC_MRP_domain2
ABCC_MRP_domain1
ATP-BINDING CASSETTE SUB-FAMILY C
cd03244
cd03250
PTHR24223
Other Features
Cytoplasmic domain
Non cytoplasmic domain
TMHMM: TMhelix
PHOBIUS: TRANSMEMBRANE
PHOBIUS: CYTOPLASMIC_DOMAIN
PHOBIUS: NON_CYTOPLASMIC_DOMAIN
FUNFAM: G3DSA:3.40.50.300:FF:000163
MOBIDB LITE: mobidb-lite
FUNFAM: G3DSA:3.40.50.300:FF:000973
FUNFAM: G3DSA:1.20.1560.10:FF:000026
Residues
ABCC_MRP_domain2
ABCC_MRP_domain1
ABC_6TM_ABCC_D1
cd03244
cd03250
ABC transporter signature motif
Q-loop/lid
Walker B
H-loop/switch region
ATP binding site
Walker A/P-loop
D-loop
cd18579
Other Residues

ABCC4

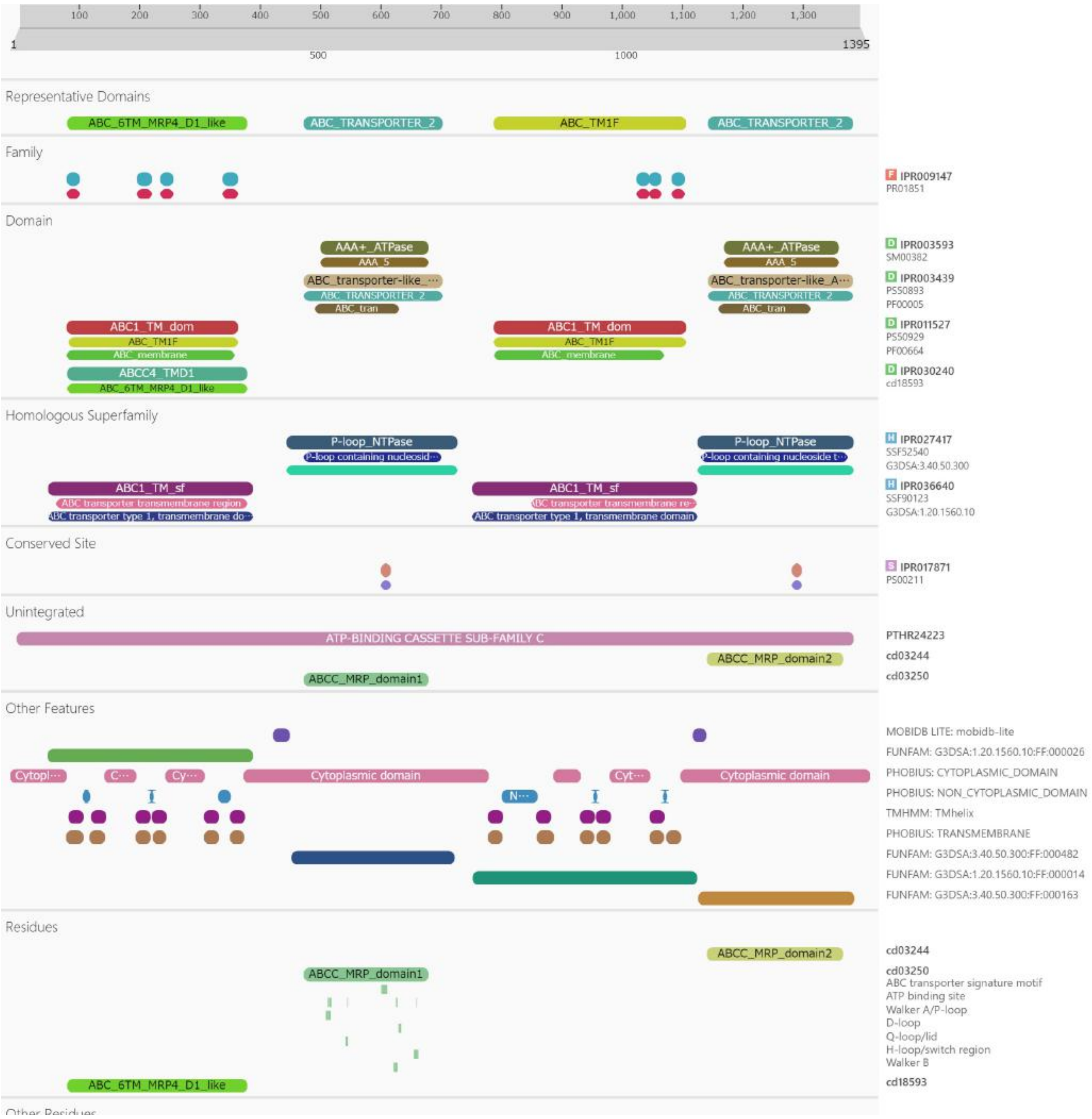

Representative Domains
ABC_6TM_MRP4_D1_like
ABC_TRANSPORTER_2
ABC_TM1F
Family
IPR009147
PR01851
Domain
AAA+_ATPase
ABC_transporter-like
ABC1_TM_dom
ABCC4_TMD1
IPR003593
SM00382
IPR003439
PS50893
PF00005
IPR011527
PS50929
PF00664
IPR030240
cd18593
Homologous Superfamily
P-loop_NTPase
ABC1_TM_sf
IPR027417
SSF52540
G3DSA:3.40.50.300
IPR036640
SSF90123
G3DSA:1.20.1560.10
Conserved Site
IPR017871
PS00211
Unintegrated
ATP-BINDING CASSETTE SUB-FAMILY C
ABCC_MRP_domain2
ABCC_MRP_domain1
PTHR24223
cd03244
cd03250
Other Features
Cytoplasmic domain
MOBIDB LITE: mobidb-lite
FUNFAM: G3DSA:1.20.1560.10:FF:000026
PHOBIUS: CYTOPLASMIC_DOMAIN
PHOBIUS: NON_CYTOPLASMIC_DOMAIN
TMHMM: TMhelix
PHOBIUS: TRANSMEMBRANE
FUNFAM: G3DSA:3.40.50.300:FF:000482
FUNFAM: G3DSA:1.20.1560.10:FF:000014
FUNFAM: G3DSA:3.40.50.300:FF:000163
Residues
ABC transporter signature motif
ATP binding site
Walker A/P-loop
D-loop
Q-loop/lid
H-loop/switch region
Walker B
cd18593

ABCC4-like1

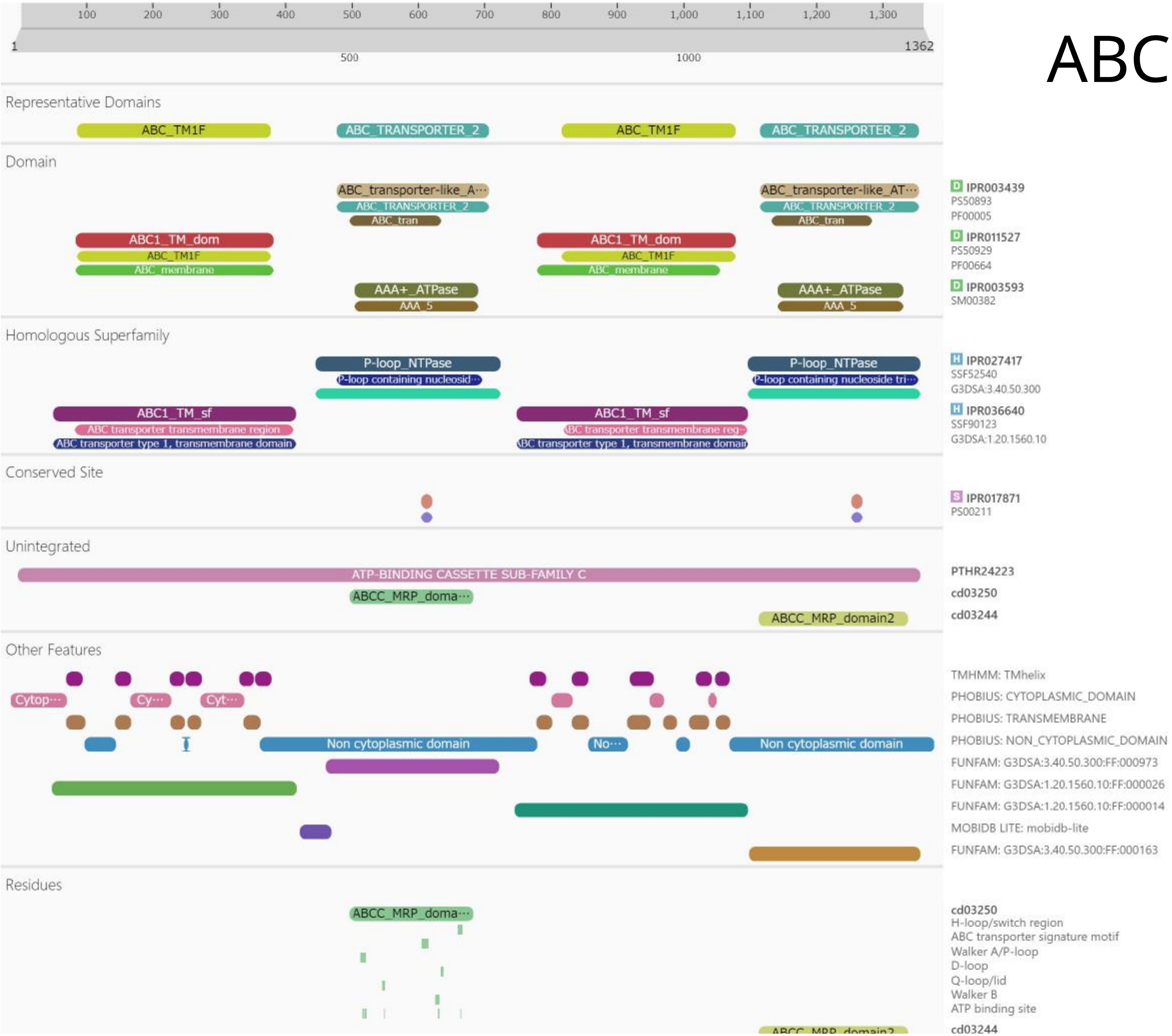

Representative Domains
ABC_TM1F
ABC_TRANSPORTER_2
Domain
IPR003439
PS50893
PF00005
IPR011527
PS50929
PF00664
IPR003593
SM00382
ABC1_TM_dom
ABC_membrane
AAA+_ATPase
AAA_5
ABC_tran
Homologous Superfamily
P-loop_NTPase
ABC1_TM_sf
ABC transporter transmembrane region
ABC transporter type 1, transmembrane domain
IPR027417
SSF52540
G3DSA:3.40.50.300
IPR036640
SSF90123
G3DSA:1.20.1560.10
Conserved Site
IPR017871
PS00211
Unintegrated
ATP-BINDING CASSETTE SUB-FAMILY C
ABCC_MRP_domain2
PTHR24223
cd03250
cd03244
Other Features
Non cytoplasmic domain
TMHMM: TMhelix
PHOBIUS: CYTOPLASMIC_DOMAIN
PHOBIUS: TRANSMEMBRANE
PHOBIUS: NON_CYTOPLASMIC_DOMAIN
FUNFAM: G3DSA:3.40.50.300:FF:000973
FUNFAM: G3DSA:1.20.1560.10:FF:000026
FUNFAM: G3DSA:1.20.1560.10:FF:000014
MOBIDB LITE: mobidb-lite
FUNFAM: G3DSA:3.40.50.300:FF:000163
Residues
cd03250
H-loop/switch region
ABC transporter signature motif
Walker A/P-loop
D-loop
Q-loop/lid
Walker B
ATP binding site
cd03244

ABCC4-like2

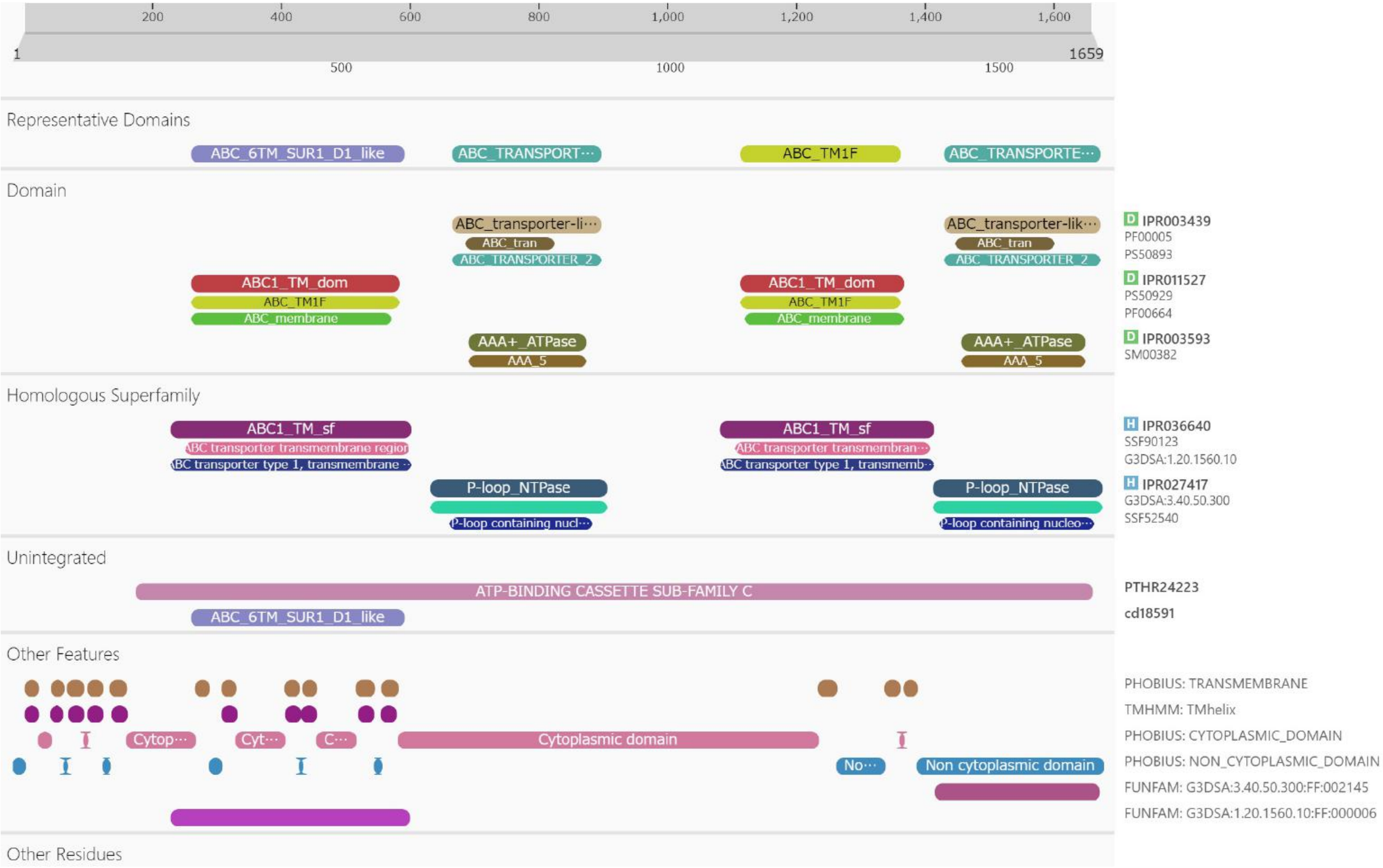

200
400
600
800
1,000
1,200
1,400
1,600
1
1659
500
1000
1500
Representative Domains
ABC_6TM_SUR1_D1_like
ABC_TRANSPORT···
ABC_TM1F
ABC_TRANSPORTE···
Domain
ABC_transporter-li···
ABC_tran
ABC_TRANSPORTER_2
ABC_transporter-lik···
ABC1_TM_dom
ABC_TM1F
ABC_membrane
AAA+_ATPase
AAA_5
IPR003439
PF00005
PS50893
IPR011527
PS50929
PF00664
IPR003593
SM00382
Homologous Superfamily
ABC1_TM_sf
ABC transporter transmembrane region
ABC transporter type 1, transmembrane
ABC transporter transmembran···
ABC transporter type 1, transmemb···
P-loop_NTPase
P-loop containing nucl···
P-loop containing nucleo···
IPR036640
SSF90123
G3DSA:1.20.1560.10
IPR027417
G3DSA:3.40.50.300
SSF52540
Unintegrated
ATP-BINDING CASSETTE SUB-FAMILY C
ABC_6TM_SUR1_D1_like
PTHR24223
cd18591
Other Features
Cytop···
Cyt···
C···
Cytoplasmic domain
No···
Non cytoplasmic domain
PHOBIUS: TRANSMEMBRANE
TMHMM: TMhelix
PHOBIUS: CYTOPLASMIC_DOMAIN
PHOBIUS: NON_CYTOPLASMIC_DOMAIN
FUNFAM: G3DSA:3.40.50.300:FF:002145
FUNFAM: G3DSA:1.20.1560.10:FF:000006
Other Residues

ABCC9

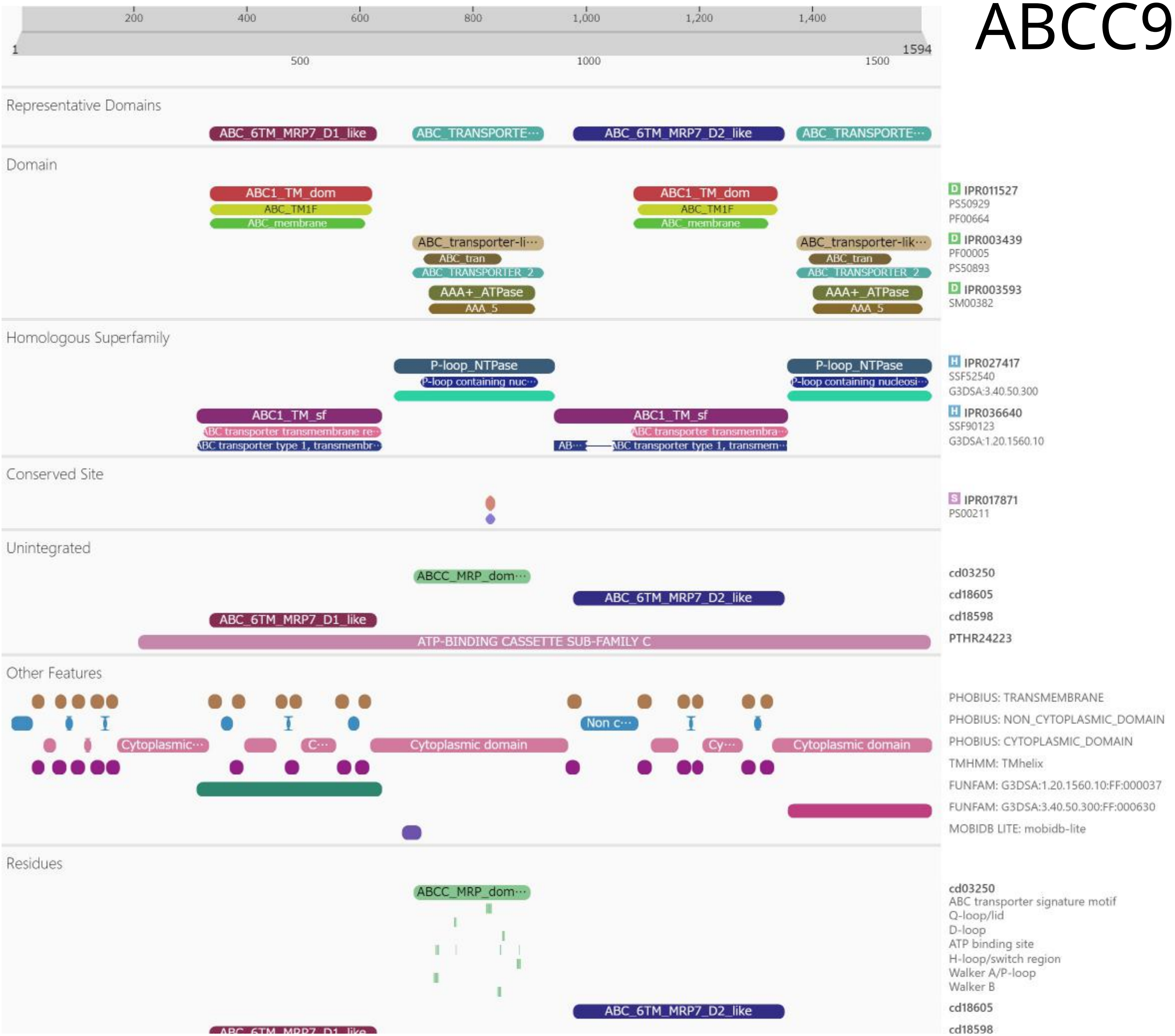

200
400
600
800
1,000
1,200
1,400
1
1594
500
1000
1500
Representative Domains
ABC_6TM_MRP7_D1_like
ABC_TRANSPORTE···
ABC_6TM_MRP7_D2_like
ABC_TRANSPORTE···
Domain
ABC1_TM_dom
ABC_TM1F
ABC_membrane
ABC_transporter-li···
ABC_tran
ABC_TRANSPORTER_2
AAA+_ATPase
AAA_5
ABC_transporter-lik···
IPR011527
PS50929
PF00664
IPR003439
PF00005
PS50893
IPR003593
SM00382
Homologous Superfamily
P-loop_NTPase
P-loop containing nuc···
P-loop containing nucleosi···
ABC1_TM_sf
ABC transporter transmembrane re···
ABC transporter type 1, transmembr···
AB···
ABC transporter type 1, transmem···
IPR027417
SSF52540
G3DSA:3.40.50.300
IPR036640
SSF90123
G3DSA:1.20.1560.10
Conserved Site
IPR017871
PS00211
Unintegrated
ABCC_MRP_dom···
ABC_6TM_MRP7_D2_like
ABC_6TM_MRP7_D1_like
ATP-BINDING CASSETTE SUB-FAMILY C
cd03250
cd18605
cd18598
PTHR24223
Other Features
Non c···
Cytoplasmic···
C···
Cytoplasmic domain
Cy···
PHOBIUS: TRANSMEMBRANE
PHOBIUS: NON_CYTOPLASMIC_DOMAIN
PHOBIUS: CYTOPLASMIC_DOMAIN
TMHMM: TMhelix
FUNFAM: G3DSA:1.20.1560.10:FF:000037
FUNFAM: G3DSA:3.40.50.300:FF:000630
MOBIDB LITE: mobidb-lite
Residues
ABCC_MRP_dom···
ABC_6TM_MRP7_D2_like
cd03250
ABC transporter signature motif
Q-loop/lid
D-loop
ATP binding site
H-loop/switch region
Walker A/P-loop
Walker B
cd18605
cd18598

ABCC10

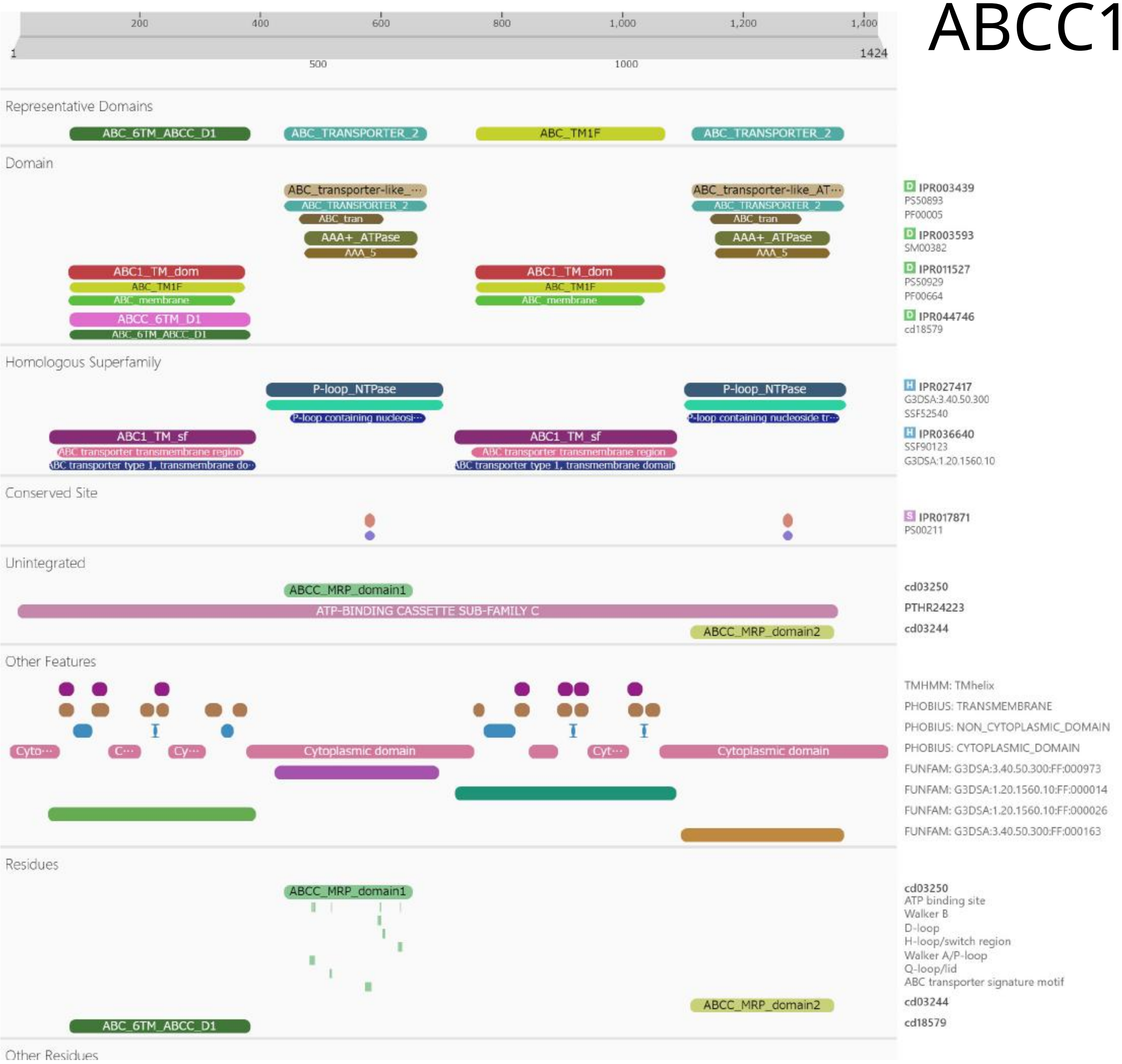

200
400
600
800
1,000
1,200
1,400
1
1424
500
1000
Representative Domains
ABC_6TM_ABCC_D1
ABC_TRANSPORTER_2
ABC_TM1F
ABC_TRANSPORTER_2
Domain
ABC_transporter-like_...
ABC_TRANSPORTER_2
ABC_tran
AAA+_ATPase
AAA_5
ABC_transporter-like_AT...
ABC1_TM_dom
ABC_TM1F
ABC_membrane
ABCC_6TM_D1
ABC_6TM_ABCC_D1
IPR003439
PS50893
PF00005
IPR003593
SM00382
IPR011527
PS50929
PF00664
IPR044746
cd18579
Homologous Superfamily
P-loop_NTPase
P-loop containing nucleosi...
P-loop containing nucleoside tr...
ABC1_TM_sf
ABC transporter transmembrane region
ABC transporter type 1, transmembrane do...
ABC transporter type 1, transmembrane domain
IPR027417
G3DSA:3.40.50.300
SSF52540
IPR036640
SSF90123
G3DSA:1.20.1560.10
Conserved Site
IPR017871
PS00211
Unintegrated
ABCC_MRP_domain1
ATP-BINDING CASSETTE SUB-FAMILY C
ABCC_MRP_domain2
cd03250
PTHR24223
cd03244
Other Features
Cyto...
C...
Cy...
Cytoplasmic domain
Cyt...
Cytoplasmic domain
TMHMM: TMhelix
PHOBIUS: TRANSMEMBRANE
PHOBIUS: NON_CYTOPLASMIC_DOMAIN
PHOBIUS: CYTOPLASMIC_DOMAIN
FUNFAM: G3DSA:3.40.50.300:FF:000973
FUNFAM: G3DSA:1.20.1560.10:FF:000014
FUNFAM: G3DSA:1.20.1560.10:FF:000026
FUNFAM: G3DSA:3.40.50.300:FF:000163
Residues
ABCC_MRP_domain1
ABCC_MRP_domain2
ABC_6TM_ABCC_D1
cd03250
ATP binding site
Walker B
D-loop
H-loop/switch region
Walker A/P-loop
Q-loop/lid
ABC transporter signature motif
cd03244
cd18579
Other Residues

ABCC11

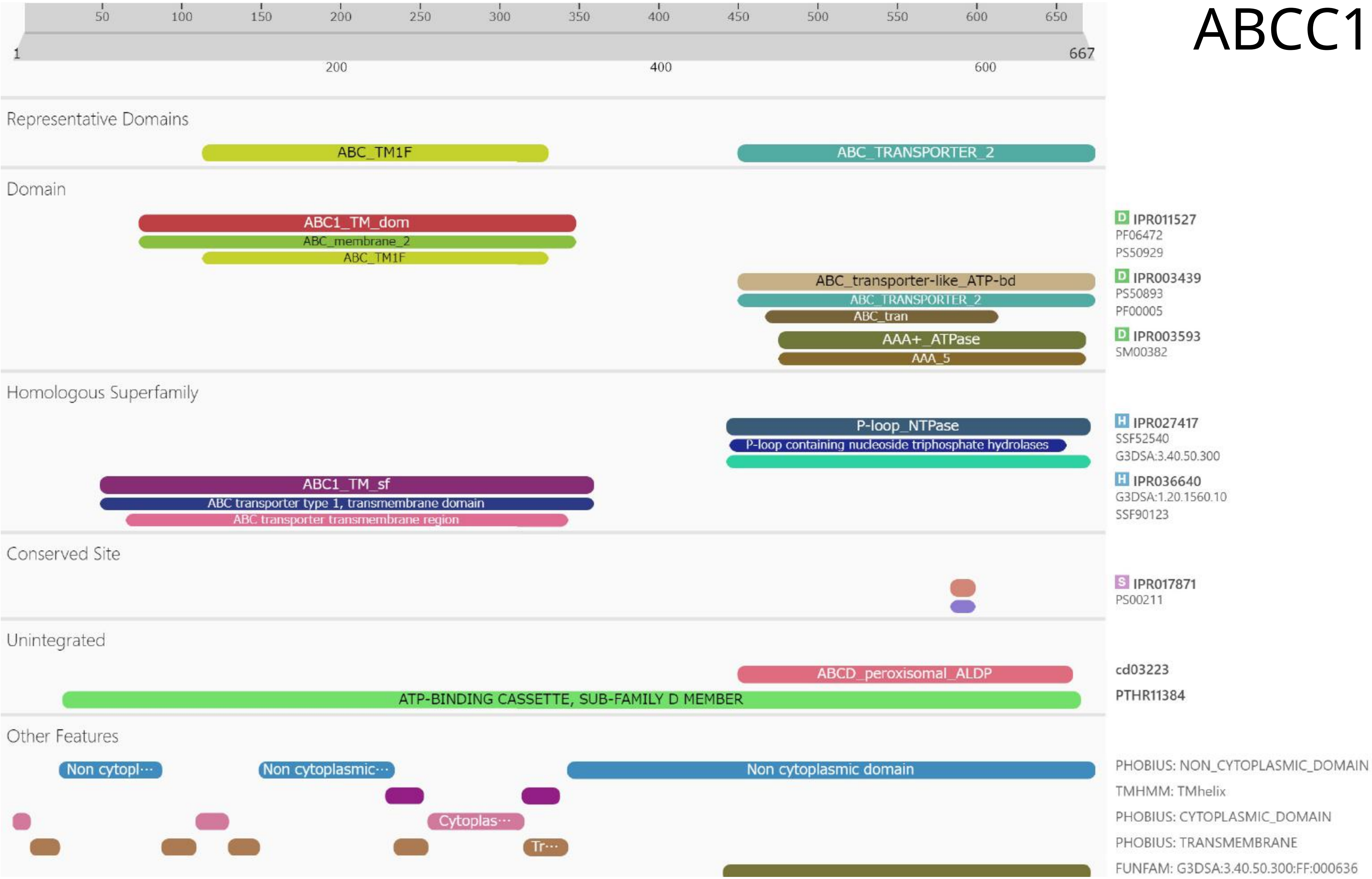

50 100 150 200 250 300 350 400 450 500 550 600 650
1 667
200 400 600
Representative Domains
ABC_TM1F
ABC_TRANSPORTER_2
Domain
ABC1_TM_dom
ABC_membrane_2
ABC_TM1F
ABC_transporter-like_ATP-bd
ABC_TRANSPORTER_2
ABC_tran
AAA+_ATPase
AAA_5
D IPR011527
PF06472
PS50929
D IPR003439
PS50893
PF00005
D IPR003593
SM00382
Homologous Superfamily
P-loop_NTPase
P-loop containing nucleoside triphosphate hydrolases
ABC1_TM_sf
ABC transporter type 1, transmembrane domain
ABC transporter transmembrane region
H IPR027417
SSF52540
G3DSA:3.40.50.300
H IPR036640
G3DSA:1.20.1560.10
SSF90123
Conserved Site
S IPR017871
PS00211
Unintegrated
ABCD_peroxisomal_ALDP
ATP-BINDING CASSETTE, SUB-FAMILY D MEMBER
cd03223
PTHR11384
Other Features
Non cytopl···
Non cytoplasmic···
Non cytoplasmic domain
Cytoplas···
Tr···
PHOBIUS: NON_CYTOPLASMIC_DOMAIN
TMHMM: TMhelix
PHOBIUS: CYTOPLASMIC_DOMAIN
PHOBIUS: TRANSMEMBRANE
FUNFAM: G3DSA:3.40.50.300:FF:000636

ABCD1

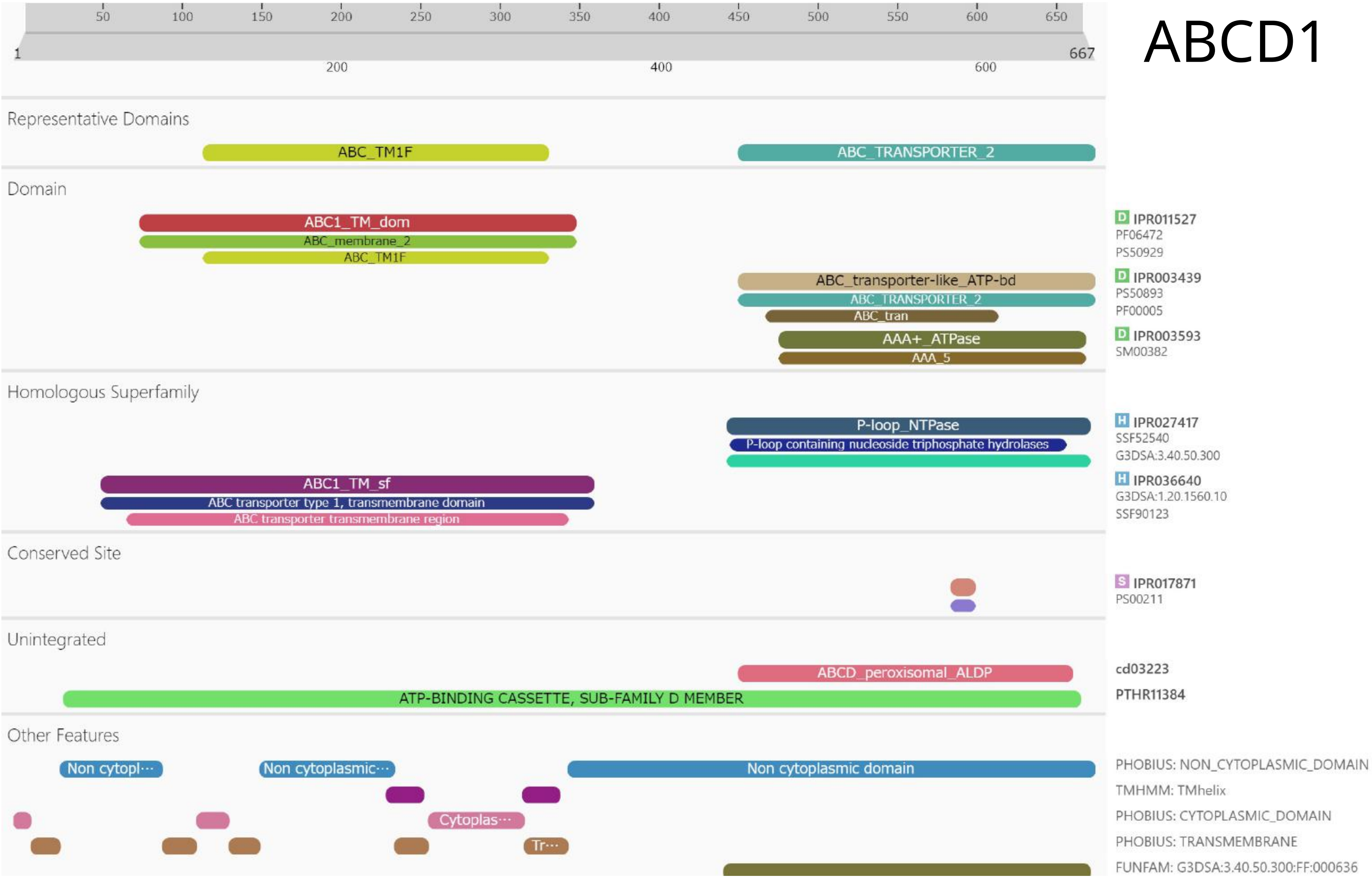

50
100
150
200
250
300
350
400
450
500
550
600
650
1
667
200
400
600
Representative Domains
ABC_TM1F
ABC_TRANSPORTER_2
Domain
ABC1_TM_dom
ABC_membrane_2
ABC_TM1F
ABC_transporter-like_ATP-bd
ABC_TRANSPORTER_2
ABC_tran
AAA+_ATPase
AAA_5
Homologous Superfamily
P-loop_NTPase
P-loop containing nucleoside triphosphate hydrolases
ABC1_TM_sf
ABC transporter type 1, transmembrane domain
ABC transporter transmembrane region
Conserved Site
Unintegrated
ABCD_peroxisomal_ALDP
ATP-BINDING CASSETTE, SUB-FAMILY D MEMBER
Other Features
Non cytopl···
Non cytoplasmic···
Non cytoplasmic domain
Cytoplas···
Tr···
D IPR011527
PF06472
PS50929
D IPR003439
PS50893
PF00005
D IPR003593
SM00382
H IPR027417
SSF52540
G3DSA:3.40.50.300
H IPR036640
G3DSA:1.20.1560.10
SSF90123
S IPR017871
PS00211
cd03223
PTHR11384
PHOBIUS: NON_CYTOPLASMIC_DOMAIN
TMHMM: TMhelix
PHOBIUS: CYTOPLASMIC_DOMAIN
PHOBIUS: TRANSMEMBRANE
FUNFAM: G3DSA:3.40.50.300:FF:000636

ABCD2

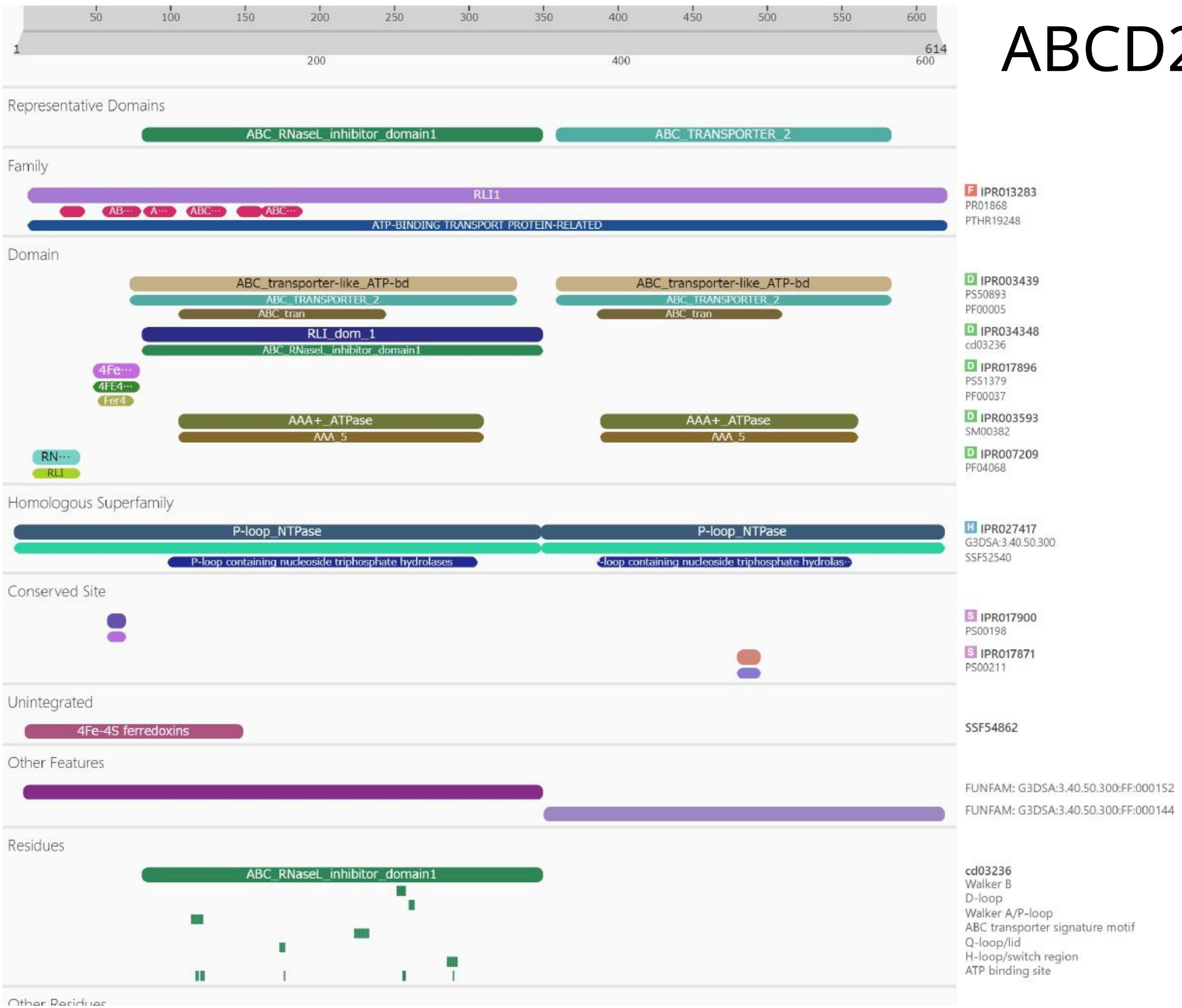

Representative Domains
ABC_RNaseL_inhibitor_domain1
ABC_TRANSPORTER_2
Family
RLI1
ATP-BINDING TRANSPORT PROTEIN-RELATED
IPR013283
PR01868
PTHR19248
Domain
ABC_transporter-like_ATP-bd
ABC_TRANSPORTER_2
ABC_tran
RLI_dom_1
ABC_RNaseL_inhibitor_domain1
AAA+_ATPase
AAA_5
RLI
IPR003439
PS50893
PF00005
IPR034348
cd03236
IPR017896
PS51379
PF00037
IPR003593
SM00382
IPR007209
PF04068
Homologous Superfamily
P-loop_NTPase
P-loop containing nucleoside triphosphate hydrolases
IPR027417
G3DSA:3.40.50.300
SSF52540
Conserved Site
IPR017900
PS00198
IPR017871
PS00211
Unintegrated
4Fe-4S ferredoxins
SSF54862
Other Features
FUNFAM: G3DSA:3.40.50.300:FF:000152
FUNFAM: G3DSA:3.40.50.300:FF:000144
Residues
ABC_RNaseL_inhibitor_domain1
cd03236
Walker B
D-loop
Walker A/P-loop
ABC transporter signature motif
Q-loop/lid
H-loop/switch region
ATP binding site

ABCE1

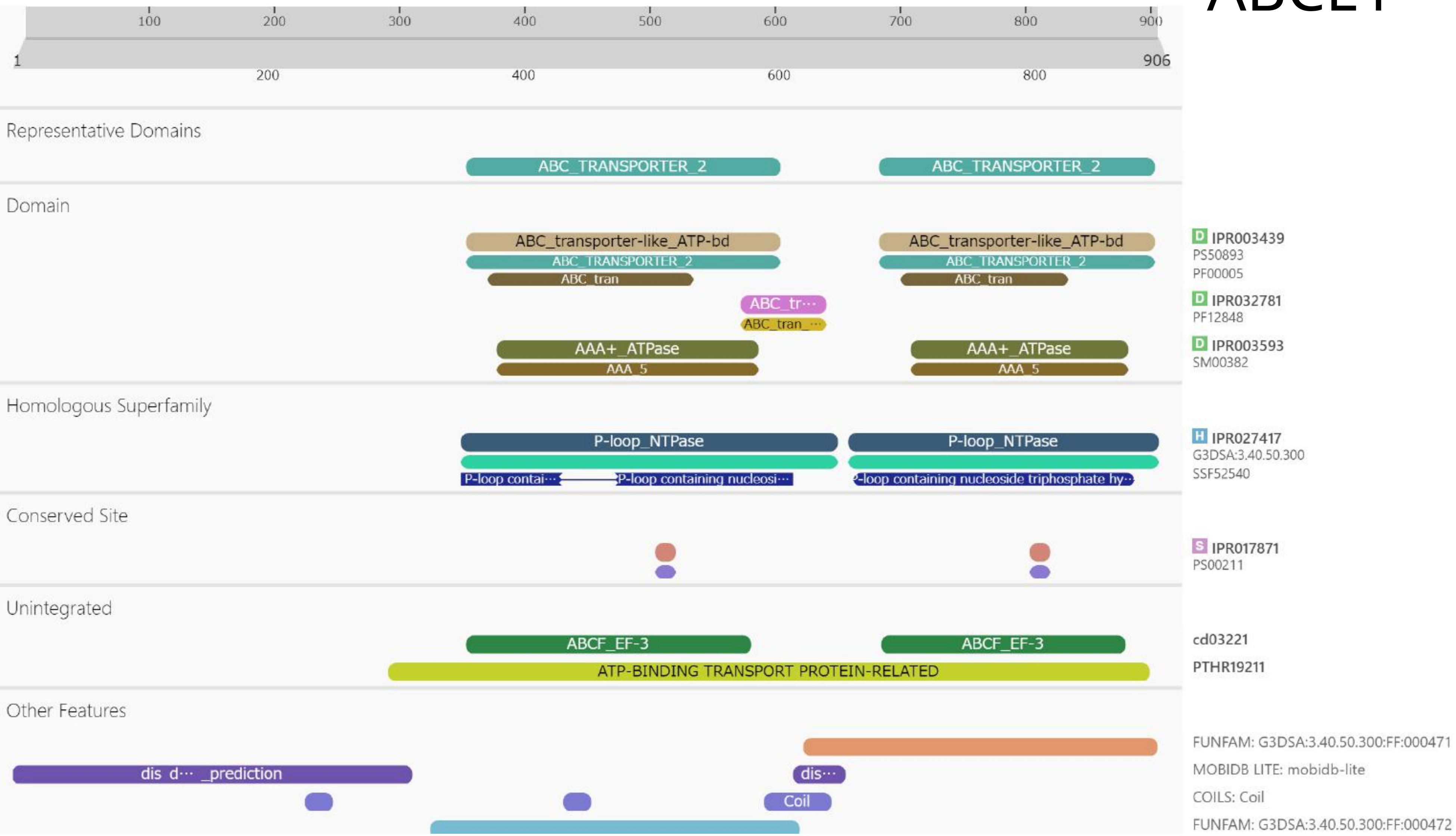

100
200
300
400
500
600
700
800
900
1
906
200
400
600
800
Representative Domains
ABC_TRANSPORTER_2
ABC_TRANSPORTER_2
Domain
ABC_transporter-like_ATP-bd
ABC_TRANSPORTER_2
ABC_tran
ABC_tr···
ABC_tran_···
AAA+_ATPase
AAA_5
IPR003439
PS50893
PF00005
IPR032781
PF12848
IPR003593
SM00382
Homologous Superfamily
P-loop_NTPase
P-loop contai···
P-loop containing nucleosi···
P-loop containing nucleoside triphosphate hy···
IPR027417
G3DSA:3.40.50.300
SSF52540
Conserved Site
IPR017871
PS00211
Unintegrated
ABCF_EF-3
ATP-BINDING TRANSPORT PROTEIN-RELATED
cd03221
PTHR19211
Other Features
dis d··· _prediction
dis···
Coil
FUNFAM: G3DSA:3.40.50.300:FF:000471
MOBIDB LITE: mobidb-lite
COILS: Coil
FUNFAM: G3DSA:3.40.50.300:FF:000472

ABCF1

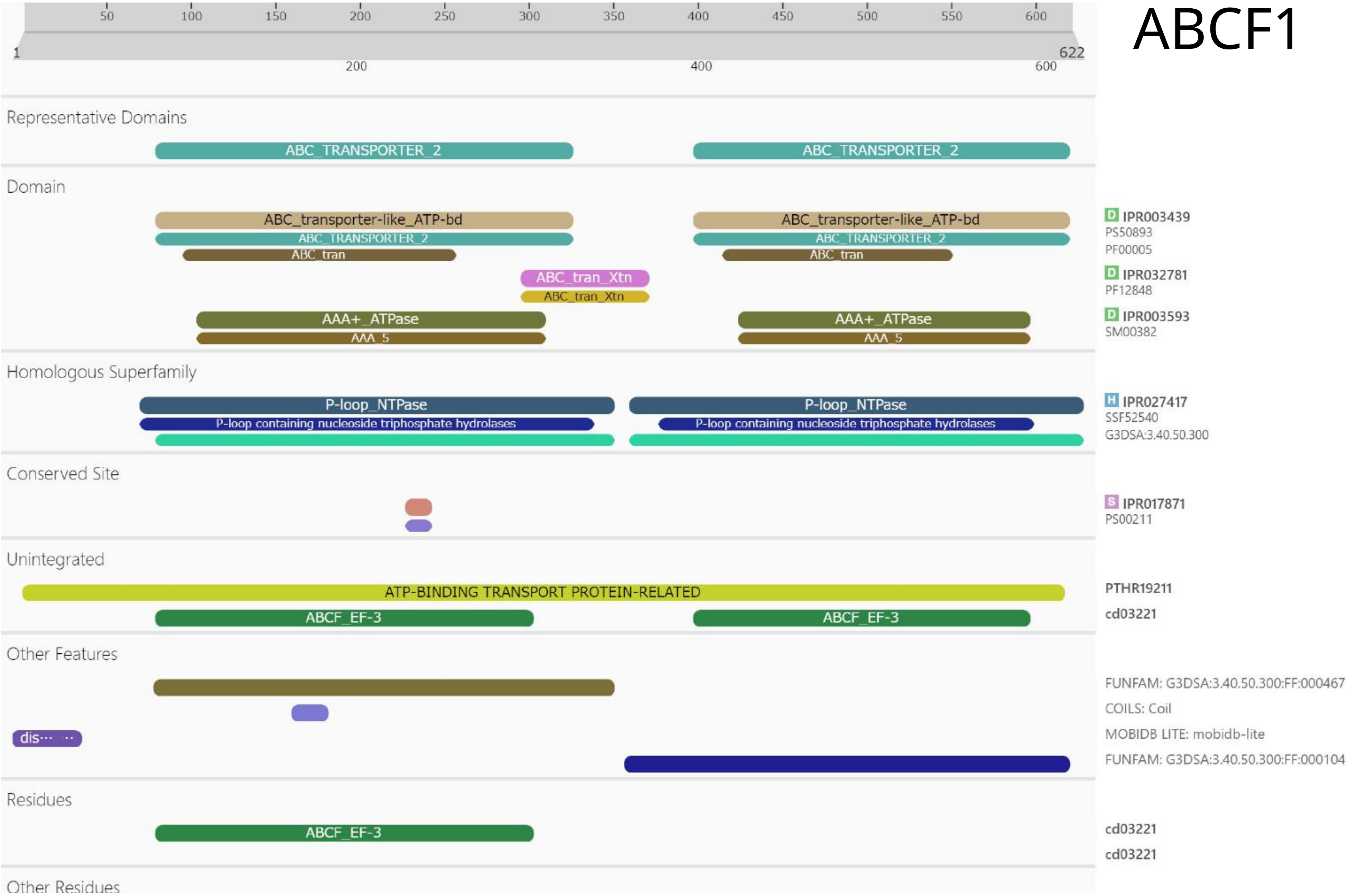
50
100
150
200
250
300
350
400
450
500
550
600
1
622
200
400
600
Representative Domains
ABC_TRANSPORTER_2
ABC_TRANSPORTER_2
Domain
ABC_transporter-like_ATP-bd
ABC_TRANSPORTER_2
ABC_tran
ABC_transporter-like_ATP-bd
ABC_TRANSPORTER_2
ABC_tran
ABC_tran_Xtn
ABC_tran_Xtn
AAA+_ATPase
AAA_5
AAA+_ATPase
AAA_5
D IPR003439
PS50893
PF00005
D IPR032781
PF12848
D IPR003593
SM00382
Homologous Superfamily
P-loop_NTPase
P-loop containing nucleoside triphosphate hydrolases
P-loop_NTPase
P-loop containing nucleoside triphosphate hydrolases
H IPR027417
SSF52540
G3DSA:3.40.50.300
Conserved Site
S IPR017871
PS00211
Unintegrated
ATP-BINDING TRANSPORT PROTEIN-RELATED
ABCF_EF-3
ABCF_EF-3
PTHR19211
cd03221
Other Features
dis··· ··
FUNFAM: G3DSA:3.40.50.300:FF:000467
COILS: Coil
MOBIDB LITE: mobidb-lite
FUNFAM: G3DSA:3.40.50.300:FF:000104
Residues
ABCF_EF-3
cd03221
cd03221
Other Residues

ABCF2

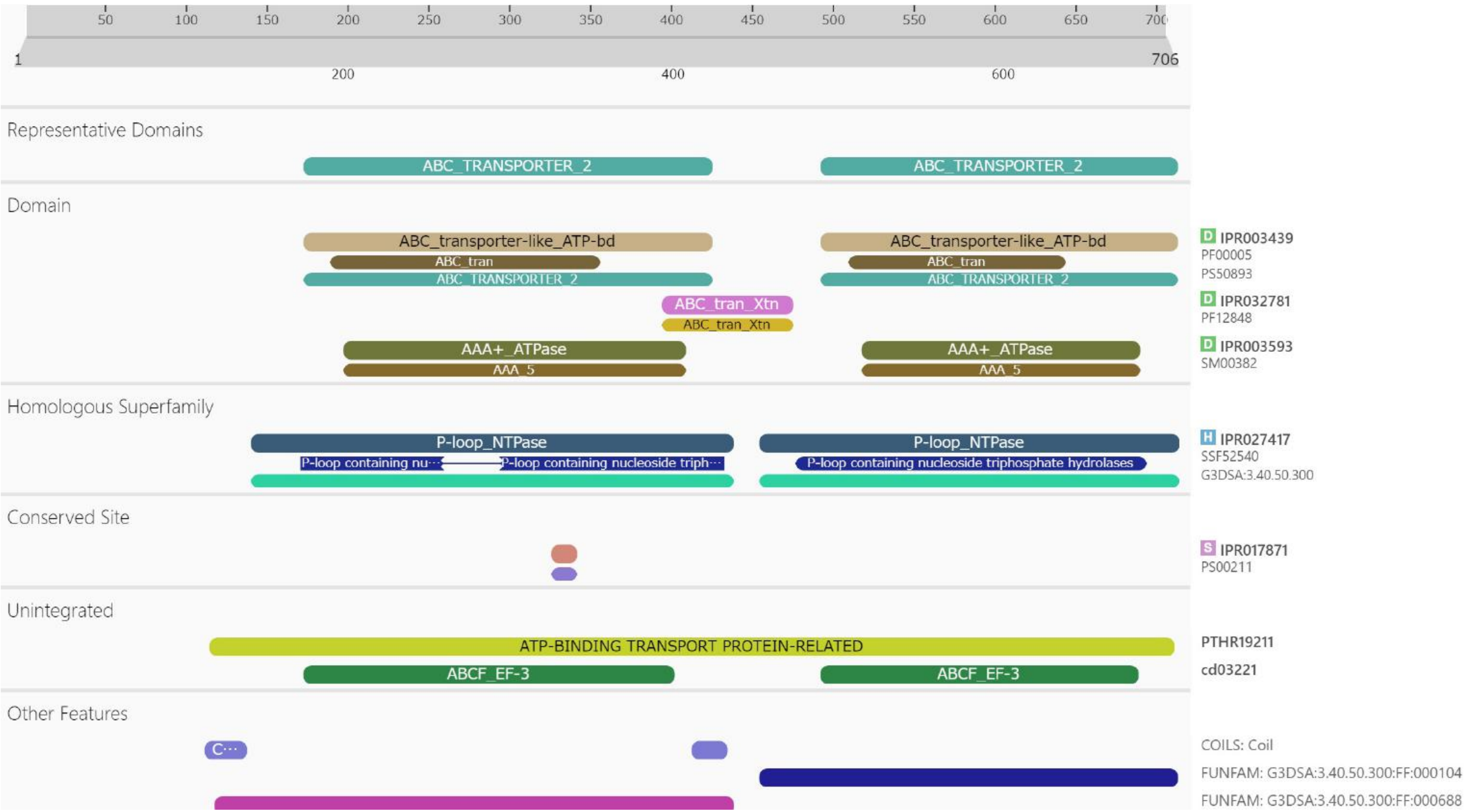

50 100 150 200 250 300 350 400 450 500 550 600 650 700
1 706
200 400 600
Representative Domains
ABC_TRANSPORTER_2
ABC_TRANSPORTER_2
Domain
ABC_transporter-like_ATP-bd
ABC_tran
ABC_TRANSPORTER_2
ABC_tran_Xtn
ABC_tran_Xtn
AAA+_ATPase
AAA_5
IPR003439
PF00005
PS50893
IPR032781
PF12848
IPR003593
SM00382
Homologous Superfamily
P-loop_NTPase
P-loop containing nucleoside triphosphate hydrolases
IPR027417
SSF52540
G3DSA:3.40.50.300
Conserved Site
IPR017871
PS00211
Unintegrated
ATP-BINDING TRANSPORT PROTEIN-RELATED
ABCF_EF-3
PTHR19211
cd03221
Other Features
COILS: Coil
FUNFAM: G3DSA:3.40.50.300:FF:000104
FUNFAM: G3DSA:3.40.50.300:FF:000688

ABCF3

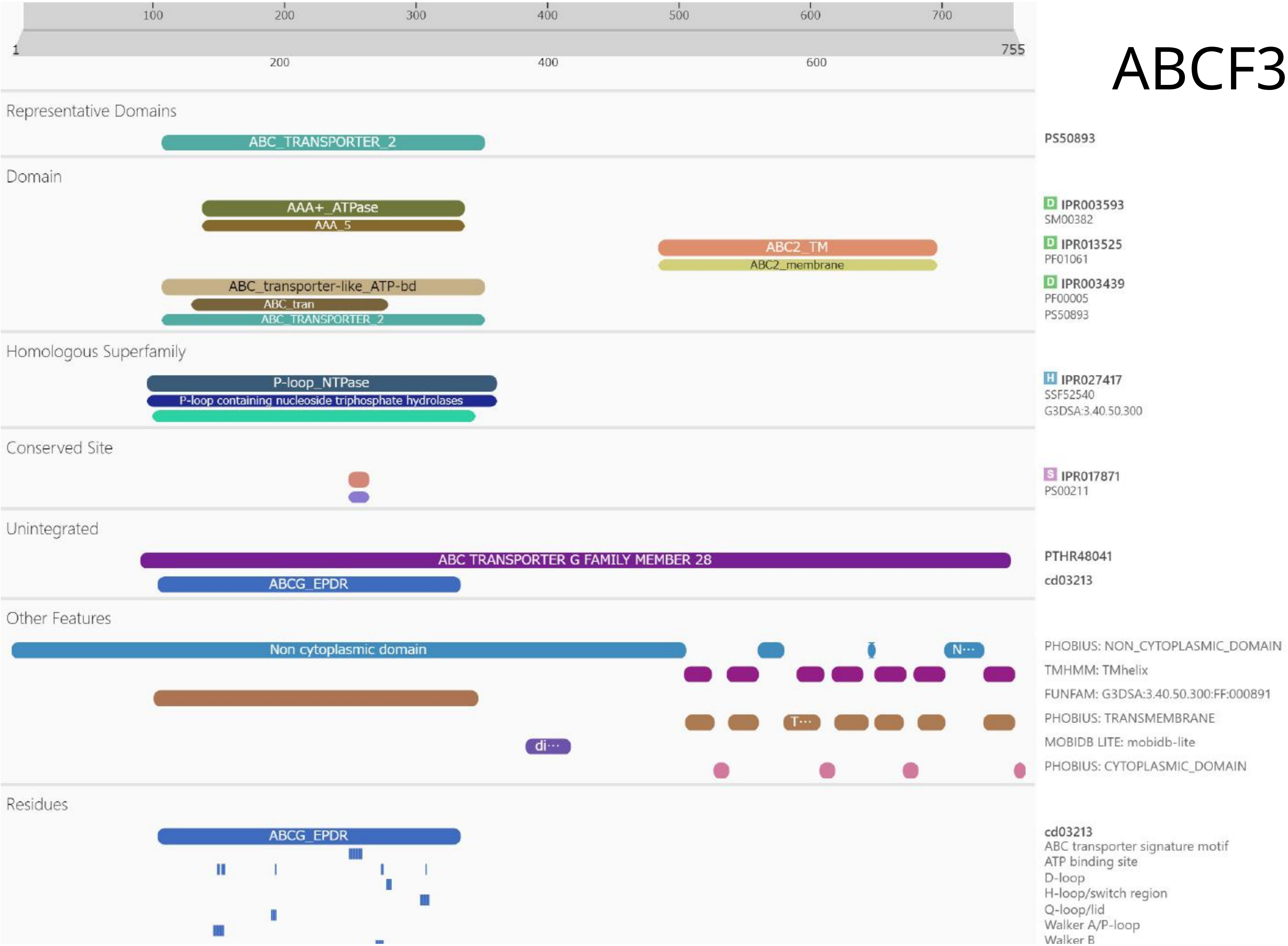

100 200 300 400 500 600 700
1 755
200 400 600
Representative Domains
ABC_TRANSPORTER_2
PS50893
Domain
AAA+_ATPase
AAA_5
ABC2_TM
ABC2_membrane
ABC_transporter-like_ATP-bd
ABC_tran
ABC_TRANSPORTER_2
IPR003593
SM00382
IPR013525
PF01061
IPR003439
PF00005
PS50893
Homologous Superfamily
P-loop_NTPase
P-loop containing nucleoside triphosphate hydrolases
IPR027417
SSF52540
G3DSA:3.40.50.300
Conserved Site
IPR017871
PS00211
Unintegrated
ABC TRANSPORTER G FAMILY MEMBER 28
ABCG_EPDR
PTHR48041
cd03213
Other Features
Non cytoplasmic domain
PHOBIUS: NON_CYTOPLASMIC_DOMAIN
TMHMM: TMhelix
FUNFAM: G3DSA:3.40.50.300:FF:000891
PHOBIUS: TRANSMEMBRANE
MOBIDB LITE: mobidb-lite
PHOBIUS: CYTOPLASMIC_DOMAIN
Residues
ABCG_EPDR
cd03213
ABC transporter signature motif
ATP binding site
D-loop
H-loop/switch region
Q-loop/lid
Walker A/P-loop
Walker B

ABCG1

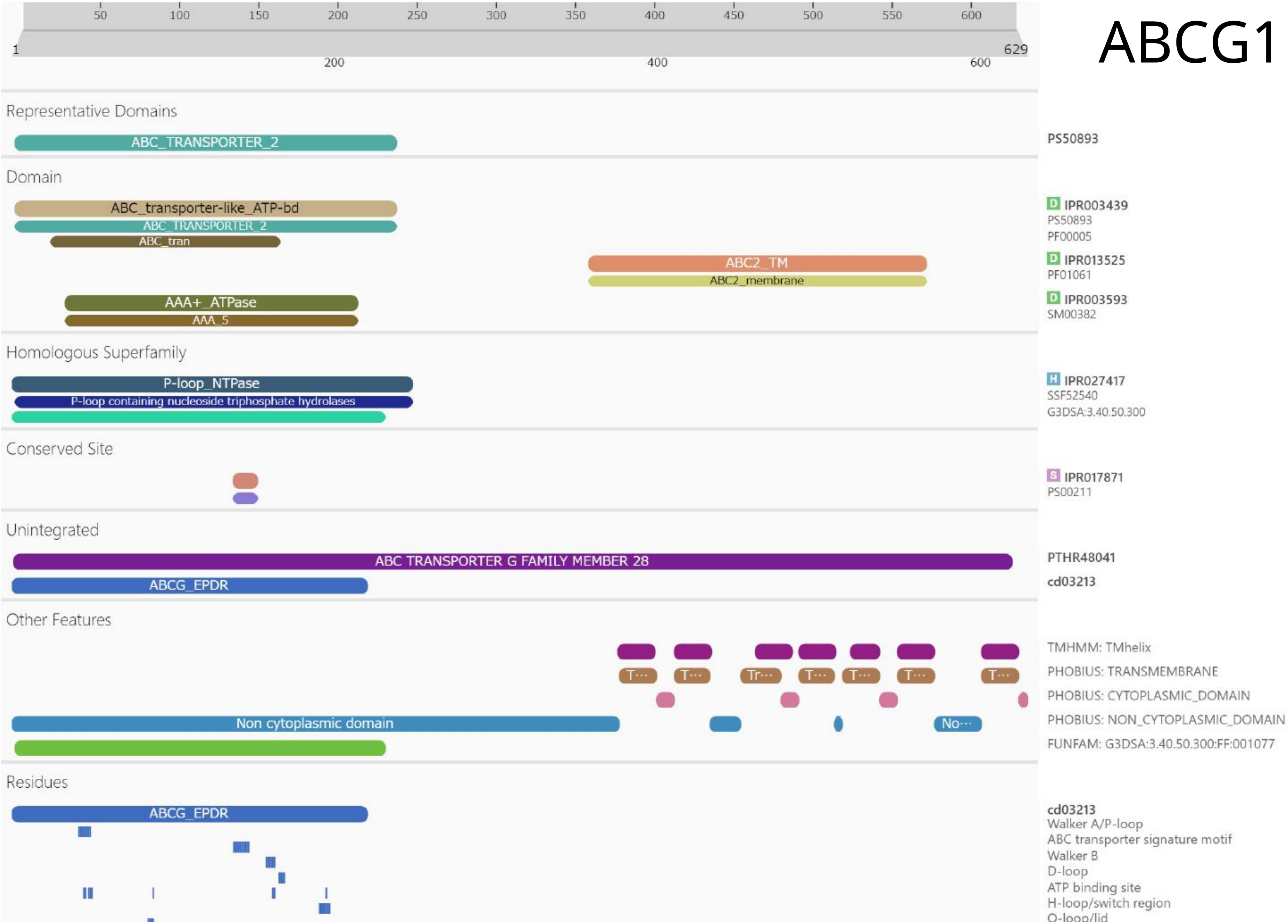

1
629
Representative Domains
ABC_TRANSPORTER_2
PS50893
Domain
ABC_transporter-like_ATP-bd
ABC_TRANSPORTER_2
ABC_tran
ABC2_TM
ABC2_membrane
AAA+_ATPase
AAA_5
IPR003439
PS50893
PF00005
IPR013525
PF01061
IPR003593
SM00382
Homologous Superfamily
P-loop_NTPase
P-loop containing nucleoside triphosphate hydrolases
IPR027417
SSF52540
G3DSA:3.40.50.300
Conserved Site
IPR017871
PS00211
Unintegrated
ABC TRANSPORTER G FAMILY MEMBER 28
ABCG_EPDR
PTHR48041
cd03213
Other Features
Non cytoplasmic domain
TMHMM: TMhelix
PHOBIUS: TRANSMEMBRANE
PHOBIUS: CYTOPLASMIC_DOMAIN
PHOBIUS: NON_CYTOPLASMIC_DOMAIN
FUNFAM: G3DSA:3.40.50.300:FF:001077
Residues
ABCG_EPDR
cd03213
Walker A/P-loop
ABC transporter signature motif
Walker B
D-loop
ATP binding site
H-loop/switch region
Q-loop/lid

ABCG2

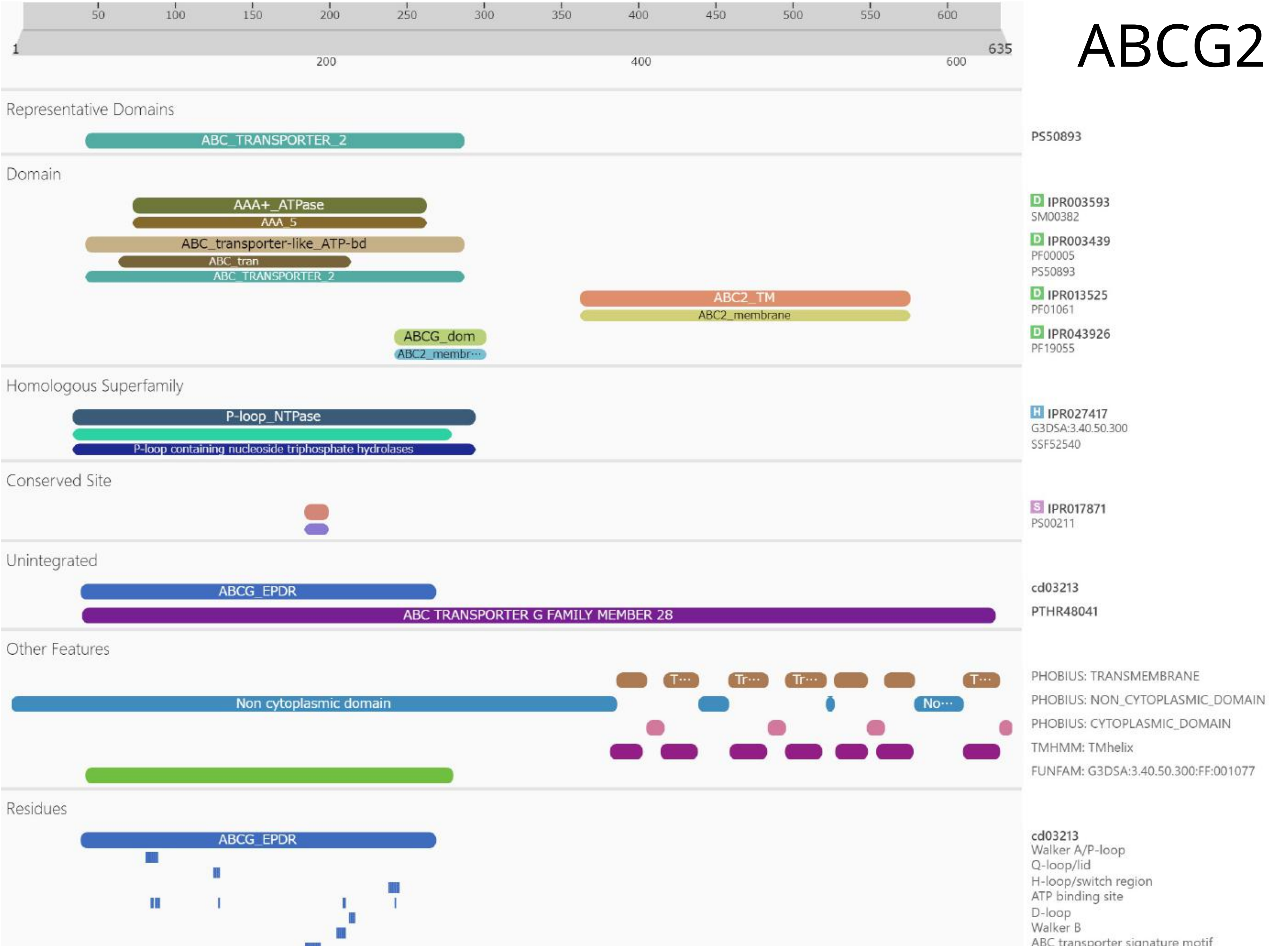

50
100
150
200
250
300
350
400
450
500
550
600
1
635
200
400
600
Representative Domains
ABC_TRANSPORTER_2
PS50893
Domain
AAA+_ATPase
AAA_5
ABC_transporter-like_ATP-bd
ABC_tran
ABC_TRANSPORTER_2
ABC2_TM
ABC2_membrane
ABCG_dom
ABC2_membr···
IPR003593
SM00382
IPR003439
PF00005
PS50893
IPR013525
PF01061
IPR043926
PF19055
Homologous Superfamily
P-loop_NTPase
P-loop containing nucleoside triphosphate hydrolases
IPR027417
G3DSA:3.40.50.300
SSF52540
Conserved Site
IPR017871
PS00211
Unintegrated
ABCG_EPDR
ABC TRANSPORTER G FAMILY MEMBER 28
cd03213
PTHR48041
Other Features
Non cytoplasmic domain
No···
T···
Tr···
PHOBIUS: TRANSMEMBRANE
PHOBIUS: NON_CYTOPLASMIC_DOMAIN
PHOBIUS: CYTOPLASMIC_DOMAIN
TMHMM: TMhelix
FUNFAM: G3DSA:3.40.50.300:FF:001077
Residues
ABCG_EPDR
cd03213
Walker A/P-loop
Q-loop/lid
H-loop/switch region
ATP binding site
D-loop
Walker B
ABC transporter signature motif

ABCG3

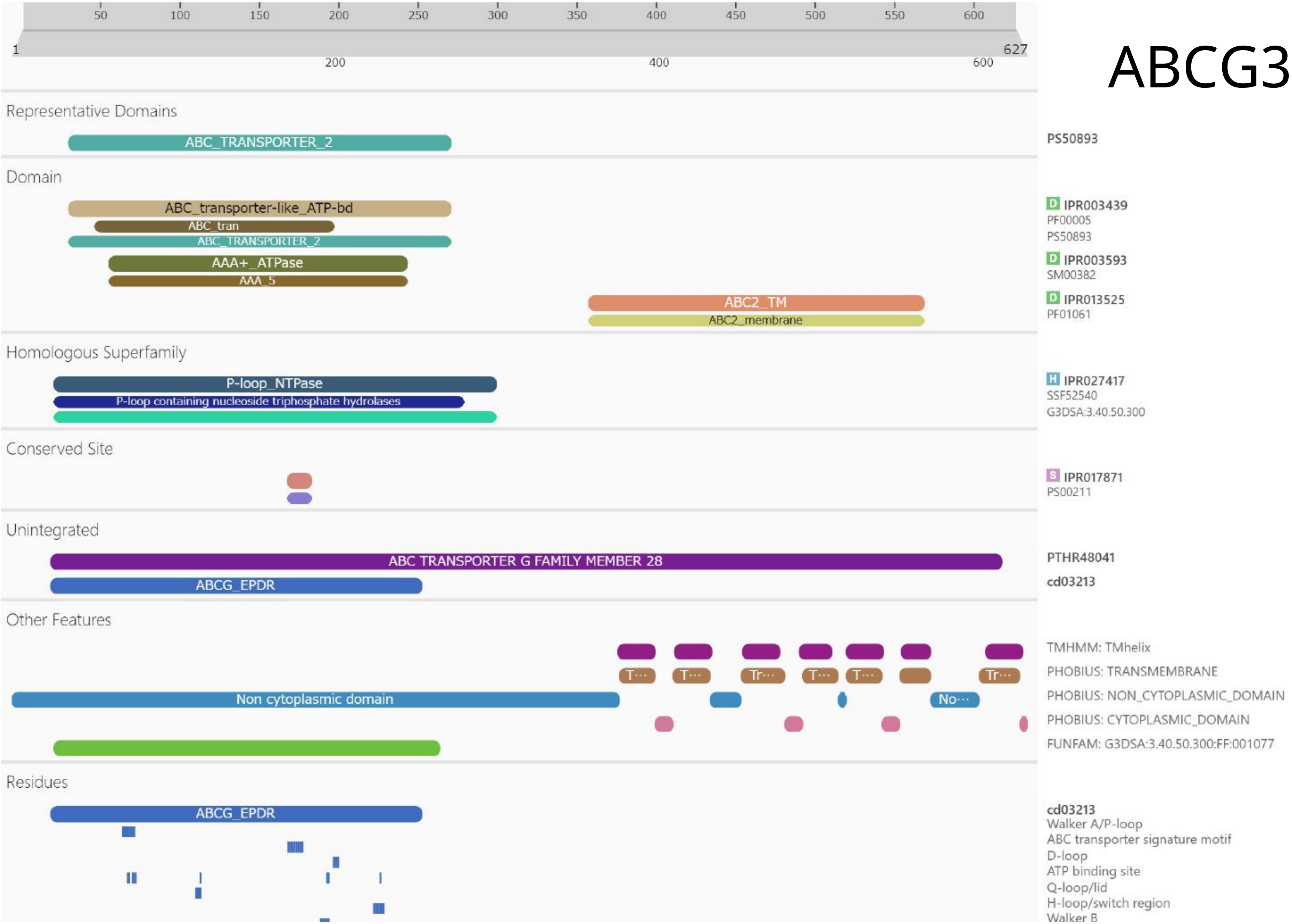
50 100 150 200 250 300 350 400 450 500 550 600
1
627
200 400 600
Representative Domains
ABC_TRANSPORTER_2
PS50893
Domain
ABC_transporter-like_ATP-bd
ABC_tran
ABC_TRANSPORTER_2
AAA+_ATPase
AAA_5
ABC2_TM
ABC2_membrane
D IPR003439
PF00005
PS50893
D IPR003593
SM00382
D IPR013525
PF01061
Homologous Superfamily
P-loop_NTPase
P-loop containing nucleoside triphosphate hydrolases
H IPR027417
SSF52540
G3DSA:3.40.50.300
Conserved Site
S IPR017871
PS00211
Unintegrated
ABC TRANSPORTER G FAMILY MEMBER 28
ABCG_EPDR
PTHR48041
cd03213
Other Features
Non cytoplasmic domain
TMHMM: TMhelix
PHOBIUS: TRANSMEMBRANE
PHOBIUS: NON_CYTOPLASMIC_DOMAIN
PHOBIUS: CYTOPLASMIC_DOMAIN
FUNFAM: G3DSA:3.40.50.300:FF:001077
Residues
ABCG_EPDR
cd03213
Walker A/P-loop
ABC transporter signature motif
D-loop
ATP binding site
Q-loop/lid
H-loop/switch region
Walker B

ABCG4

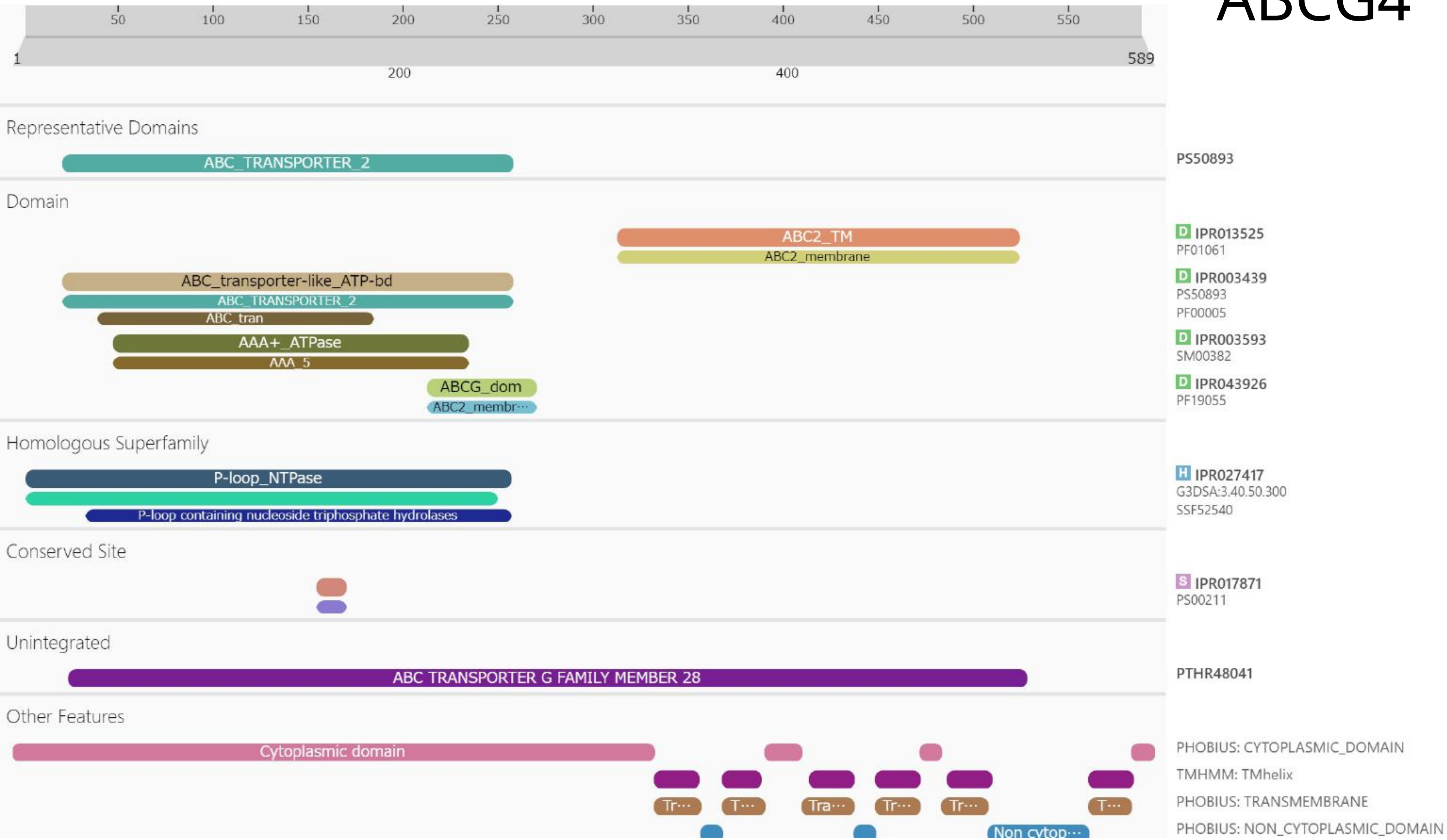
50
100
150
200
250
300
350
400
450
500
550
1
589
200
400
Representative Domains
ABC_TRANSPORTER_2
PS50893
Domain
ABC2_TM
ABC2_membrane
ABC_transporter-like_ATP-bd
ABC_TRANSPORTER_2
ABC_tran
AAA+_ATPase
AAA_5
ABCG_dom
ABC2_membr···
IPR013525
PF01061
IPR003439
PS50893
PF00005
IPR003593
SM00382
IPR043926
PF19055
Homologous Superfamily
P-loop_NTPase
P-loop containing nucleoside triphosphate hydrolases
IPR027417
G3DSA:3.40.50.300
SSF52540
Conserved Site
IPR017871
PS00211
Unintegrated
ABC TRANSPORTER G FAMILY MEMBER 28
PTHR48041
Other Features
Cytoplasmic domain
Non cytop···
PHOBIUS: CYTOPLASMIC_DOMAIN
TMHMM: TMhelix
PHOBIUS: TRANSMEMBRANE
PHOBIUS: NON_CYTOPLASMIC_DOMAIN

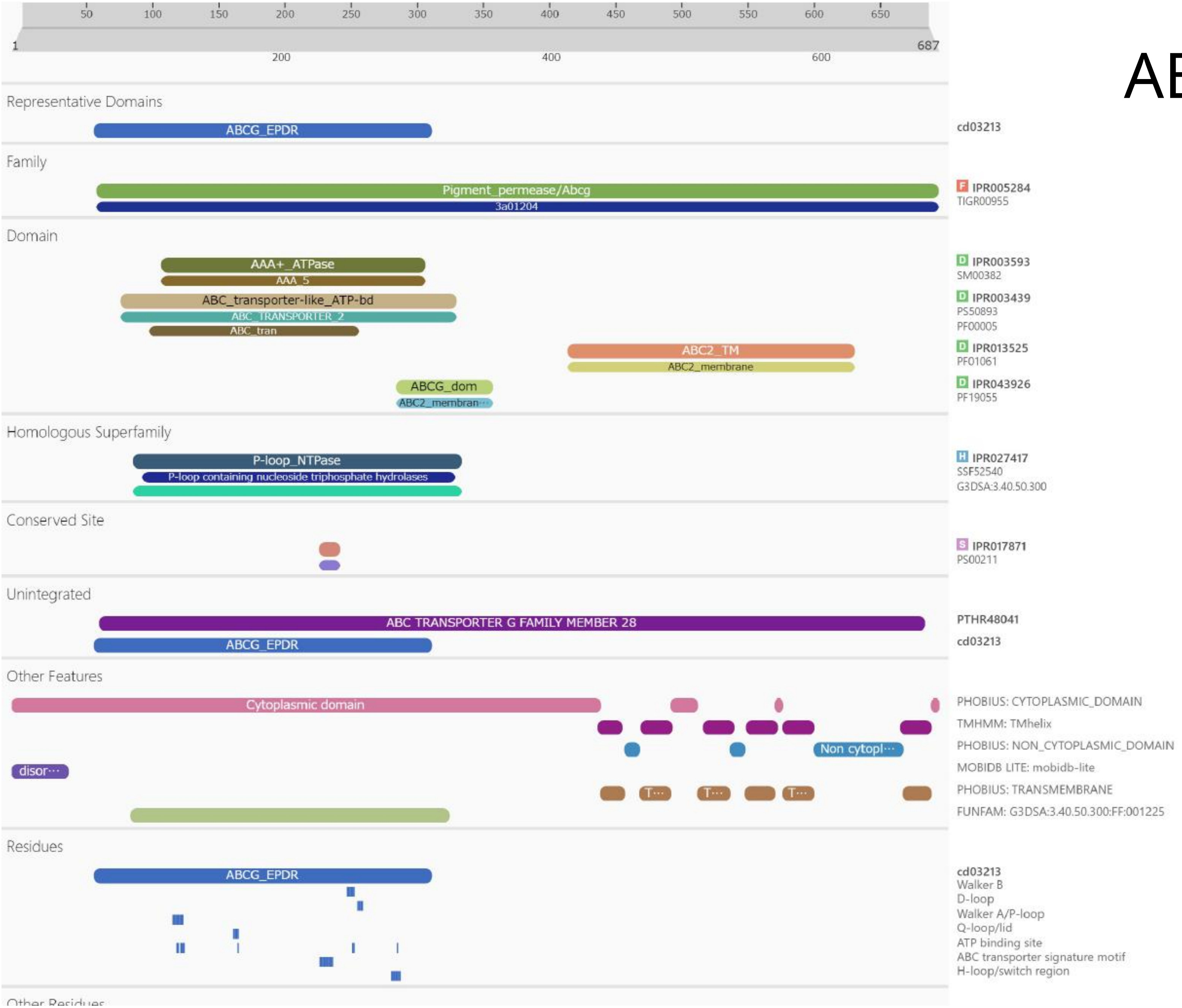

50 100 150 200 250 300 350 400 450 500 550 600 650
1 687
200 400 600
Representative Domains
ABCG_EPDR
cd03213
Family
Pigment_permease/Abcg
3a01204
IPR005284
TIGR00955
Domain
AAA+_ATPase
AAA_5
ABC_transporter-like_ATP-bd
ABC_TRANSPORTER_2
ABC_tran
ABC2_TM
ABC2_membrane
ABCG_dom
IPR003593
SM00382
IPR003439
PS50893
PF00005
IPR013525
PF01061
IPR043926
PF19055
Homologous Superfamily
P-loop_NTPase
P-loop containing nucleoside triphosphate hydrolases
IPR027417
SSF52540
G3DSA:3.40.50.300
Conserved Site
IPR017871
PS00211
Unintegrated
ABC TRANSPORTER G FAMILY MEMBER 28
ABCG_EPDR
PTHR48041
cd03213
Other Features
Cytoplasmic domain
Non cytopl···
disor···
PHOBIUS: CYTOPLASMIC_DOMAIN
TMHMM: TMhelix
PHOBIUS: NON_CYTOPLASMIC_DOMAIN
MOBIDB LITE: mobidb-lite
PHOBIUS: TRANSMEMBRANE
FUNFAM: G3DSA:3.40.50.300:FF:001225
Residues
ABCG_EPDR
cd03213
Walker B
D-loop
Walker A/P-loop
Q-loop/lid
ATP binding site
ABC transporter signature motif
H-loop/switch region

ABCG5

ABCG6

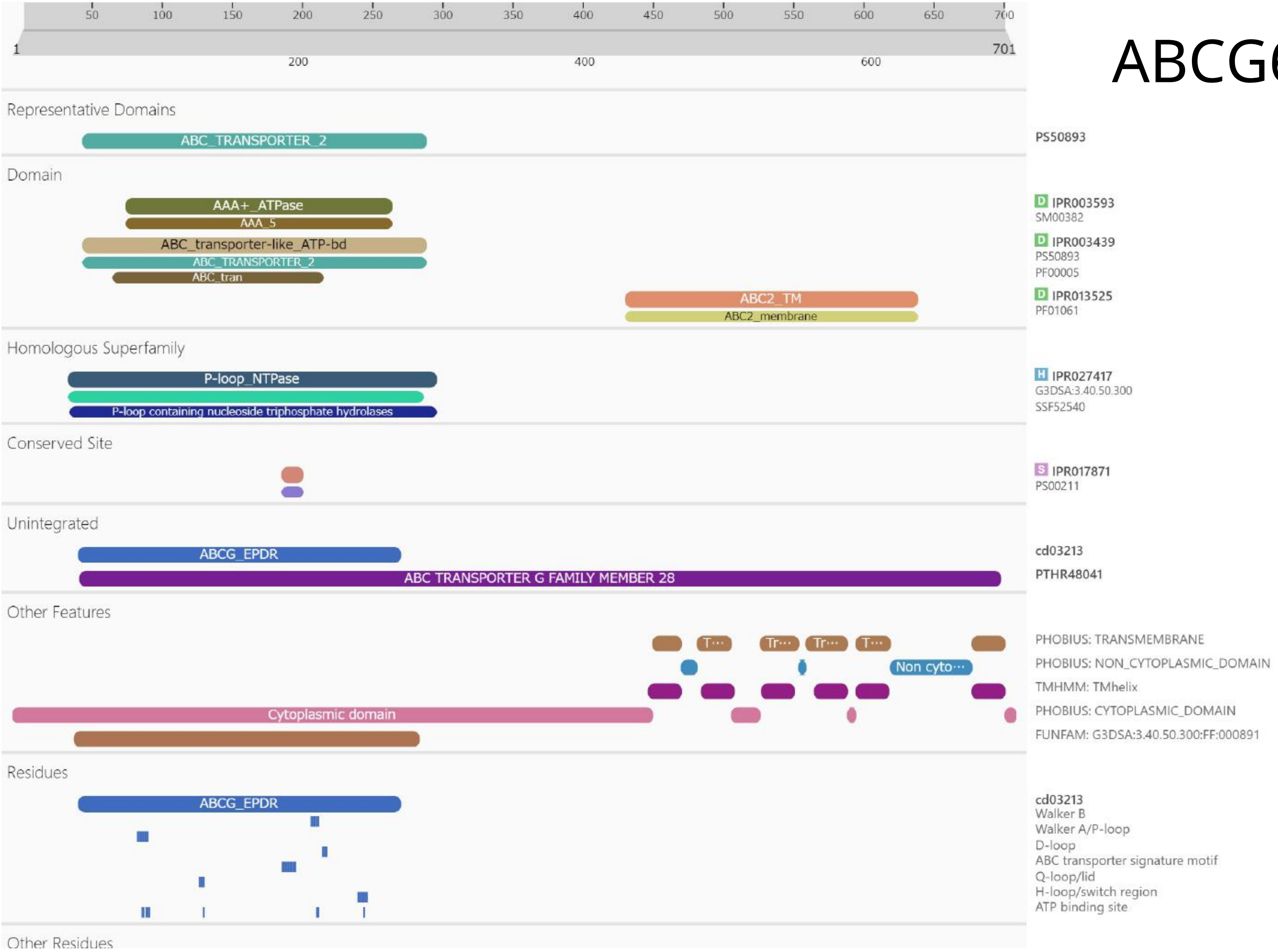
1
701
200
400
600
Representative Domains
ABC_TRANSPORTER_2
PS50893
Domain
AAA+_ATPase
AAA_5
ABC_transporter-like_ATP-bd
ABC_TRANSPORTER_2
ABC_tran
ABC2_TM
ABC2_membrane
IPR003593
SM00382
IPR003439
PS50893
PF00005
IPR013525
PF01061
Homologous Superfamily
P-loop_NTPase
P-loop containing nucleoside triphosphate hydrolases
IPR027417
G3DSA:3.40.50.300
SSF52540
Conserved Site
IPR017871
PS00211
Unintegrated
ABCG_EPDR
ABC TRANSPORTER G FAMILY MEMBER 28
cd03213
PTHR48041
Other Features
Non cyto…
Cytoplasmic domain
PHOBIUS: TRANSMEMBRANE
PHOBIUS: NON_CYTOPLASMIC_DOMAIN
TMHMM: TMhelix
PHOBIUS: CYTOPLASMIC_DOMAIN
FUNFAM: G3DSA:3.40.50.300:FF:000891
Residues
ABCG_EPDR
cd03213
Walker B
Walker A/P-loop
D-loop
ABC transporter signature motif
Q-loop/lid
H-loop/switch region
ATP binding site
Other Residues

ABCG7

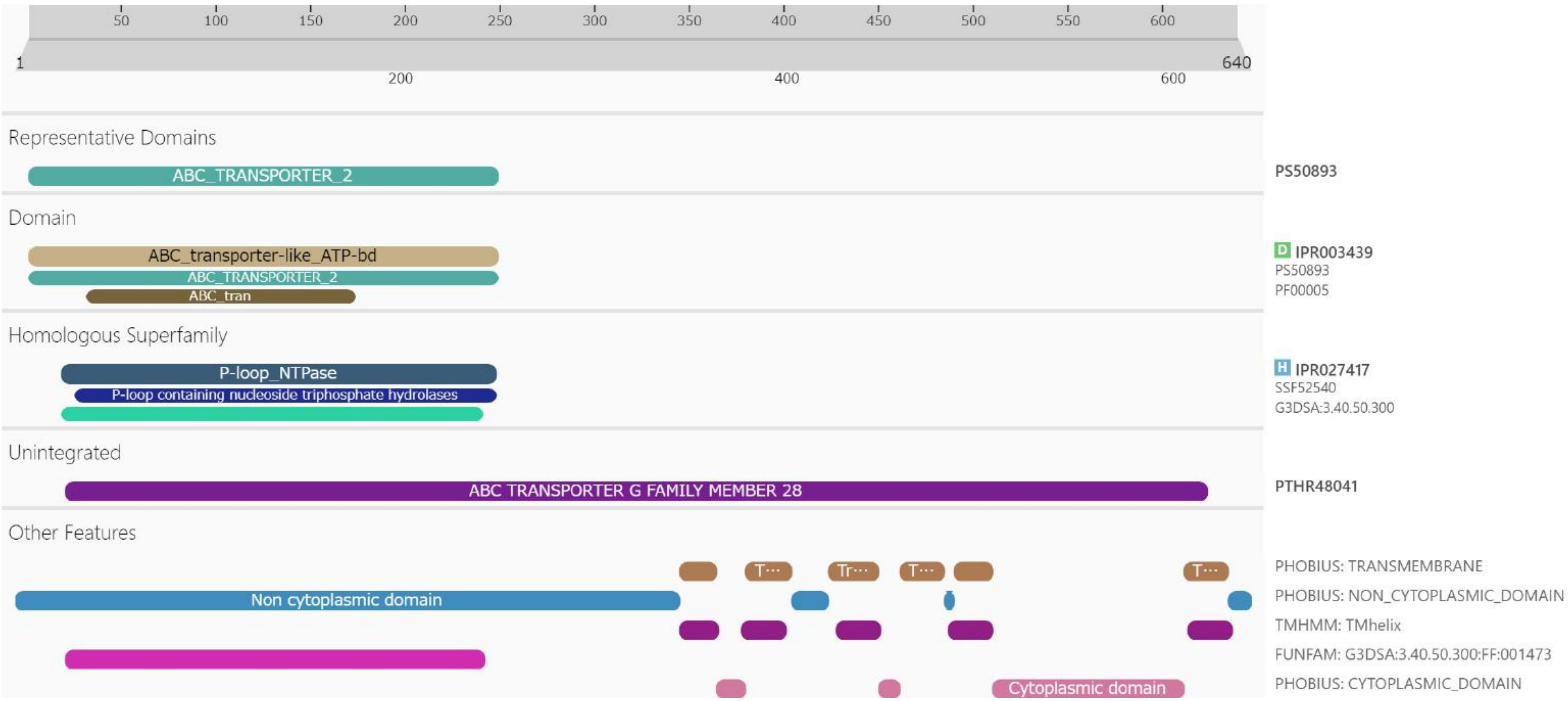

50
100
150
200
250
300
350
400
450
500
550
600
1
640
200
400
600
Representative Domains
ABC_TRANSPORTER_2
PS50893
Domain
ABC_transporter-like_ATP-bd
ABC_TRANSPORTER_2
ABC_tran
D IPR003439
PS50893
PF00005
Homologous Superfamily
P-loop_NTPase
P-loop containing nucleoside triphosphate hydrolases
H IPR027417
SSF52540
G3DSA:3.40.50.300
Unintegrated
ABC TRANSPORTER G FAMILY MEMBER 28
PTHR48041
Other Features
Non cytoplasmic domain
Cytoplasmic domain
PHOBIUS: TRANSMEMBRANE
PHOBIUS: NON_CYTOPLASMIC_DOMAIN
TMHMM: TMhelix
FUNFAM: G3DSA:3.40.50.300:FF:001473
PHOBIUS: CYTOPLASMIC_DOMAIN

ABCG8

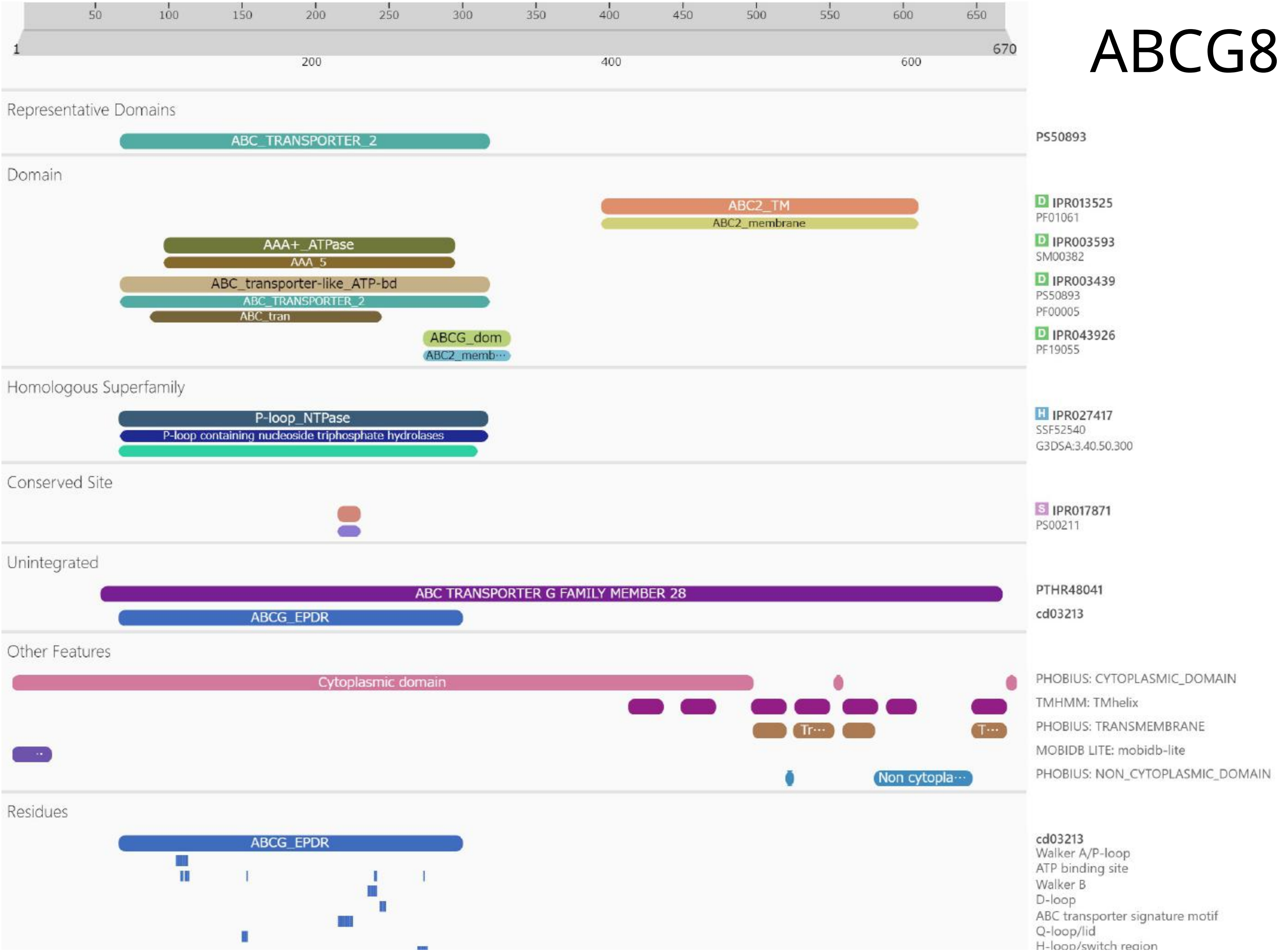

50
100
150
200
250
300
350
400
450
500
550
600
650
1
670
200
400
600
Representative Domains
ABC_TRANSPORTER_2
PS50893
Domain
ABC2_TM
ABC2_membrane
AAA+_ATPase
AAA_5
ABC_transporter-like_ATP-bd
ABC_TRANSPORTER_2
ABC_tran
ABCG_dom
ABC2_memb···
D IPR013525
PF01061
D IPR003593
SM00382
D IPR003439
PS50893
PF00005
D IPR043926
PF19055
Homologous Superfamily
P-loop_NTPase
P-loop containing nucleoside triphosphate hydrolases
H IPR027417
SSF52540
G3DSA:3.40.50.300
Conserved Site
S IPR017871
PS00211
Unintegrated
ABC TRANSPORTER G FAMILY MEMBER 28
ABCG_EPDR
PTHR48041
cd03213
Other Features
Cytoplasmic domain
Tr···
T···
Non cytopla···
PHOBIUS: CYTOPLASMIC_DOMAIN
TMHMM: TMhelix
PHOBIUS: TRANSMEMBRANE
MOBIDB LITE: mobidb-lite
PHOBIUS: NON_CYTOPLASMIC_DOMAIN
Residues
ABCG_EPDR
cd03213
Walker A/P-loop
ATP binding site
Walker B
D-loop
ABC transporter signature motif
Q-loop/lid
H-loop/switch region

ABCG9

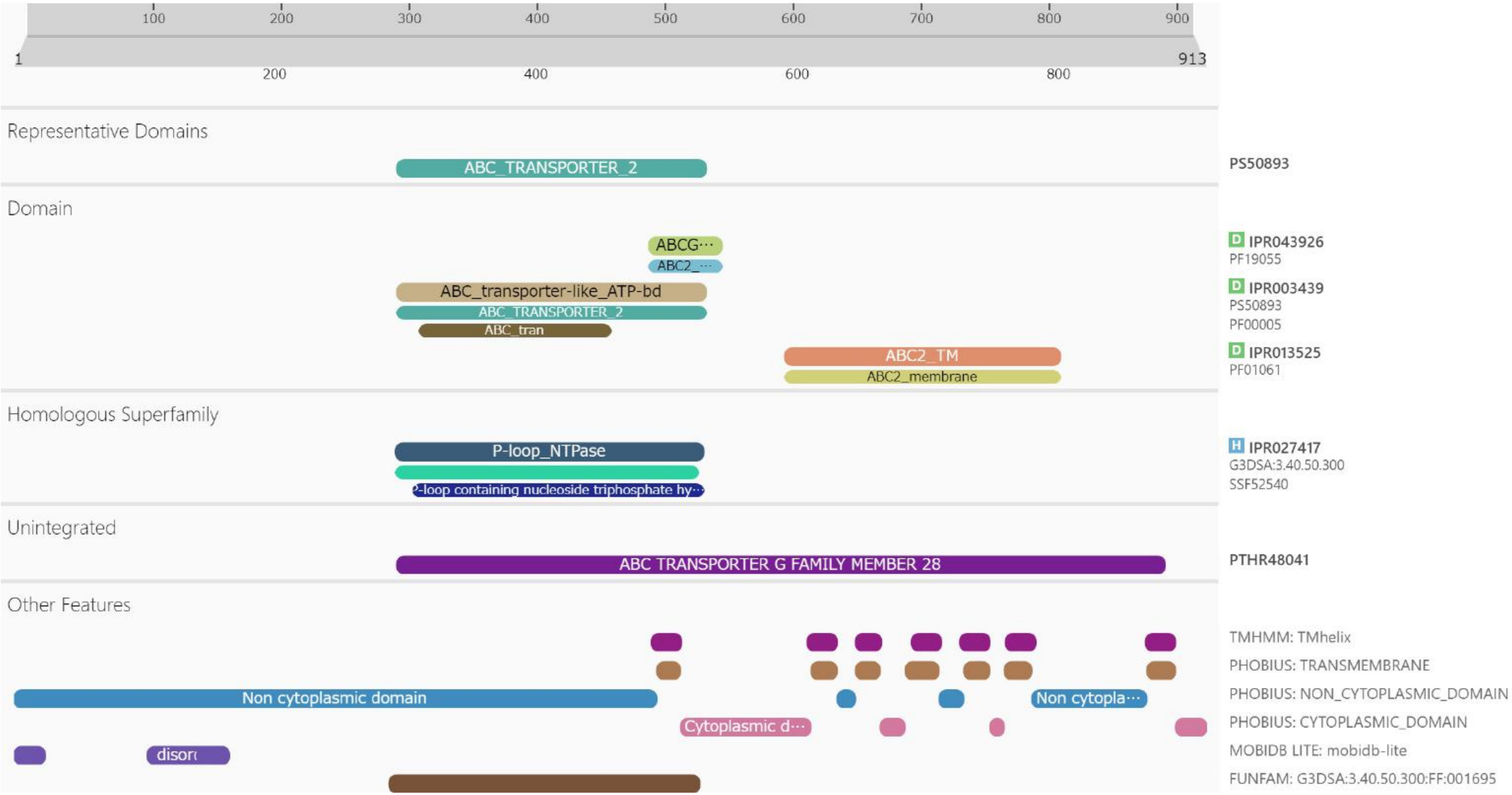
100
200
300
400
500
600
700
800
900
1
913
200
400
600
800
Representative Domains
ABC_TRANSPORTER_2
PS50893
Domain
ABCG···
ABC2_···
ABC_transporter-like_ATP-bd
ABC_TRANSPORTER_2
ABC_tran
ABC2_TM
ABC2_membrane
IPR043926
PF19055
IPR003439
PS50893
PF00005
IPR013525
PF01061
Homologous Superfamily
P-loop_NTPase
P-loop containing nucleoside triphosphate hy···
IPR027417
G3DSA:3.40.50.300
SSF52540
Unintegrated
ABC TRANSPORTER G FAMILY MEMBER 28
PTHR48041
Other Features
Non cytoplasmic domain
Non cytopla···
Cytoplasmic d···
TMHMM: TMhelix
PHOBIUS: TRANSMEMBRANE
PHOBIUS: NON_CYTOPLASMIC_DOMAIN
PHOBIUS: CYTOPLASMIC_DOMAIN
MOBIDB LITE: mobidb-lite
FUNFAM: G3DSA:3.40.50.300:FF:001695

ABCG10

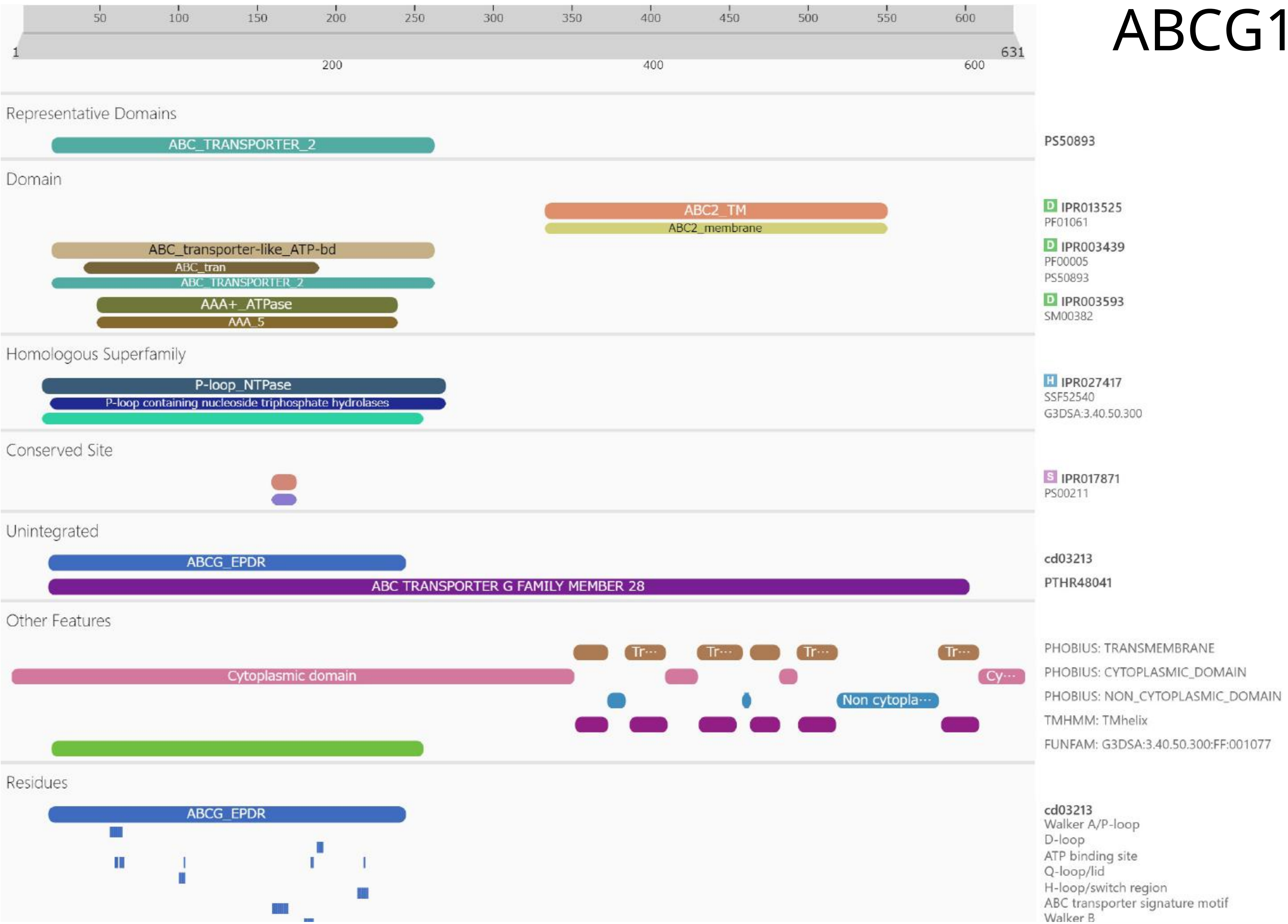

50 100 150 200 250 300 350 400 450 500 550 600
1 631
200 400 600
Representative Domains
ABC_TRANSPORTER_2
PS50893
Domain
ABC2_TM
ABC2_membrane
ABC_transporter-like_ATP-bd
ABC_tran
ABC_TRANSPORTER_2
AAA+_ATPase
AAA_5
IPR013525
PF01061
IPR003439
PF00005
PS50893
IPR003593
SM00382
Homologous Superfamily
P-loop_NTPase
P-loop containing nucleoside triphosphate hydrolases
IPR027417
SSF52540
G3DSA:3.40.50.300
Conserved Site
IPR017871
PS00211
Unintegrated
ABCG_EPDR
ABC TRANSPORTER G FAMILY MEMBER 28
cd03213
PTHR48041
Other Features
Cytoplasmic domain
Non cytopla···
PHOBIUS: TRANSMEMBRANE
PHOBIUS: CYTOPLASMIC_DOMAIN
PHOBIUS: NON_CYTOPLASMIC_DOMAIN
TMHMM: TMhelix
FUNFAM: G3DSA:3.40.50.300:FF:001077
Residues
ABCG_EPDR
cd03213
Walker A/P-loop
D-loop
ATP binding site
Q-loop/lid
H-loop/switch region
ABC transporter signature motif
Walker B

ABCG11

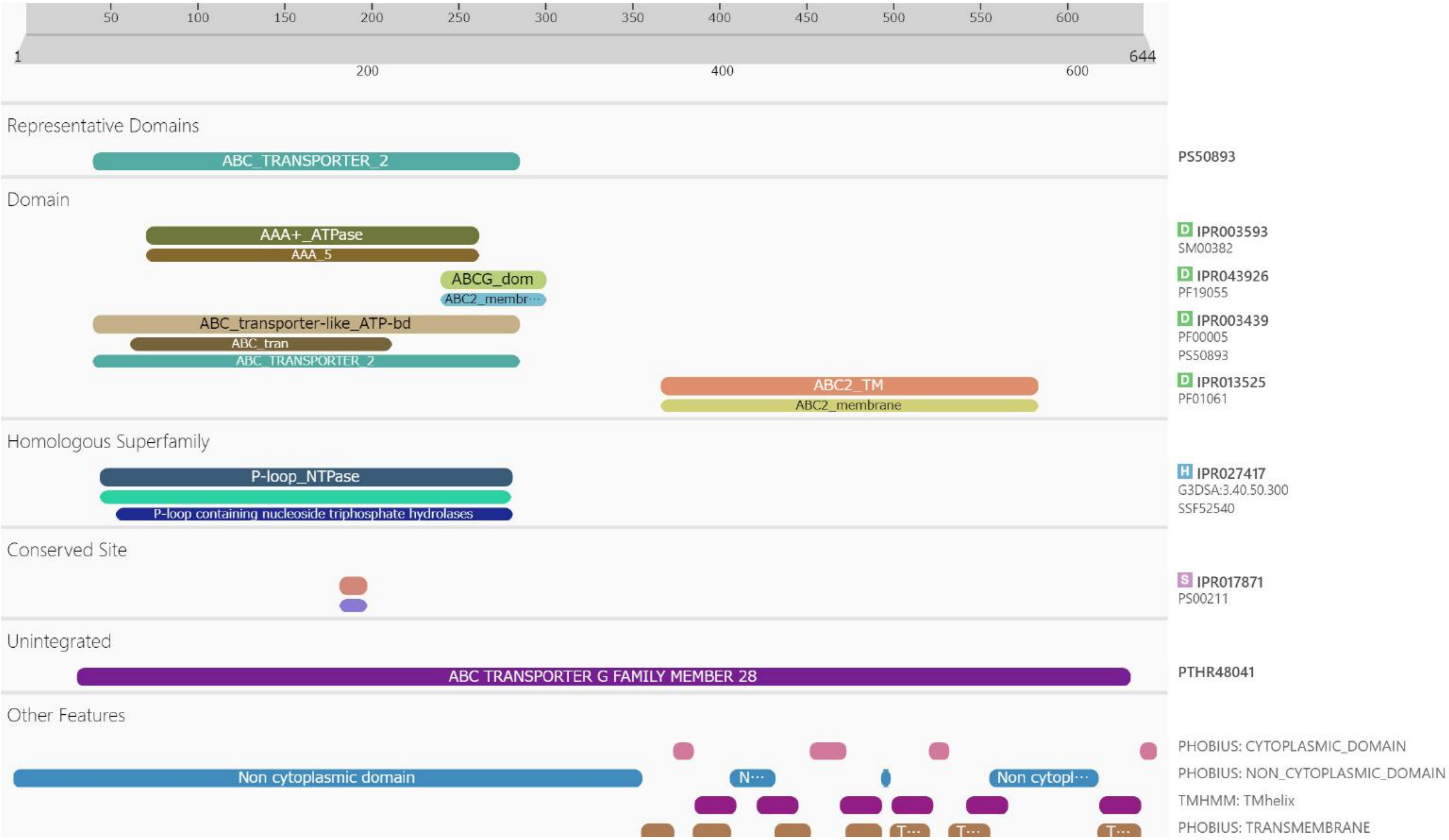
50 100 150 200 250 300 350 400 450 500 550 600
1
644
200 400 600
Representative Domains
ABC_TRANSPORTER_2
PS50893
Domain
AAA+_ATPase
AAA_5
ABCG_dom
ABC2_membr…
ABC_transporter-like_ATP-bd
ABC_tran
ABC_TRANSPORTER_2
ABC2_TM
ABC2_membrane
IPR003593
SM00382
IPR043926
PF19055
IPR003439
PF00005
PS50893
IPR013525
PF01061
Homologous Superfamily
P-loop_NTPase
P-loop containing nucleoside triphosphate hydrolases
IPR027417
G3DSA:3.40.50.300
SSF52540
Conserved Site
IPR017871
PS00211
Unintegrated
ABC TRANSPORTER G FAMILY MEMBER 28
PTHR48041
Other Features
Non cytoplasmic domain
N…
Non cytopl…
T…
T…
T…
PHOBIUS: CYTOPLASMIC_DOMAIN
PHOBIUS: NON_CYTOPLASMIC_DOMAIN
TMHMM: TMhelix
PHOBIUS: TRANSMEMBRANE

ABCG12

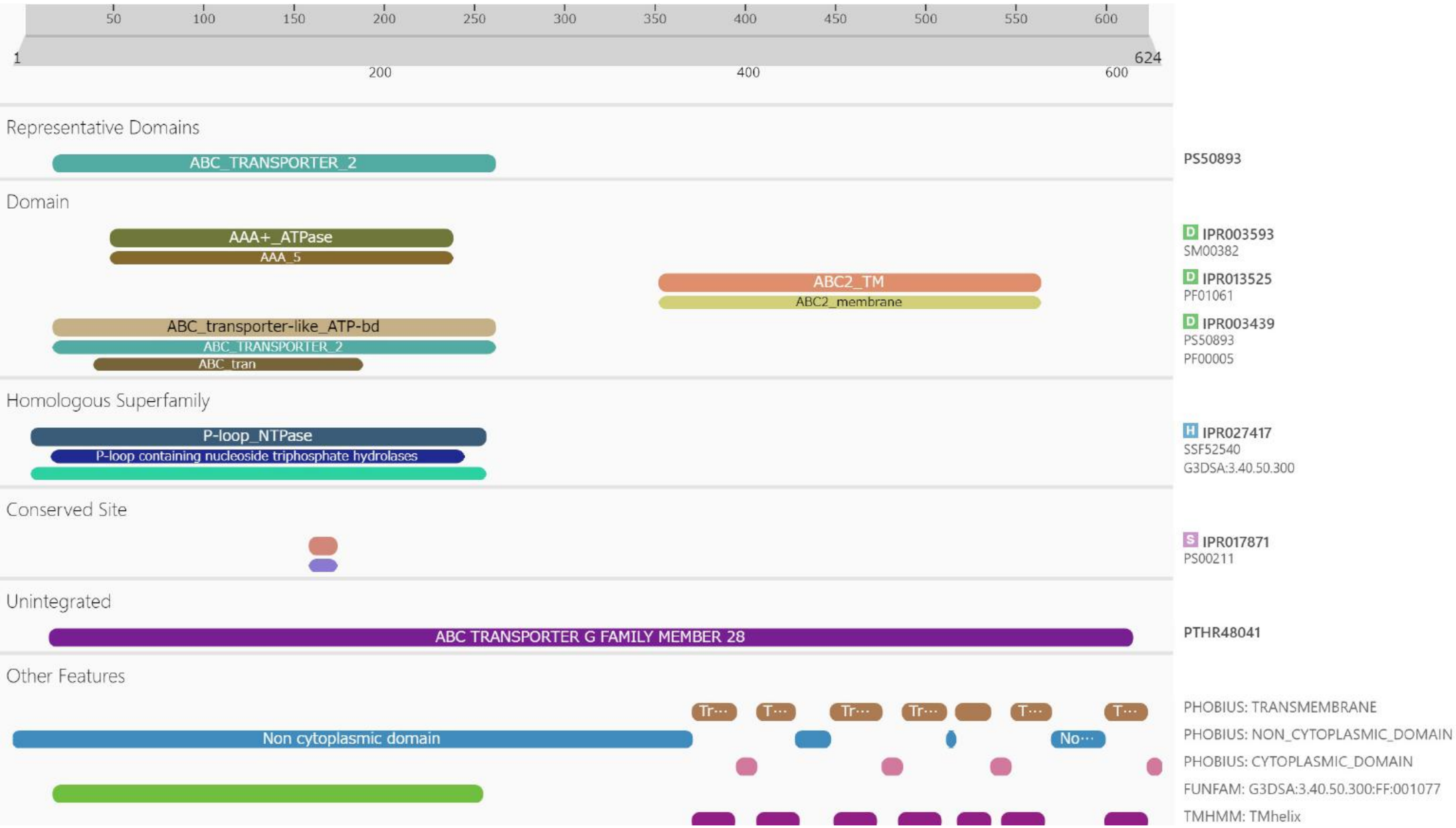
50 100 150 200 250 300 350 400 450 500 550 600
1
624
200 400 600
Representative Domains
ABC_TRANSPORTER_2
PS50893
Domain
AAA+_ATPase
AAA_5
ABC2_TM
ABC2_membrane
ABC_transporter-like_ATP-bd
ABC_TRANSPORTER_2
ABC_tran
D IPR003593
SM00382
D IPR013525
PF01061
D IPR003439
PS50893
PF00005
Homologous Superfamily
P-loop_NTPase
P-loop containing nucleoside triphosphate hydrolases
H IPR027417
SSF52540
G3DSA:3.40.50.300
Conserved Site
S IPR017871
PS00211
Unintegrated
ABC TRANSPORTER G FAMILY MEMBER 28
PTHR48041
Other Features
Non cytoplasmic domain
PHOBIUS: TRANSMEMBRANE
PHOBIUS: NON_CYTOPLASMIC_DOMAIN
PHOBIUS: CYTOPLASMIC_DOMAIN
FUNFAM: G3DSA:3.40.50.300:FF:001077
TMHMM: TMhelix

ABCG4-like1

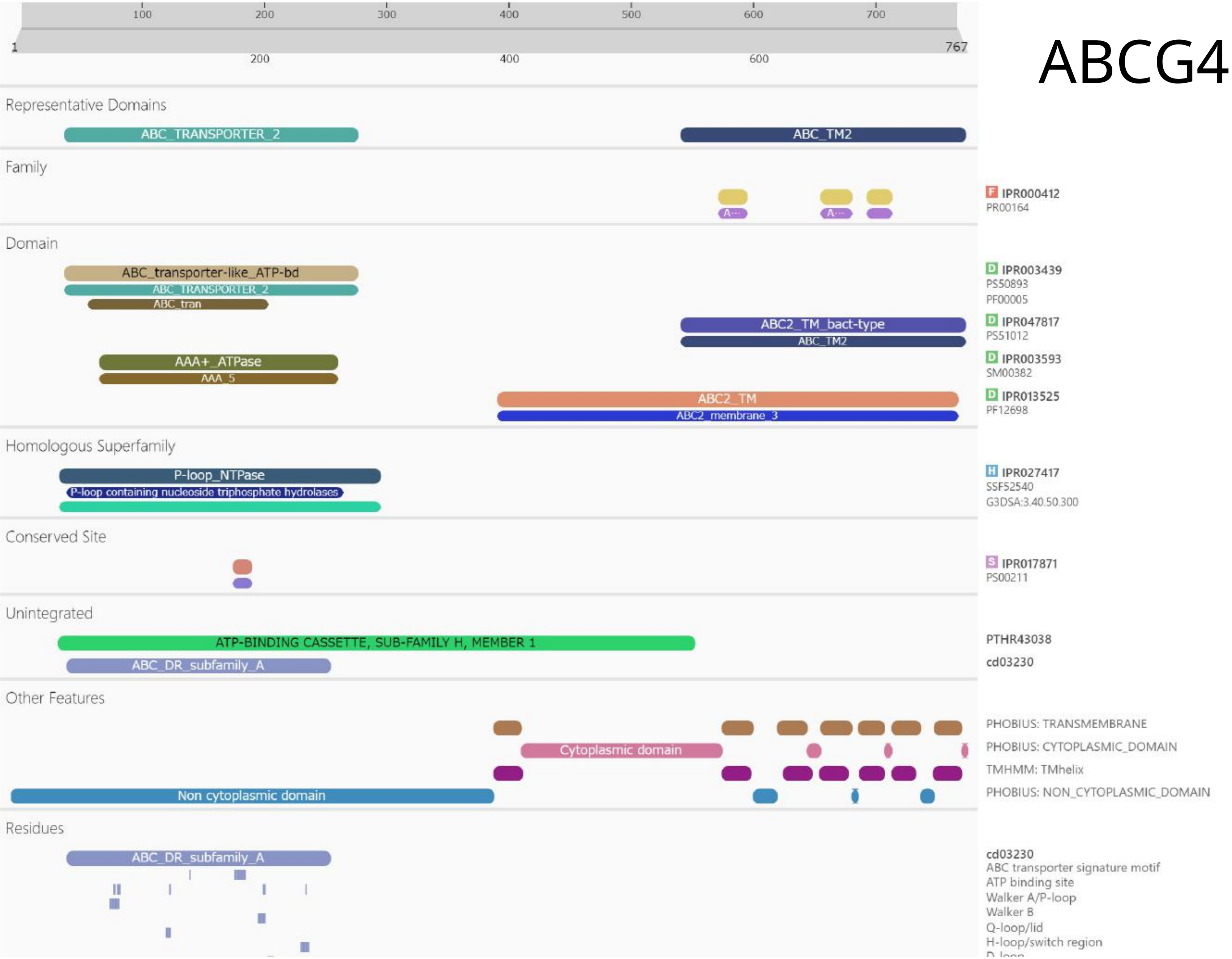

1
767
Representative Domains
ABC_TRANSPORTER_2
ABC_TM2
Family
IPR000412
PR00164
Domain
ABC_transporter-like_ATP-bd
ABC_TRANSPORTER_2
ABC_tran
ABC2_TM_bact-type
ABC_TM2
AAA+_ATPase
AAA_5
ABC2_TM
ABC2_membrane_3
IPR003439
PS50893
PF00005
IPR047817
PS51012
IPR003593
SM00382
IPR013525
PF12698
Homologous Superfamily
P-loop_NTPase
P-loop containing nucleoside triphosphate hydrolases
IPR027417
SSF52540
G3DSA:3.40.50.300
Conserved Site
IPR017871
PS00211
Unintegrated
ATP-BINDING CASSETTE, SUB-FAMILY H, MEMBER 1
ABC_DR_subfamily_A
PTHR43038
cd03230
Other Features
Cytoplasmic domain
Non cytoplasmic domain
PHOBIUS: TRANSMEMBRANE
PHOBIUS: CYTOPLASMIC_DOMAIN
TMHMM: TMhelix
PHOBIUS: NON_CYTOPLASMIC_DOMAIN
Residues
ABC_DR_subfamily_A
cd03230
ABC transporter signature motif
ATP binding site
Walker A/P-loop
Walker B
Q-loop/lid
H-loop/switch region

ABCG23

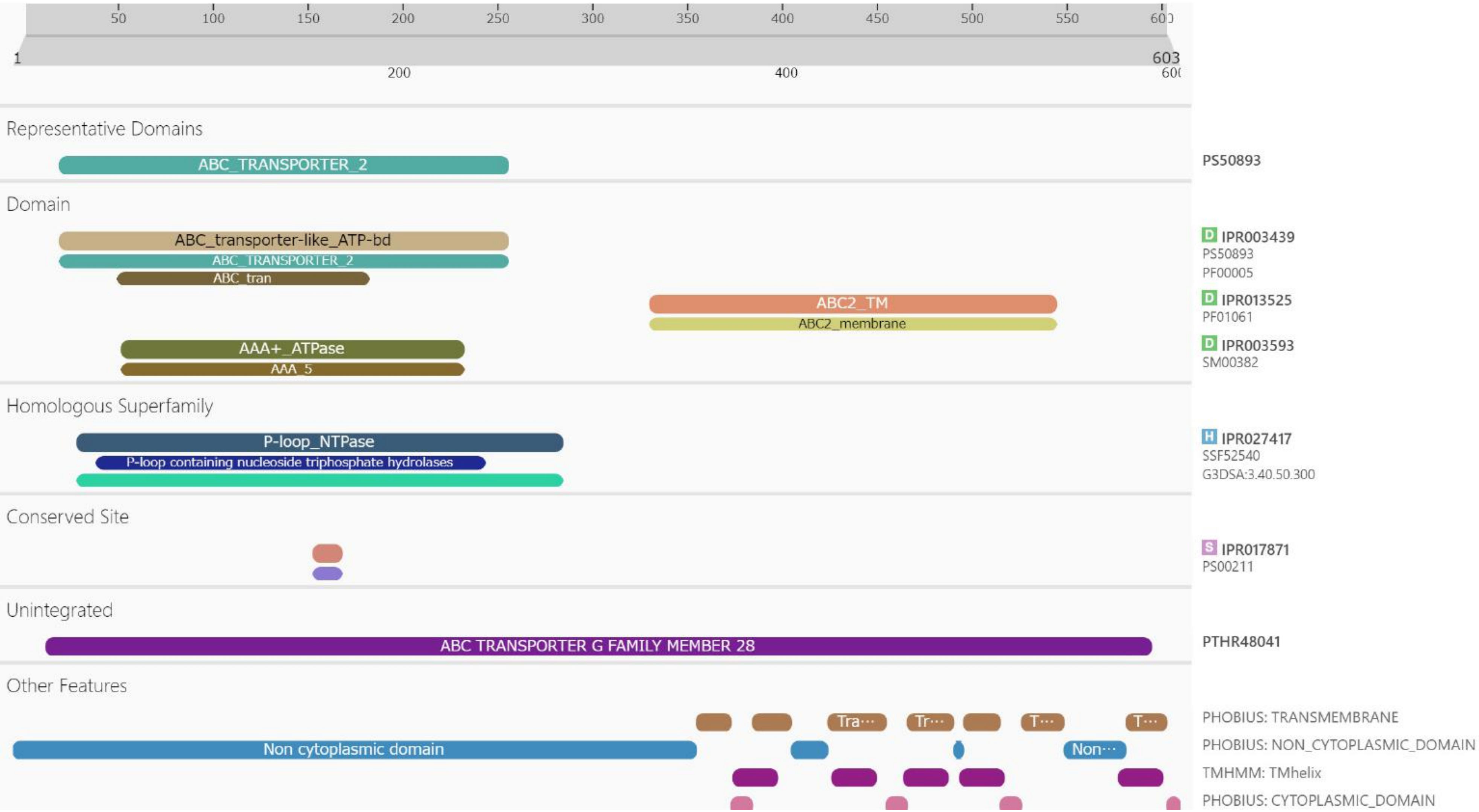

50 100 150 200 250 300 350 400 450 500 550 600
1
603
200
400
Representative Domains
ABC_TRANSPORTER_2
PS50893
Domain
ABC_transporter-like_ATP-bd
ABC_TRANSPORTER_2
ABC_tran
ABC2_TM
ABC2_membrane
AAA+_ATPase
AAA_5
D IPR003439
PS50893
PF00005
D IPR013525
PF01061
D IPR003593
SM00382
Homologous Superfamily
P-loop_NTPase
P-loop containing nucleoside triphosphate hydrolases
H IPR027417
SSF52540
G3DSA:3.40.50.300
Conserved Site
S IPR017871
PS00211
Unintegrated
ABC TRANSPORTER G FAMILY MEMBER 28
PTHR48041
Other Features
Non cytoplasmic domain
PHOBIUS: TRANSMEMBRANE
PHOBIUS: NON_CYTOPLASMIC_DOMAIN
TMHMM: TMhelix
PHOBIUS: CYTOPLASMIC_DOMAIN

ABCG4-like2

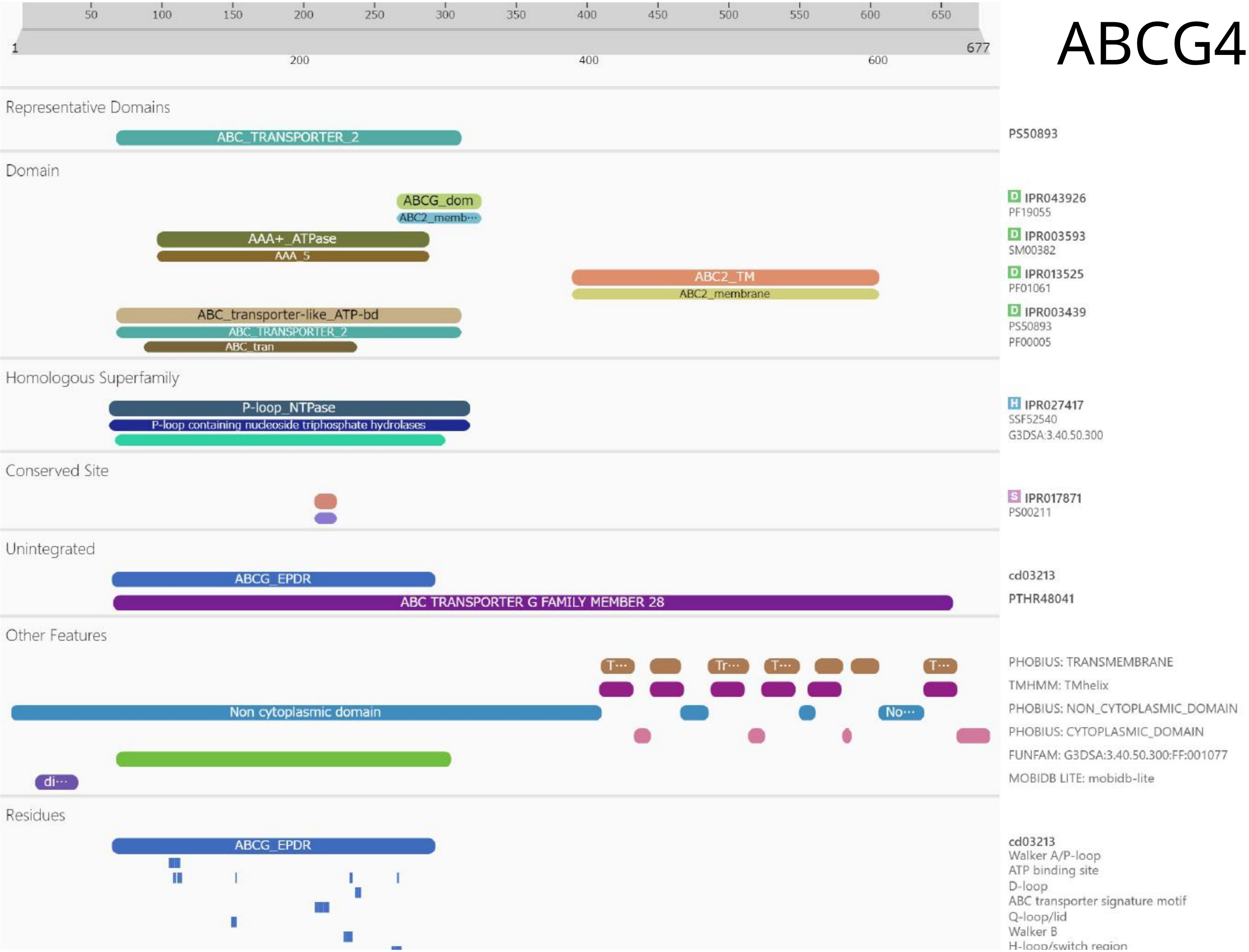

50 100 150 200 250 300 350 400 450 500 550 600 650
1 677
200 400 600
Representative Domains
ABC_TRANSPORTER_2
PS50893
Domain
ABCG_dom
ABC2_memb···
AAA+_ATPase
AAA_5
ABC2_TM
ABC2_membrane
ABC_transporter-like_ATP-bd
ABC_TRANSPORTER_2
ABC_tran
IPR043926
PF19055
IPR003593
SM00382
IPR013525
PF01061
IPR003439
PS50893
PF00005
Homologous Superfamily
P-loop_NTPase
P-loop containing nucleoside triphosphate hydrolases
IPR027417
SSF52540
G3DSA:3.40.50.300
Conserved Site
IPR017871
PS00211
Unintegrated
ABCG_EPDR
ABC TRANSPORTER G FAMILY MEMBER 28
cd03213
PTHR48041
Other Features
Non cytoplasmic domain
PHOBIUS: TRANSMEMBRANE
TMHMM: TMhelix
PHOBIUS: NON_CYTOPLASMIC_DOMAIN
PHOBIUS: CYTOPLASMIC_DOMAIN
FUNFAM: G3DSA:3.40.50.300:FF:001077
MOBIDB LITE: mobidb-lite
Residues
ABCG_EPDR
cd03213
Walker A/P-loop
ATP binding site
D-loop
ABC transporter signature motif
Q-loop/lid
Walker B
H-loop/switch region

ABCG1-like1

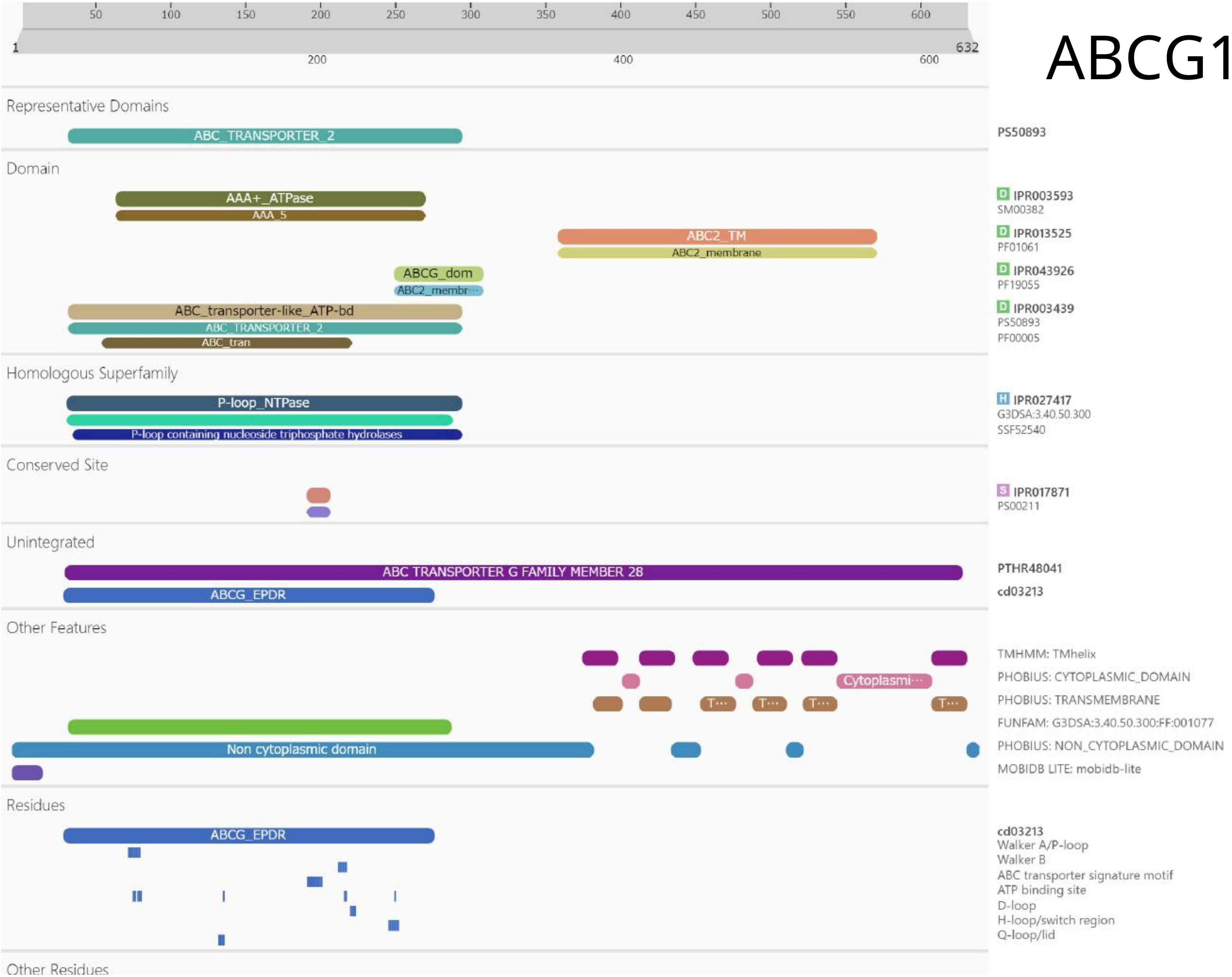
50 100 150 200 250 300 350 400 450 500 550 600
1
632
200 400 600
Representative Domains
ABC_TRANSPORTER_2
PS50893
Domain
AAA+_ATPase
AAA_5
ABC2_TM
ABC2_membrane
ABCG_dom
ABC2_membr…
ABC_transporter-like_ATP-bd
ABC_TRANSPORTER_2
ABC_tran
IPR003593
SM00382
IPR013525
PF01061
IPR043926
PF19055
IPR003439
PS50893
PF00005
Homologous Superfamily
P-loop_NTPase
P-loop containing nucleoside triphosphate hydrolases
IPR027417
G3DSA:3.40.50.300
SSF52540
Conserved Site
IPR017871
PS00211
Unintegrated
ABC TRANSPORTER G FAMILY MEMBER 28
ABCG_EPDR
PTHR48041
cd03213
Other Features
Cytoplasmi…
T…
Non cytoplasmic domain
TMHMM: TMhelix
PHOBIUS: CYTOPLASMIC_DOMAIN
PHOBIUS: TRANSMEMBRANE
FUNFAM: G3DSA:3.40.50.300:FF:001077
PHOBIUS: NON_CYTOPLASMIC_DOMAIN
MOBIDB LITE: mobidb-lite
Residues
ABCG_EPDR
cd03213
Walker A/P-loop
Walker B
ABC transporter signature motif
ATP binding site
D-loop
H-loop/switch region
Q-loop/lid
Other Residues

ABCG4-like3

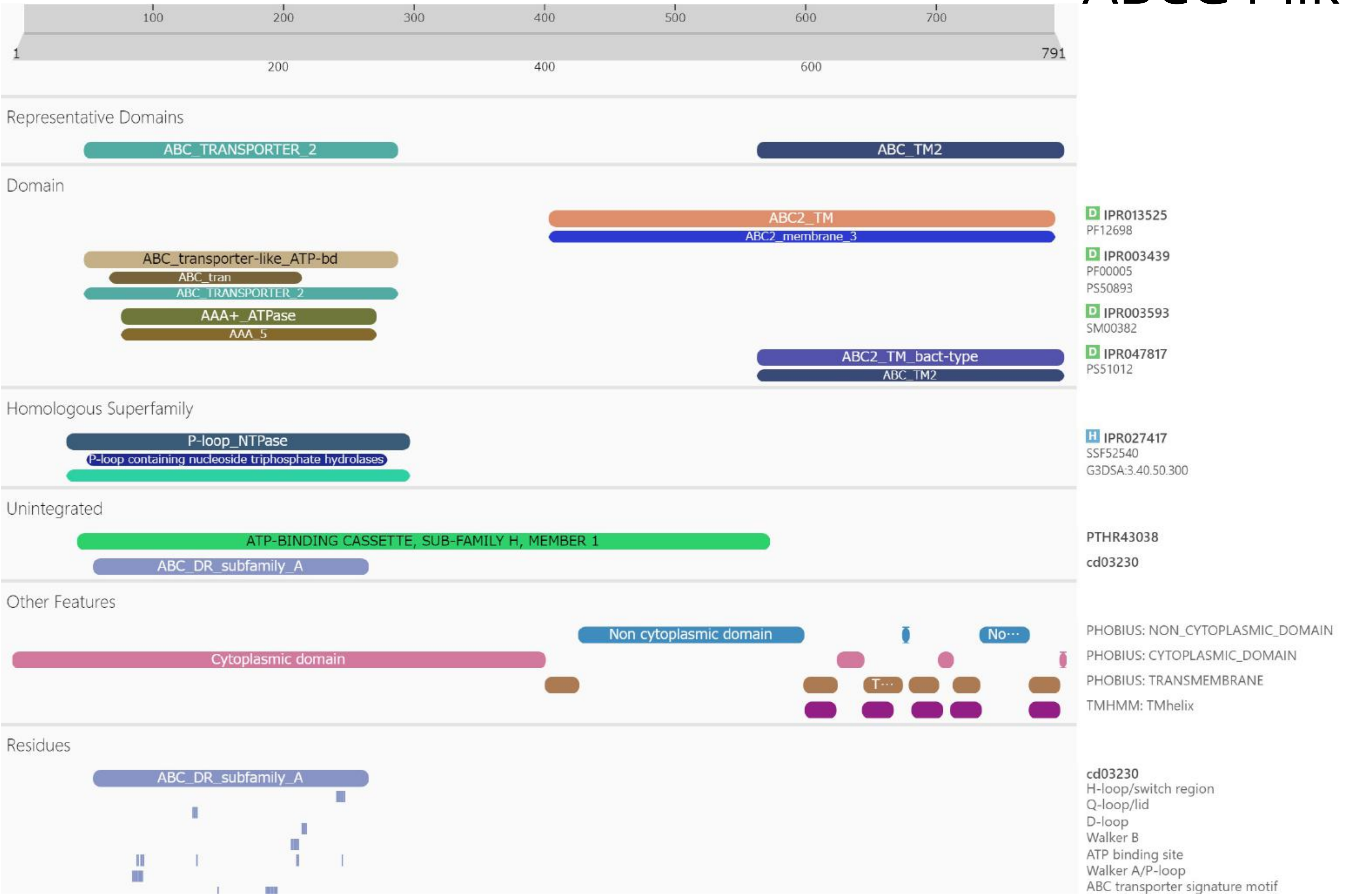

100
200
300
400
500
600
700
1
791
200
400
600
Representative Domains
ABC_TRANSPORTER_2
ABC_TM2
Domain
ABC2_TM
ABC2_membrane_3
ABC_transporter-like_ATP-bd
ABC_tran
ABC_TRANSPORTER_2
AAA+_ATPase
AAA_5
ABC2_TM_bact-type
ABC_TM2
IPR013525
PF12698
IPR003439
PF00005
PS50893
IPR003593
SM00382
IPR047817
PS51012
Homologous Superfamily
P-loop_NTPase
P-loop containing nucleoside triphosphate hydrolases
IPR027417
SSF52540
G3DSA:3.40.50.300
Unintegrated
ATP-BINDING CASSETTE, SUB-FAMILY H, MEMBER 1
ABC_DR_subfamily_A
PTHR43038
cd03230
Other Features
Non cytoplasmic domain
No···
Cytoplasmic domain
T···
PHOBIUS: NON_CYTOPLASMIC_DOMAIN
PHOBIUS: CYTOPLASMIC_DOMAIN
PHOBIUS: TRANSMEMBRANE
TMHMM: TMhelix
Residues
ABC_DR_subfamily_A
cd03230
H-loop/switch region
Q-loop/lid
D-loop
Walker B
ATP binding site
Walker A/P-loop
ABC transporter signature motif

ABCH1

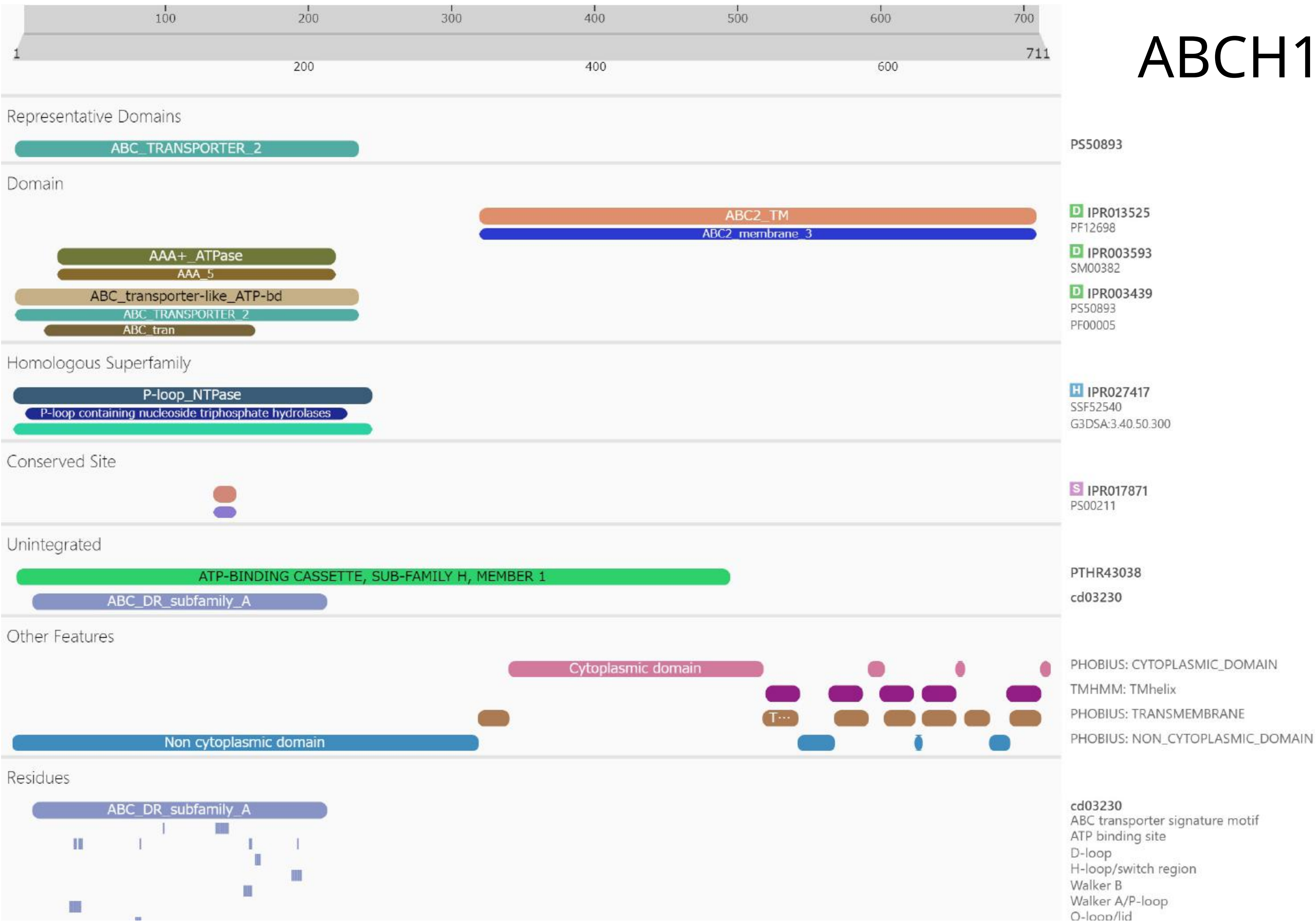

100
200
300
400
500
600
700
1
711
200
400
600
Representative Domains
ABC_TRANSPORTER_2
PS50893
Domain
ABC2_TM
ABC2_membrane_3
AAA+_ATPase
AAA_5
ABC_transporter-like_ATP-bd
ABC_TRANSPORTER_2
ABC_tran
D IPR013525
PF12698
D IPR003593
SM00382
D IPR003439
PS50893
PF00005
Homologous Superfamily
P-loop_NTPase
P-loop containing nucleoside triphosphate hydrolases
H IPR027417
SSF52540
G3DSA:3.40.50.300
Conserved Site
S IPR017871
PS00211
Unintegrated
ATP-BINDING CASSETTE, SUB-FAMILY H, MEMBER 1
ABC_DR_subfamily_A
PTHR43038
cd03230
Other Features
Cytoplasmic domain
T…
Non cytoplasmic domain
PHOBIUS: CYTOPLASMIC_DOMAIN
TMHMM: TMhelix
PHOBIUS: TRANSMEMBRANE
PHOBIUS: NON_CYTOPLASMIC_DOMAIN
Residues
ABC_DR_subfamily_A
cd03230
ABC transporter signature motif
ATP binding site
D-loop
H-loop/switch region
Walker B
Walker A/P-loop
Q-loop/lid

ABCH2

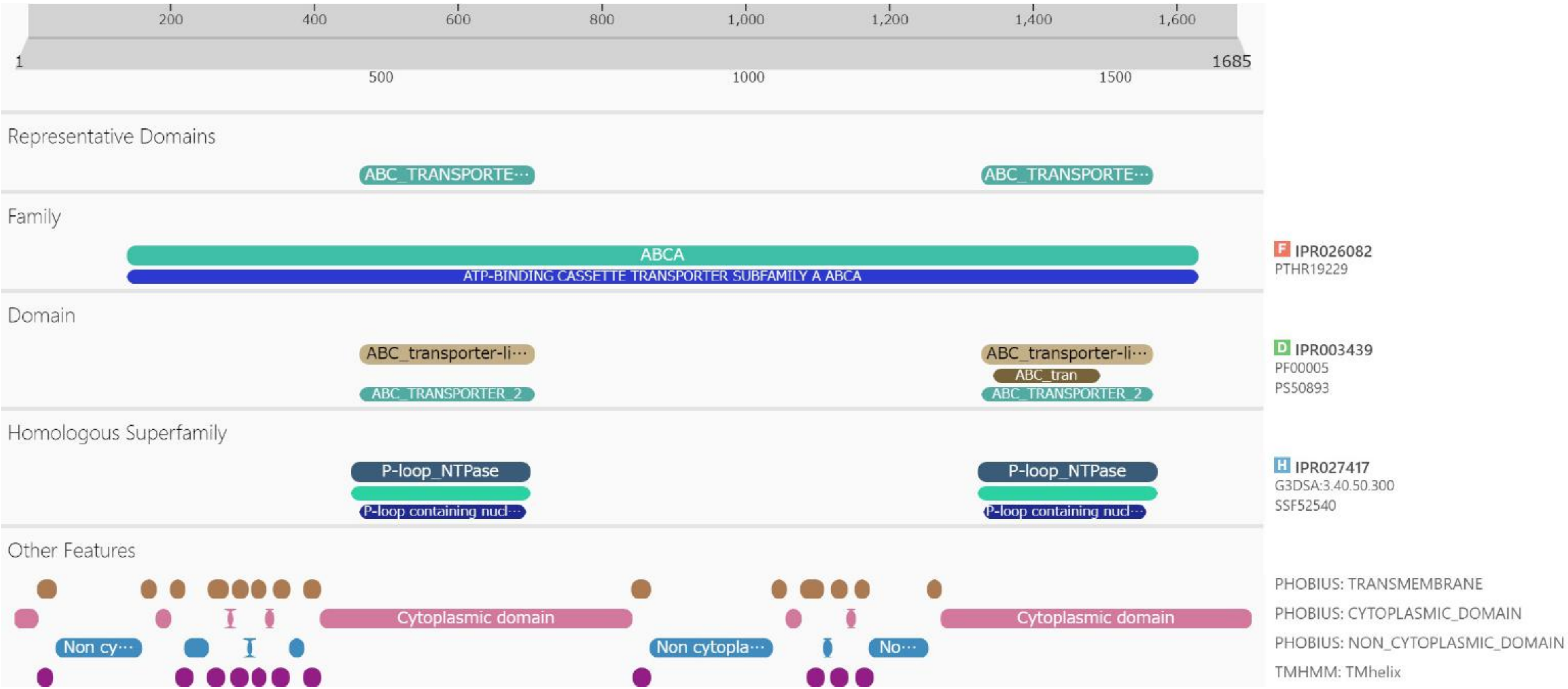
200
400
600
800
1,000
1,200
1,400
1,600
1
1685
500
1000
1500
Representative Domains
ABC_TRANSPORTE···
ABC_TRANSPORTE···
Family
ABCA
ATP-BINDING CASSETTE TRANSPORTER SUBFAMILY A ABCA
IPR026082
PTHR19229
Domain
ABC_transporter-li···
ABC_TRANSPORTER_2
ABC_transporter-li···
ABC_tran
ABC_TRANSPORTER_2
IPR003439
PF00005
PS50893
Homologous Superfamily
P-loop_NTPase
P-loop containing nucl···
P-loop_NTPase
P-loop containing nucl···
IPR027417
G3DSA:3.40.50.300
SSF52540
Other Features
Cytoplasmic domain
Cytoplasmic domain
Non cy···
Non cytopla···
No···
PHOBIUS: TRANSMEMBRANE
PHOBIUS: CYTOPLASMIC_DOMAIN
PHOBIUS: NON_CYTOPLASMIC_DOMAIN
TMHMM: TMhelix
