# Supplementary material for: Cry Toxins Use Multiple ATP-Binding Cassette Transporter Subfamily C Members as Low-Efficiency Receptors in Bombyx mori
Source: Biomolecules. 2024 Feb 23;14(3):271. doi: 10.3390/biom14030271 (PMC10968512; doi:10.3390/biom14030271)
Supplement: Supplementary file 1 [file biomolecules-14-00271-s001.zip › supplymentary_figs_revise2.pdf]

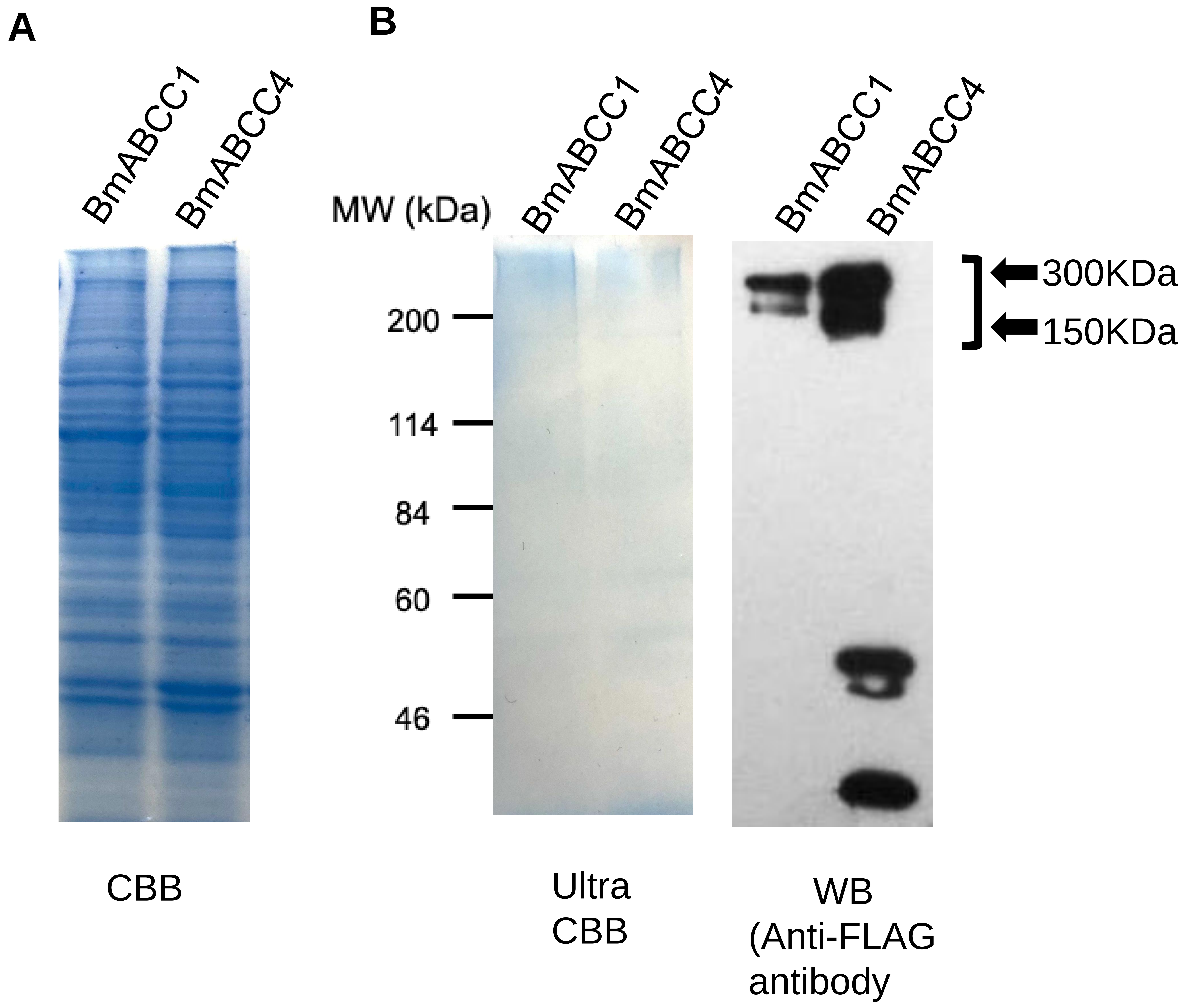

Supplementary Figure 1.

A

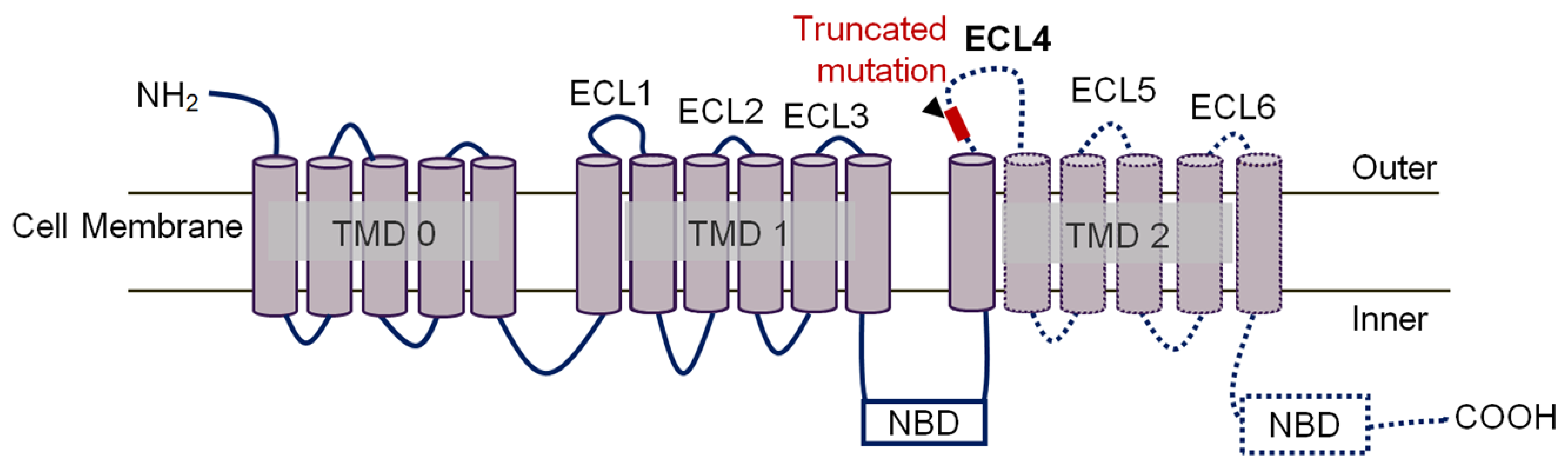

B

Wild   ATTCTGATGAATCTGATCCTGCAAGTGTTCCAAGTCGGCTCCAACTACTGGCTGGCGGAG
Ile Leu Met Asn Leu Ile Leu Gln Val Phe Gln Val Gly Ser Asn Tyr Trp Leu Ala Glu

C1T09   ATTCTGATGAAT---------------------------- GTTCCAAGTCGGCTCCAACTACTGGCTGGCGGAG (-14nt)

C

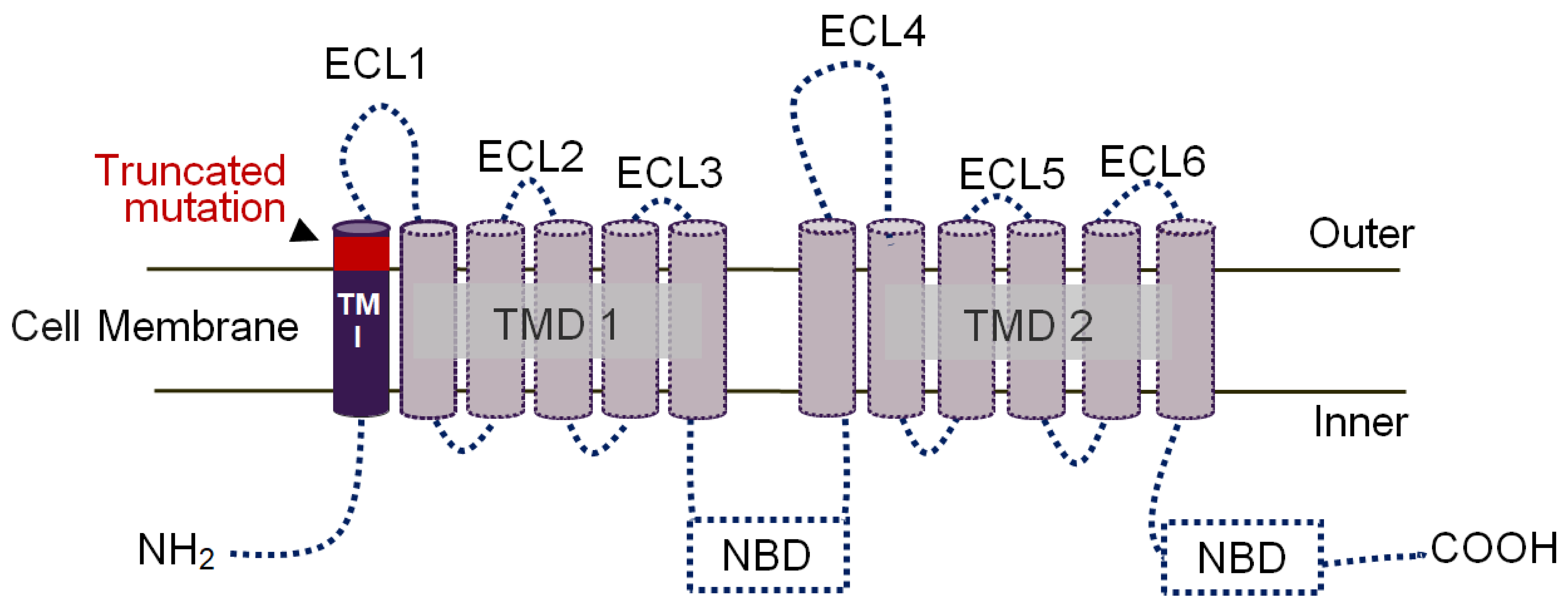

D

Wild   CTGGTTCCGTTTACACTAGCATTGTTTATCGGATATTTCTCTGGAGAAAAATCACCTGAA
Leu Val Pro Phe Thr Leu Ala Leu Phe Ile Gly Tyr Phe Ser Gly Glu Lys Ser Pro Glu

C4T02   CTGGTTCCGTTTACACT---------TGTTTATCGGATATTTCTCTGGAGAAAAATCACCTGAA   (-5nt)

Supplementary Figure 2.

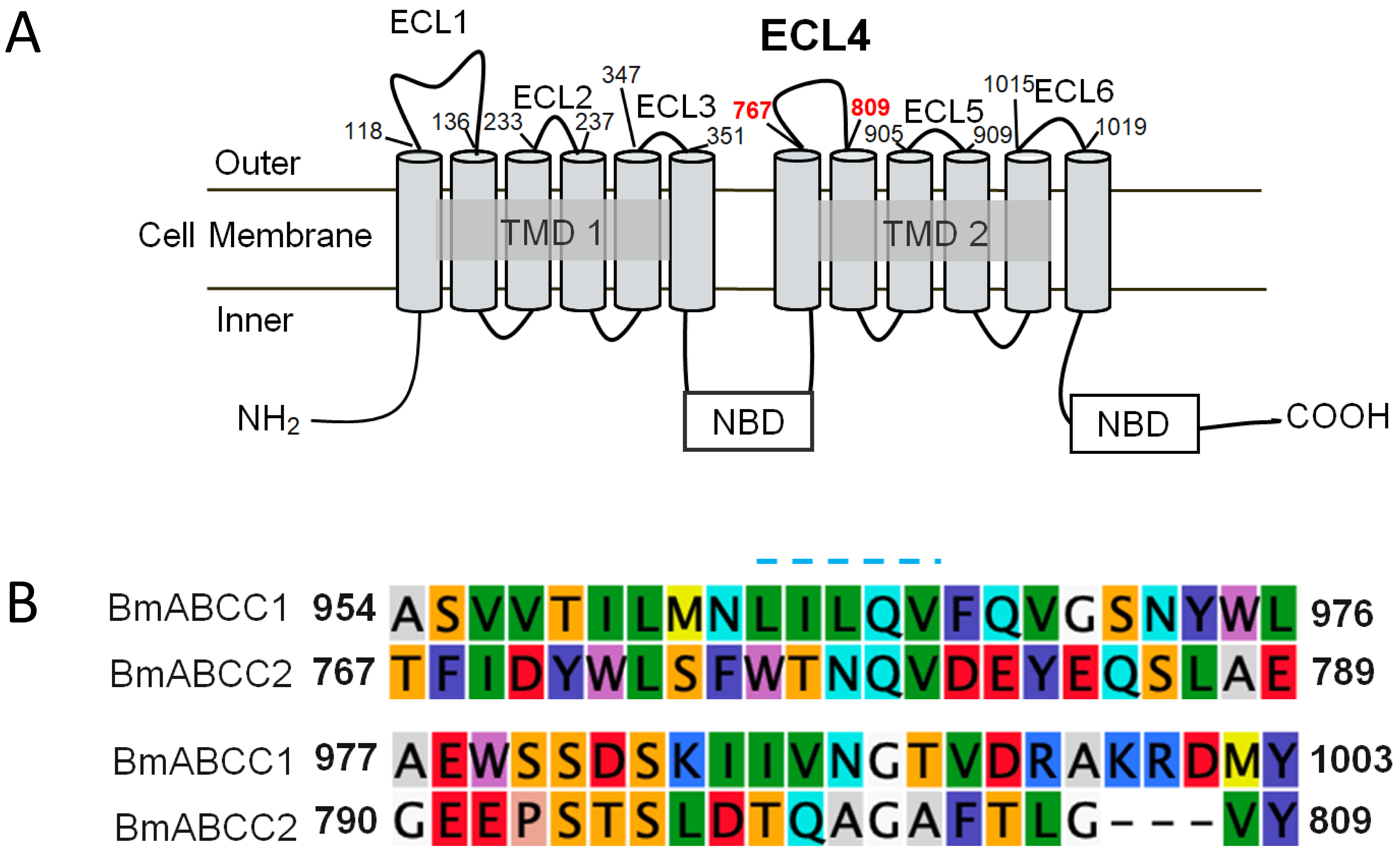

Supplementary Figure 3.

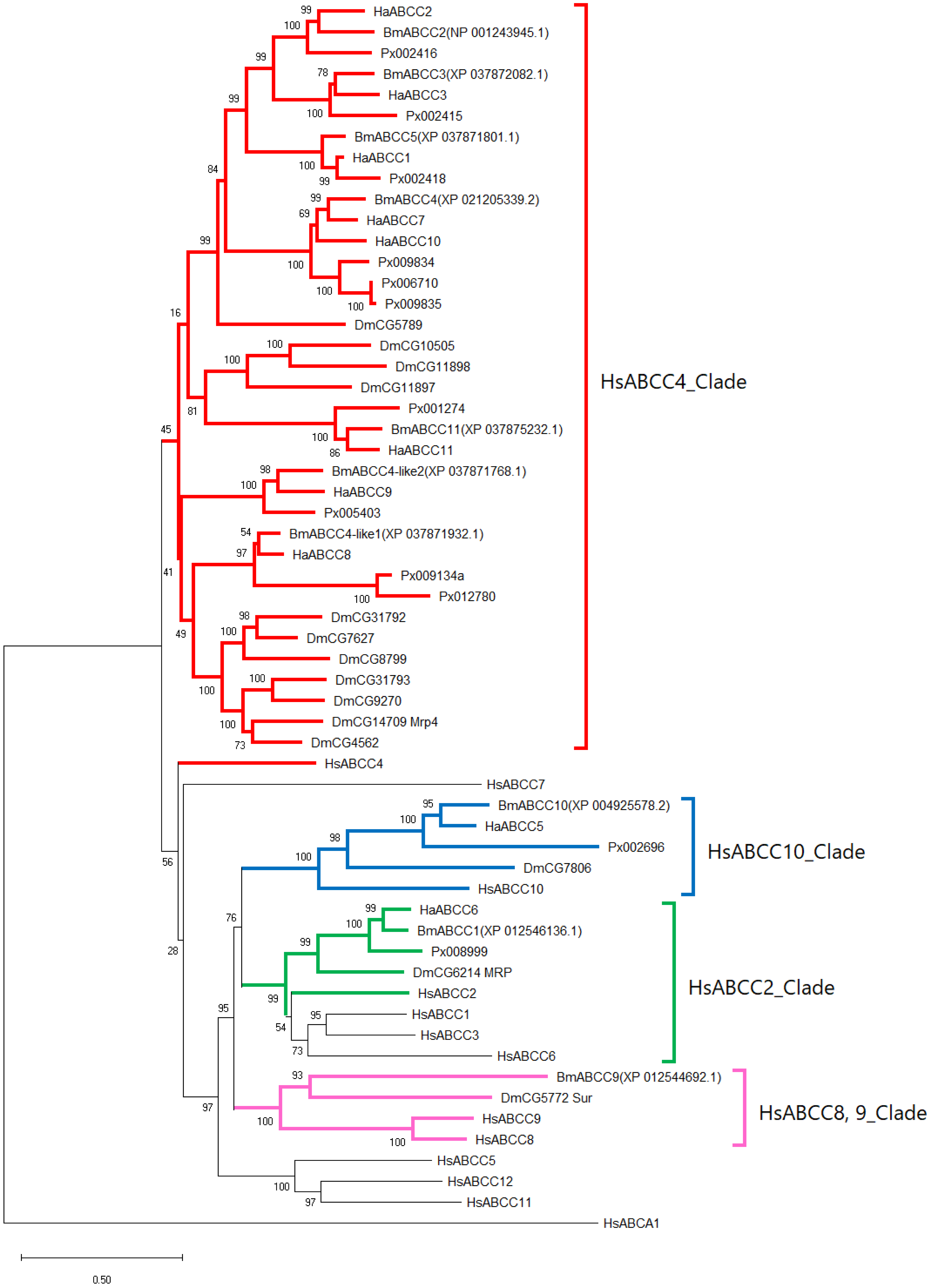

Supplementary Figure 5.

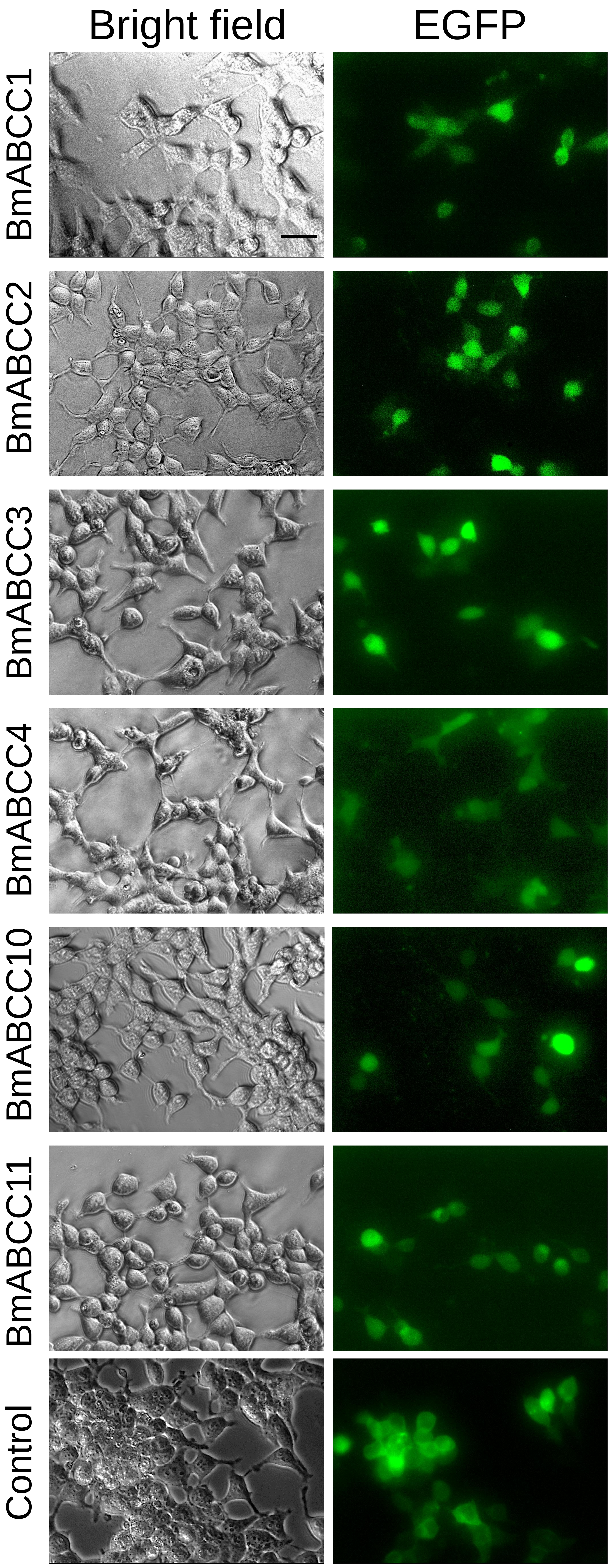

Supplementary Figure 6.

A

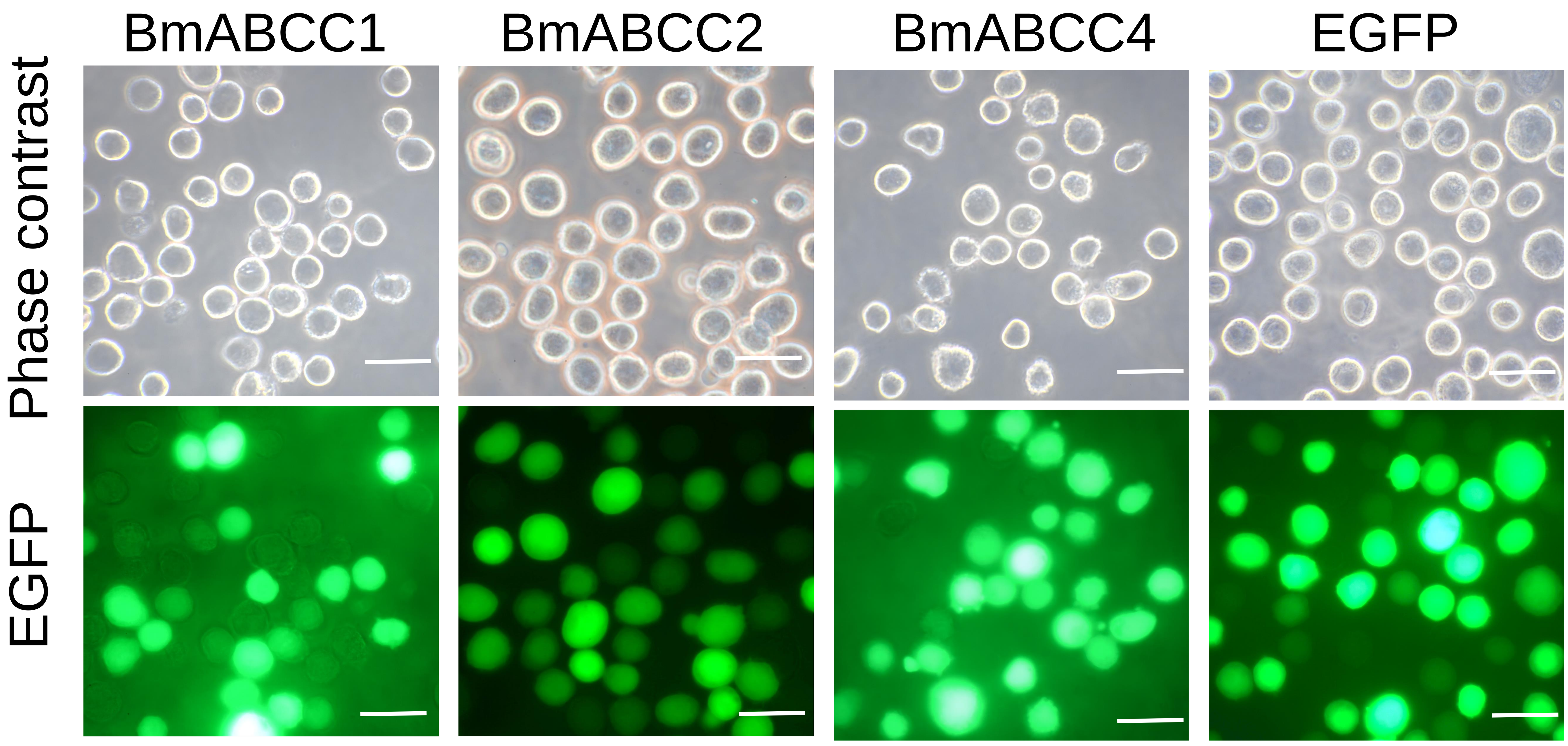

Supplementary Figure 7.

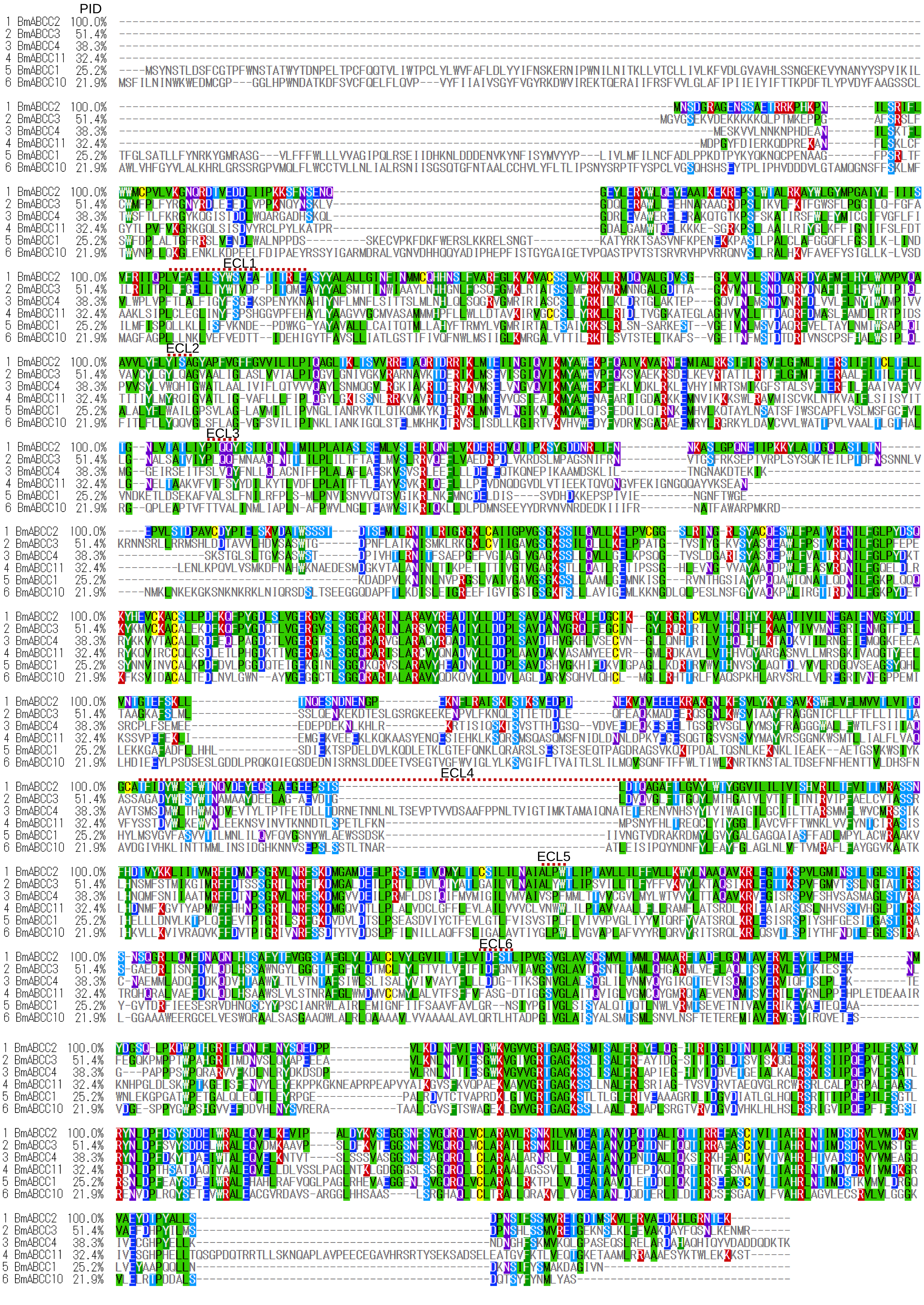

Supplementary Figure 8.
